# Study of the doubly charmed tetraquark ${{{{{{\rm{T}}}}}}}_{{{{{{\rm{c}}}}}}{{{{{\rm{c}}}}}}}^{+}$

**DOI:** 10.1038/s41467-022-30206-w

**Published:** 2022-06-16

**Authors:** LHCb collaboration, R. Aaij, A. S. W. Abdelmotteleb, C. Abellán Beteta, F. J. Abudinen Gallego, T. Ackernley, B. Adeva, M. Adinolfi, H. Afsharnia, C. Agapopoulou, C. A. Aidala, S. Aiola, Z. Ajaltouni, S. Akar, J. Albrecht, F. Alessio, M. Alexander, A. Alfonso Albero, Z. Aliouche, G. Alkhazov, P. Alvarez Cartelle, S. Amato, J. L. Amey, Y. Amhis, L. An, L. Anderlini, A. Andreianov, M. Andreotti, F. Archilli, A. Artamonov, M. Artuso, K. Arzymatov, E. Aslanides, M. Atzeni, B. Audurier, S. Bachmann, M. Bachmayer, J. J. Back, P. Baladron Rodriguez, V. Balagura, W. Baldini, J. Baptista Leite, M. Barbetti, R. J. Barlow, S. Barsuk, W. Barter, M. Bartolini, F. Baryshnikov, J. M. Basels, S. Bashir, G. Bassi, B. Batsukh, A. Battig, A. Bay, A. Beck, M. Becker, F. Bedeschi, I. Bediaga, A. Beiter, V. Belavin, S. Belin, V. Bellee, K. Belous, I. Belov, I. Belyaev, G. Bencivenni, E. Ben-Haim, A. Berezhnoy, R. Bernet, D. Berninghoff, H. C. Bernstein, C. Bertella, A. Bertolin, C. Betancourt, F. Betti, Ia Bezshyiko, S. Bhasin, J. Bhom, L. Bian, M. S. Bieker, S. Bifani, P. Billoir, M. Birch, F. C. R. Bishop, A. Bitadze, A. Bizzeti, M. Bjørn, M. P. Blago, T. Blake, F. Blanc, S. Blusk, D. Bobulska, J. A. Boelhauve, O. Boente Garcia, T. Boettcher, A. Boldyrev, A. Bondar, N. Bondar, S. Borghi, M. Borisyak, M. Borsato, J. T. Borsuk, S. A. Bouchiba, T. J. V. Bowcock, A. Boyer, C. Bozzi, M. J. Bradley, S. Braun, A. Brea Rodriguez, J. Brodzicka, A. Brossa Gonzalo, D. Brundu, A. Buonaura, L. Buonincontri, A. T. Burke, C. Burr, A. Bursche, A. Butkevich, J. S. Butter, J. Buytaert, W. Byczynski, S. Cadeddu, H. Cai, R. Calabrese, L. Calefice, L. Calero Diaz, S. Cali, R. Calladine, M. Calvi, M. Calvo Gomez, P. Camargo Magalhaes, P. Campana, A. F. Campoverde Quezada, S. Capelli, L. Capriotti, A. Carbone, G. Carboni, R. Cardinale, A. Cardini, I. Carli, P. Carniti, L. Carus, K. Carvalho Akiba, A. Casais Vidal, G. Casse, M. Cattaneo, G. Cavallero, S. Celani, J. Cerasoli, D. Cervenkov, A. J. Chadwick, M. G. Chapman, M. Charles, Ph Charpentier, G. Chatzikonstantinidis, C. A. Chavez Barajas, M. Chefdeville, C. Chen, S. Chen, A. Chernov, V. Chobanova, S. Cholak, M. Chrzaszcz, A. Chubykin, V. Chulikov, P. Ciambrone, M. F. Cicala, X. Cid Vidal, G. Ciezarek, P. E. L. Clarke, M. Clemencic, H. V. Cliff, J. Closier, J. L. Cobbledick, V. Coco, J. A. B. Coelho, J. Cogan, E. Cogneras, L. Cojocariu, P. Collins, T. Colombo, L. Congedo, A. Contu, N. Cooke, G. Coombs, I. Corredoira, G. Corti, C. M. Costa Sobral, B. Couturier, D. C. Craik, J. Crkovská, M. Cruz Torres, R. Currie, C. L. Da Silva, S. Dadabaev, L. Dai, E. Dall’Occo, J. Dalseno, C. D’Ambrosio, A. Danilina, P. d’Argent, J. E. Davies, A. Davis, O. De Aguiar Francisco, K. De Bruyn, S. De Capua, M. De Cian, J. M. De Miranda, L. De Paula, M. De Serio, D. De Simone, P. De Simone, F. De Vellis, J. A. de Vries, C. T. Dean, F. Debernardis, D. Decamp, V. Dedu, L. Del Buono, B. Delaney, H.-P. Dembinski, A. Dendek, V. Denysenko, D. Derkach, O. Deschamps, F. Desse, F. Dettori, B. Dey, A. Di Cicco, P. Di Nezza, S. Didenko, L. Dieste Maronas, H. Dijkstra, V. Dobishuk, C. Dong, A. M. Donohoe, F. Dordei, A. C. dos Reis, L. Douglas, A. Dovbnya, A. G. Downes, M. W. Dudek, L. Dufour, V. Duk, P. Durante, J. M. Durham, D. Dutta, A. Dziurda, A. Dzyuba, S. Easo, U. Egede, V. Egorychev, S. Eidelman, S. Eisenhardt, S. Ek-In, L. Eklund, S. Ely, A. Ene, E. Epple, S. Escher, J. Eschle, S. Esen, T. Evans, A. Falabella, J. Fan, Y. Fan, B. Fang, S. Farry, D. Fazzini, M. Féo, A. Fernandez Prieto, A. D. Fernez, F. Ferrari, L. Ferreira Lopes, F. Ferreira Rodrigues, S. Ferreres Sole, M. Ferrillo, M. Ferro-Luzzi, S. Filippov, R. A. Fini, M. Fiorini, M. Firlej, K. M. Fischer, D. S. Fitzgerald, C. Fitzpatrick, T. Fiutowski, A. Fkiaras, F. Fleuret, M. Fontana, F. Fontanelli, R. Forty, D. Foulds-Holt, V. Franco Lima, M. Franco Sevilla, M. Frank, E. Franzoso, G. Frau, C. Frei, D. A. Friday, J. Fu, Q. Fuehring, E. Gabriel, G. Galati, A. Gallas Torreira, D. Galli, S. Gambetta, Y. Gan, M. Gandelman, P. Gandini, Y. Gao, M. Garau, L. M. Garcia Martin, P. Garcia Moreno, J. García Pardiñas, B. Garcia Plana, F. A. Garcia Rosales, L. Garrido, C. Gaspar, R. E. Geertsema, D. Gerick, L. L. Gerken, E. Gersabeck, M. Gersabeck, T. Gershon, D. Gerstel, L. Giambastiani, V. Gibson, H. K. Giemza, A. L. Gilman, M. Giovannetti, A. Gioventù, P. Gironella Gironell, L. Giubega, C. Giugliano, K. Gizdov, E. L. Gkougkousis, V. V. Gligorov, C. Göbel, E. Golobardes, D. Golubkov, A. Golutvin, A. Gomes, S. Gomez Fernandez, F. Goncalves Abrantes, M. Goncerz, G. Gong, P. Gorbounov, I. V. Gorelov, C. Gotti, E. Govorkova, J. P. Grabowski, T. Grammatico, L. A. Granado Cardoso, E. Graugés, E. Graverini, G. Graziani, A. Grecu, L. M. Greeven, N. A. Grieser, L. Grillo, S. Gromov, B. R. Gruberg Cazon, C. Gu, M. Guarise, M. Guittiere, P. A. Günther, E. Gushchin, A. Guth, Y. Guz, T. Gys, T. Hadavizadeh, G. Haefeli, C. Haen, J. Haimberger, T. Halewood-leagas, P. M. Hamilton, J. P. Hammerich, Q. Han, X. Han, T. H. Hancock, E. B. Hansen, S. Hansmann-Menzemer, N. Harnew, T. Harrison, C. Hasse, M. Hatch, J. He, M. Hecker, K. Heijhoff, K. Heinicke, A. M. Hennequin, K. Hennessy, L. Henry, J. Heuel, A. Hicheur, D. Hill, M. Hilton, S. E. Hollitt, R. Hou, Y. Hou, J. Hu, J. Hu, W. Hu, X. Hu, W. Huang, X. Huang, W. Hulsbergen, R. J. Hunter, M. Hushchyn, D. Hutchcroft, D. Hynds, P. Ibis, M. Idzik, D. Ilin, P. Ilten, A. Inglessi, A. Ishteev, K. Ivshin, R. Jacobsson, H. Jage, S. Jakobsen, E. Jans, B. K. Jashal, A. Jawahery, V. Jevtic, F. Jiang, M. John, D. Johnson, C. R. Jones, T. P. Jones, B. Jost, N. Jurik, S. H. Kalavan Kadavath, S. Kandybei, Y. Kang, M. Karacson, M. Karpov, F. Keizer, D. M. Keller, M. Kenzie, T. Ketel, B. Khanji, A. Kharisova, S. Kholodenko, T. Kirn, V. S. Kirsebom, O. Kitouni, S. Klaver, N. Kleijne, K. Klimaszewski, M. R. Kmiec, S. Koliiev, A. Kondybayeva, A. Konoplyannikov, P. Kopciewicz, R. Kopecna, P. Koppenburg, M. Korolev, I. Kostiuk, O. Kot, S. Kotriakhova, P. Kravchenko, L. Kravchuk, R. D. Krawczyk, M. Kreps, F. Kress, S. Kretzschmar, P. Krokovny, W. Krupa, W. Krzemien, M. Kucharczyk, V. Kudryavtsev, H. S. Kuindersma, G. J. Kunde, T. Kvaratskheliya, D. Lacarrere, G. Lafferty, A. Lai, A. Lampis, D. Lancierini, J. J. Lane, R. Lane, G. Lanfranchi, C. Langenbruch, J. Langer, O. Lantwin, T. Latham, F. Lazzari, R. Le Gac, S. H. Lee, R. Lefèvre, A. Leflat, S. Legotin, O. Leroy, T. Lesiak, B. Leverington, H. Li, P. Li, S. Li, Y. Li, Z. Li, X. Liang, T. Lin, R. Lindner, V. Lisovskyi, R. Litvinov, G. Liu, H. Liu, Q. Liu, S. Liu, A. Lobo Salvia, A. Loi, J. Lomba Castro, I. Longstaff, J. H. Lopes, S. Lopez Solino, G. H. Lovell, Y. Lu, C. Lucarelli, D. Lucchesi, S. Luchuk, M. Lucio Martinez, V. Lukashenko, Y. Luo, A. Lupato, E. Luppi, O. Lupton, A. Lusiani, X. Lyu, L. Ma, R. Ma, S. Maccolini, F. Machefert, F. Maciuc, V. Macko, P. Mackowiak, S. Maddrell-Mander, O. Madejczyk, L. R. Madhan Mohan, O. Maev, A. Maevskiy, D. Maisuzenko, M. W. Majewski, J. J. Malczewski, S. Malde, B. Malecki, A. Malinin, T. Maltsev, H. Malygina, G. Manca, G. Mancinelli, D. Manuzzi, D. Marangotto, J. Maratas, J. F. Marchand, U. Marconi, S. Mariani, C. Marin Benito, M. Marinangeli, J. Marks, A. M. Marshall, P. J. Marshall, G. Martelli, G. Martellotti, L. Martinazzoli, M. Martinelli, D. Martinez Santos, F. Martinez Vidal, A. Massafferri, M. Materok, R. Matev, A. Mathad, V. Matiunin, C. Matteuzzi, K. R. Mattioli, A. Mauri, E. Maurice, J. Mauricio, M. Mazurek, M. McCann, L. Mcconnell, T. H. Mcgrath, N. T. Mchugh, A. McNab, R. McNulty, J. V. Mead, B. Meadows, G. Meier, N. Meinert, D. Melnychuk, S. Meloni, M. Merk, A. Merli, L. Meyer Garcia, M. Mikhasenko, D. A. Milanes, E. Millard, M. Milovanovic, M.-N. Minard, A. Minotti, L. Minzoni, S. E. Mitchell, B. Mitreska, D. S. Mitzel, A. Mödden, R. A. Mohammed, R. D. Moise, S. Mokhnenko, T. Mombächer, I. A. Monroy, S. Monteil, M. Morandin, G. Morello, M. J. Morello, J. Moron, A. B. Morris, A. G. Morris, R. Mountain, H. Mu, F. Muheim, M. Mulder, D. Müller, K. Müller, C. H. Murphy, D. Murray, P. Muzzetto, P. Naik, T. Nakada, R. Nandakumar, T. Nanut, I. Nasteva, M. Needham, I. Neri, N. Neri, S. Neubert, N. Neufeld, R. Newcombe, E. M. Niel, S. Nieswand, N. Nikitin, N. S. Nolte, C. Normand, C. Nunez, A. Oblakowska-Mucha, V. Obraztsov, T. Oeser, D. P. O’Hanlon, S. Okamura, R. Oldeman, F. Oliva, M. E. Olivares, C. J. G. Onderwater, R. H. O’neil, J. M. Otalora Goicochea, T. Ovsiannikova, P. Owen, A. Oyanguren, K. O. Padeken, B. Pagare, P. R. Pais, T. Pajero, A. Palano, M. Palutan, Y. Pan, G. Panshin, A. Papanestis, M. Pappagallo, L. L. Pappalardo, C. Pappenheimer, W. Parker, C. Parkes, B. Passalacqua, G. Passaleva, A. Pastore, M. Patel, C. Patrignani, C. J. Pawley, A. Pearce, A. Pellegrino, M. Pepe Altarelli, S. Perazzini, D. Pereima, A. Pereiro Castro, P. Perret, M. Petric, K. Petridis, A. Petrolini, A. Petrov, S. Petrucci, M. Petruzzo, T. T. H. Pham, L. Pica, M. Piccini, B. Pietrzyk, G. Pietrzyk, M. Pili, D. Pinci, F. Pisani, M. Pizzichemi, P. K. Resmi, V. Placinta, J. Plews, M. Plo Casasus, F. Polci, M. Poli Lener, M. Poliakova, A. Poluektov, N. Polukhina, I. Polyakov, E. Polycarpo, S. Ponce, D. Popov, S. Popov, S. Poslavskii, K. Prasanth, L. Promberger, C. Prouve, V. Pugatch, V. Puill, H. Pullen, G. Punzi, H. Qi, W. Qian, J. Qin, N. Qin, R. Quagliani, B. Quintana, N. V. Raab, R. I. Rabadan Trejo, B. Rachwal, J. H. Rademacker, M. Rama, M. Ramos Pernas, M. S. Rangel, F. Ratnikov, G. Raven, M. Reboud, F. Redi, F. Reiss, C. Remon Alepuz, Z. Ren, V. Renaudin, R. Ribatti, S. Ricciardi, K. Rinnert, P. Robbe, G. Robertson, A. B. Rodrigues, E. Rodrigues, J. A. Rodriguez Lopez, E. R. R. Rodriguez Rodriguez, A. Rollings, P. Roloff, V. Romanovskiy, M. Romero Lamas, A. Romero Vidal, J. D. Roth, M. Rotondo, M. S. Rudolph, T. Ruf, R. A. Ruiz Fernandez, J. Ruiz Vidal, A. Ryzhikov, J. Ryzka, J. J. Saborido Silva, N. Sagidova, N. Sahoo, B. Saitta, M. Salomoni, C. Sanchez Gras, R. Santacesaria, C. Santamarina Rios, M. Santimaria, E. Santovetti, D. Saranin, G. Sarpis, M. Sarpis, A. Sarti, C. Satriano, A. Satta, M. Saur, D. Savrina, H. Sazak, L. G. Scantlebury Smead, A. Scarabotto, S. Schael, S. Scherl, M. Schiller, H. Schindler, M. Schmelling, B. Schmidt, S. Schmitt, O. Schneider, A. Schopper, M. Schubiger, S. Schulte, M. H. Schune, R. Schwemmer, B. Sciascia, S. Sellam, A. Semennikov, M. Senghi Soares, A. Sergi, N. Serra, L. Sestini, A. Seuthe, Y. Shang, D. M. Shangase, M. Shapkin, I. Shchemerov, L. Shchutska, T. Shears, L. Shekhtman, Z. Shen, V. Shevchenko, E. B. Shields, Y. Shimizu, E. Shmanin, J. D. Shupperd, B. G. Siddi, R. Silva Coutinho, G. Simi, S. Simone, N. Skidmore, T. Skwarnicki, M. W. Slater, I. Slazyk, J. C. Smallwood, J. G. Smeaton, A. Smetkina, E. Smith, M. Smith, A. Snoch, M. Soares, L. Soares Lavra, M. D. Sokoloff, F. J. P. Soler, A. Solovev, I. Solovyev, F. L. Souza De Almeida, B. Souza De Paula, B. Spaan, E. Spadaro Norella, P. Spradlin, F. Stagni, M. Stahl, S. Stahl, S. Stanislaus, O. Steinkamp, O. Stenyakin, H. Stevens, S. Stone, M. Straticiuc, D. Strekalina, F. Suljik, J. Sun, L. Sun, Y. Sun, P. Svihra, P. N. Swallow, K. Swientek, A. Szabelski, T. Szumlak, M. Szymanski, S. Taneja, A. R. Tanner, M. D. Tat, A. Terentev, F. Teubert, E. Thomas, D. J. D. Thompson, K. A. Thomson, V. Tisserand, S. T’Jampens, M. Tobin, L. Tomassetti, X. Tong, D. Torres Machado, D. Y. Tou, E. Trifonova, C. Trippl, G. Tuci, A. Tully, N. Tuning, A. Ukleja, D. J. Unverzagt, E. Ursov, A. Usachov, A. Ustyuzhanin, U. Uwer, A. Vagner, V. Vagnoni, A. Valassi, G. Valenti, N. Valls Canudas, M. van Beuzekom, M. Van Dijk, E. van Herwijnen, C. B. Van Hulse, M. van Veghel, R. Vazquez Gomez, P. Vazquez Regueiro, C. Vázquez Sierra, S. Vecchi, J. J. Velthuis, M. Veltri, A. Venkateswaran, M. Veronesi, M. Vesterinen, D. Vieira, M. Vieites Diaz, H. Viemann, X. Vilasis-Cardona, E. Vilella Figueras, A. Villa, P. Vincent, F. C. Volle, D. Vom Bruch, A. Vorobyev, V. Vorobyev, N. Voropaev, K. Vos, R. Waldi, J. Walsh, C. Wang, J. Wang, J. Wang, J. Wang, J. Wang, M. Wang, R. Wang, Y. Wang, Z. Wang, Z. Wang, Z. Wang, J. A. Ward, N. K. Watson, S. G. Weber, D. Websdale, C. Weisser, B. D. C. Westhenry, D. J. White, M. Whitehead, A. R. Wiederhold, D. Wiedner, G. Wilkinson, M. Wilkinson, I. Williams, M. Williams, M. R. J. Williams, F. F. Wilson, W. Wislicki, M. Witek, L. Witola, G. Wormser, S. A. Wotton, H. Wu, K. Wyllie, Z. Xiang, D. Xiao, Y. Xie, A. Xu, J. Xu, L. Xu, M. Xu, Q. Xu, Z. Xu, Z. Xu, D. Yang, S. Yang, Y. Yang, Z. Yang, Z. Yang, Y. Yao, L. E. Yeomans, H. Yin, J. Yu, X. Yuan, O. Yushchenko, E. Zaffaroni, M. Zavertyaev, M. Zdybal, O. Zenaiev, M. Zeng, D. Zhang, L. Zhang, S. Zhang, S. Zhang, Y. Zhang, Y. Zhang, A. Zharkova, A. Zhelezov, Y. Zheng, T. Zhou, X. Zhou, Y. Zhou, V. Zhovkovska, X. Zhu, X. Zhu, Z. Zhu, V. Zhukov, J. B. Zonneveld, Q. Zou, S. Zucchelli, D. Zuliani, G. Zunica

**Affiliations:** 1 Nikhef National Institute for Subatomic Physics, Amsterdam, Netherlands; 2 Department of Physics, University of Warwick, Coventry, UK; 3 Physik-Institut, Universität Zürich, Zürich, Switzerland; 4 Oliver Lodge Laboratory, University of Liverpool, Liverpool, UK; 5 Instituto Galego de Física de Altas Enerxías (IGFAE), Universidade de Santiago de Compostela, Santiago de Compostela, Spain; 6 H.H. Wills Physics Laboratory, University of Bristol, Bristol, UK; 7 Université Clermont Auvergne, CNRS/IN2P3, LPC, Clermont-Ferrand, France; 8 LPNHE, Sorbonne Université, Paris Diderot Sorbonne Paris Cité, CNRS/IN2P3, Paris, France; 9 University of Michigan, Ann Arbor, USA; 10 INFN Sezione di Milano, Milano, Italy; 11 University of Cincinnati, Cincinnati, OH USA; 12 Fakultät Physik, Technische Universität Dortmund, Dortmund, Germany; 13 European Organization for Nuclear Research (CERN), Geneva, Switzerland; 14 School of Physics and Astronomy, University of Glasgow, Glasgow, UK; 15 ICCUB, Universitat de Barcelona, Barcelona, Spain; 16 Department of Physics and Astronomy, University of Manchester, Manchester, UK; 17 Petersburg Nuclear Physics Institute NRC Kurchatov Institute (PNPI NRC KI), Gatchina, Russia; 18 Cavendish Laboratory, University of Cambridge, Cambridge, UK; 19 Universidade Federal do Rio de Janeiro (UFRJ), Rio de Janeiro, Brazil; 20 Université Paris-Saclay, CNRS/IN2P3, IJCLab, Orsay, France; 21 Q30265285 INFN Sezione di Firenze, Firenze, Italy; 22 INFN Sezione di Ferrara, Ferrara, Italy; 23 Physikalisches Institut, Ruprecht-Karls-Universität Heidelberg, Heidelberg, Germany; 24 Institute for High Energy Physics NRC Kurchatov Institute (IHEP NRC KI), Protvino, Russia, Protvino, Russia; 25 Syracuse University, Syracuse, NY USA; 26 Yandex School of Data Analysis, Moscow, Russia; 27 Aix Marseille Univ, CNRS/IN2P3, CPPM, Marseille, France; 28 Laboratoire Leprince-Ringuet, CNRS/IN2P3, Ecole Polytechnique, Institut Polytechnique de Paris, Palaiseau, France; 29 Institute of Physics, Ecole Polytechnique Fédérale de Lausanne (EPFL), Lausanne, Switzerland; 30 Centro Brasileiro de Pesquisas Físicas (CBPF), Rio de Janeiro, Brazil; 31 Università di Firenze, Firenze, Italy; 32 Imperial College London, London, UK; 33 INFN Sezione di Genova, Genova, Italy; 34 Università di Genova, Genova, Italy; 35 National University of Science and Technology “MISIS”, Moscow, Russia; 36 I. Physikalisches Institut, RWTH Aachen University, Aachen, Germany; 37 AGH - University of Science and Technology, Faculty of Physics and Applied Computer Science, Kraków, Poland; 38 INFN Sezione di Pisa, Pisa, Italy; 39 INFN Sezione di Cagliari, Monserrato, Italy; 40 Institute of Nuclear Physics, Moscow State University (SINP MSU), Moscow, Russia; 41 Institute of Theoretical and Experimental Physics NRC Kurchatov Institute (ITEP NRC KI), Moscow, Russia; 42 INFN Laboratori Nazionali di Frascati, Frascati, Italy; 43 Universita degli Studi di Padova, Universita e INFN, Padova, Padova, Italy; 44 Henryk Niewodniczanski Institute of Nuclear Physics Polish Academy of Sciences, Kraków, Poland; 45 School of Physics and Technology, Wuhan University, Wuhan, China; 46 University of Birmingham, Birmingham, UK; 47 Università di Modena e Reggio Emilia, Modena, Italy; 48 Department of Physics, University of Oxford, Oxford, UK; 49 National Research University Higher School of Economics, Moscow, Russia; 50 Budker Institute of Nuclear Physics (SB RAS), Novosibirsk, Russia; 51 University of Maryland, College Park, MD USA; 52 Guangdong Provincial Key Laboratory of Nuclear Science, Guangdong-Hong Kong Joint Laboratory of Quantum Matter, Institute of Quantum Matter, South China Normal University, Guangzhou, China; 53 Institute for Nuclear Research of the Russian Academy of Sciences (INR RAS), Moscow, Russia; 54 Università di Ferrara, Ferrara, Italy; 55 INFN Sezione di Milano-Bicocca, Milano, Italy; 56 Università di Milano Bicocca, Milano, Italy; 57 DS4DS, La Salle, Universitat Ramon Llull, Barcelona, Spain; 58 University of Chinese Academy of Sciences, Beijing, China; 59 INFN Sezione di Bologna, Bologna, Italy; 60 Università di Bologna, Bologna, Italy; 61 INFN Sezione di Roma Tor Vergata, Roma, Italy; 62 Institute Of High Energy Physics (IHEP), Beijing, China; 63 Univ. Savoie Mont Blanc, CNRS, IN2P3-LAPP, Annecy, France; 64 Center for High Energy Physics, Tsinghua University, Beijing, China; 65 School of Physics and Astronomy, University of Edinburgh, Edinburgh, UK; 66 Horia Hulubei National Institute of Physics and Nuclear Engineering, Bucharest-Magurele, Romania; 67 INFN Sezione di Bari, Bari, Italy; 68 Università di Bari, Bari, Italy; 69 Massachusetts Institute of Technology, Cambridge, MA USA; 70 Los Alamos National Laboratory (LANL), Los Alamos, USA; 71 Physics and Micro Electronic College, Hunan University, Changsha City, China; 72 Van Swinderen Institute, University of Groningen, Groningen, Netherlands; 73 Universiteit Maastricht, Maastricht, Netherlands; 74 Università di Cagliari, Cagliari, Italy; 75 Eotvos Lorand University, Budapest, Hungary; 76 Institute for Nuclear Research of the National Academy of Sciences (KINR), Kyiv, Ukraine; 77 School of Physics, University College Dublin, Dublin, Ireland; 78 NSC Kharkiv Institute of Physics and Technology (NSC KIPT), Kharkiv, Ukraine; 79 INFN Sezione di Perugia, Perugia, Italy; 80 STFC Rutherford Appleton Laboratory, Didcot, UK; 81 School of Physics and Astronomy, Monash University, Melbourne, Australia; 82 Novosibirsk State University, Novosibirsk, Russia; 83 Department of Physics and Astronomy, Uppsala University, Uppsala, Sweden; 84 School of Physics State Key Laboratory of Nuclear Physics and Technology, Peking University, Beijing, China; 85 National Center for Nuclear Research (NCBJ), Warsaw, Poland; 86 Università di Roma Tor Vergata, Roma, Italy; 87 Pontifícia Universidade Católica do Rio de Janeiro (PUC-Rio), Rio de Janeiro, Brazil; 88 Universidade Federal do Triângulo Mineiro (UFTM), Uberaba-MG, Uberaba, Brazil; 89 Institute of Particle Physics, Central China Normal University, Wuhan, Hubei China; 90 Hangzhou Institute for Advanced Study, UCAS, Hangzhou, China; 91 Instituto de Fisica Corpuscular, Centro Mixto Universidad de Valencia - CSIC, Valencia, Spain; 92 Nikhef National Institute for Subatomic Physics and VU University Amsterdam, Amsterdam, Netherlands; 93 National Research Tomsk Polytechnic University, Tomsk, Russia; 94 Università di Siena, Siena, Italy; 95 Università di Padova, Padova, Italy; 96 Scuola Normale Superiore, Pisa, Italy; 97 National Research Centre Kurchatov Institute, Moscow, Russia; 98 Università degli Studi di Milano, Milano, Italy; 99 MSU - Iligan Institute of Technology (MSU-IIT), Iligan, Philippines; 100 INFN Sezione di Roma La Sapienza, Roma, Italy; 101 Institut für Physik, Universität Rostock, Rostock, Germany; 102 Departamento de Fisica, Universidad Nacional de Colombia, Bogota, Colombia; 103 Universität Bonn - Helmholtz-Institut für Strahlen und Kernphysik, Bonn, Germany; 104 P.N. Lebedev Physical Institute, Russian Academy of Science (LPI RAS), Moscow, Russia; 105 Università di Pisa, Pisa, Italy; 106 Università della Basilicata, Potenza, Italy; 107 Max-Planck-Institut für Kernphysik (MPIK), Heidelberg, Germany; 108 Università di Urbino, Urbino, Italy

**Keywords:** Experimental particle physics, Particle physics

## Abstract

Quantum chromodynamics, the theory of the strong force, describes interactions of coloured quarks and gluons and the formation of hadronic matter. Conventional hadronic matter consists of baryons and mesons made of three quarks and quark-antiquark pairs, respectively. Particles with an alternative quark content are known as exotic states. Here a study is reported of an exotic narrow state in the D^0^D^0^*π*^+^ mass spectrum just below the D^*+^D^0^ mass threshold produced in proton-proton collisions collected with the LHCb detector at the Large Hadron Collider. The state is consistent with the ground isoscalar ${{{{{{\rm{T}}}}}}}_{{{{{{\rm{c}}}}}}{{{{{\rm{c}}}}}}}^{+}$ tetraquark with a quark content of ${{{{{\rm{c}}}}}}{{{{{\rm{c}}}}}}\overline{{{{{{\rm{u}}}}}}}\overline{{{{{{\rm{d}}}}}}}$ and spin-parity quantum numbers J^P^ = 1^+^. Study of the DD mass spectra disfavours interpretation of the resonance as the isovector state. The decay structure via intermediate off-shell D^*+^ mesons is consistent with the observed D^0^*π*^+^ mass distribution. To analyse the mass of the resonance and its coupling to the D^*^D system, a dedicated model is developed under the assumption of an isoscalar axial-vector ${{{{{{\rm{T}}}}}}}_{{{{{{\rm{c}}}}}}{{{{{\rm{c}}}}}}}^{+}$ state decaying to the D^*^D channel. Using this model, resonance parameters including the pole position, scattering length, effective range and compositeness are determined to reveal important information about the nature of the ${{{{{{\rm{T}}}}}}}_{{{{{{\rm{c}}}}}}{{{{{\rm{c}}}}}}}^{+}$ state. In addition, an unexpected dependence of the production rate on track multiplicity is observed.

## Abstract (web summary)

The existence and properties of tetraquark states with two heavy quarks and two light antiquarks have been widely debated. Here, the authors use a unitarized model to study the properties of an exotic narrow state compatible with a doubly charmed tetraquark.

## Introduction

Hadrons with quark content other than that seen in mesons (${{{{{{\rm{q}}}}}}}_{1}{\bar{{{{{{\rm{q}}}}}}}}_{2}$) and baryons (q_1_q_2_q_3_) have been actively discussed since the birth of the quark model^1–8^. Since the discovery of the *χ*_c1_(3872) state^9^ many tetraquark and pentaquark candidates, listed in Table 1, have been observed^10–19^. For all but the X_0_(2900) and X_1_(2900) states the minimal quark content implies the presence of either a ${{{{{\rm{c}}}}}}\overline{{{{{{\rm{c}}}}}}}$ or ${{{{{\rm{b}}}}}}\overline{{{{{{\rm{b}}}}}}}$ quark-antiquark pair. The masses of many tetra- and pentaquark states are close to mass thresholds, e.g., ${{{{{{\rm{D}}}}}}}^{(* )}{\overline{{{{{{\rm{D}}}}}}}}^{(* )}$ or ${{{{{{\rm{B}}}}}}}^{(* )}{\overline{{{{{{\rm{B}}}}}}}}^{(* )}$, where D^(*)^ or B^(*)^ represents a hadron containing a charm or beauty quark, respectively. Therefore, these states are likely to be hadronic molecules^16,20–22^ where colour-singlet hadrons are bound by residual nuclear forces, such as the exchange of a pion or *ρ* meson^23^, similar to electromagnetic van der Waals forces attracting electrically neutral atoms and molecules. These states are expected to have a spatial extension significantly larger than a typical compact hadron. Conversely, the only hadron currently observed that contains a pair of c quarks is the ${{{\Xi }}}_{{{{{{\rm{cc}}}}}}}^{++}$ (ccu) baryon, a long-lived, weakly-decaying compact object^24,25^. The recently observed X(6900) structure in the J/*ψ*J/*ψ* mass spectrum^26^ belongs to both categories simultaneously. Its proximity to the *χ*_c0_*χ*_c1_ threshold could indicate a molecular structure^27,28^. Alternatively, it could be a compact object, where all four quarks are within one confinement volume and each quark interacts directly with the other three quarks via the strong force^29–32^.

**Table 1:** Tetra- and pentaquark candidates and their plausible valence quark content.

States	Quark content
X_0_(2900), X_1_(2900)^148,149^	$\overline{{{{{{\rm{c}}}}}}}{{{{{\rm{d}}}}}}{{{{{\rm{u}}}}}}\overline{{{{{{\rm{s}}}}}}}$
*χ*_c1_(3872)^9^	${{{{{\rm{c}}}}}}\overline{{{{{{\rm{c}}}}}}}{{{{{\rm{q}}}}}}\overline{{{{{{\rm{q}}}}}}}$
Z_c_(3900)^150–154^, Z_c_(4020)^155,156^, Z_c_(4050)^157^, X(4100)^158^, Z_c_(4200)^159^, Z_c_(4430)^160–163^, R_c0_(4240)^162^	${{{{{\rm{c}}}}}}\overline{{{{{{\rm{c}}}}}}}{{{{{\rm{u}}}}}}\overline{{{{{{\rm{d}}}}}}}$
Z_cs_(3985)^164^, Z_cs_(4000), Z_cs_(4220)^165^	${{{{{\rm{c}}}}}}\overline{{{{{{\rm{c}}}}}}}{{{{{\rm{u}}}}}}\overline{{{{{{\rm{s}}}}}}}$
*χ*_c1_(4140)^166–169^, *χ*_c1_(4274), *χ*_c0_(4500), *χ*_c0_(4700)^169^, X(4630), X(4685)^165^, X(4740)^96^	${{{{{\rm{c}}}}}}\overline{{{{{{\rm{c}}}}}}}{{{{{\rm{s}}}}}}\overline{{{{{{\rm{s}}}}}}}$
X(6900)^26^	${{{{{\rm{c}}}}}}\overline{{{{{{\rm{c}}}}}}}{{{{{\rm{c}}}}}}\overline{{{{{{\rm{c}}}}}}}$
Z_b_(10610), Z_b_(10650)^170^	${{{{{\rm{b}}}}}}\overline{{{{{{\rm{b}}}}}}}{{{{{\rm{u}}}}}}\overline{{{{{{\rm{d}}}}}}}$
P_c_(4312)^171^, P_c_(4380)^172^, P_c_(4440), P_c_(4457)^171^, P_c_(4357)^173^	${{{{{\rm{c}}}}}}\overline{{{{{{\rm{c}}}}}}}{{{{{\rm{u}}}}}}{{{{{\rm{u}}}}}}{{{{{\rm{d}}}}}}$
P_cs_(4459)^174^	${{{{{\rm{c}}}}}}\overline{{{{{{\rm{c}}}}}}}{{{{{\rm{u}}}}}}{{{{{\rm{d}}}}}}{{{{{\rm{s}}}}}}$

The existence and properties of ${{{{{{\rm{Q}}}}}}}_{1}{{{{{{\rm{Q}}}}}}}_{2}{\bar{{{{{{\rm{q}}}}}}}}_{1}{\bar{{{{{{\rm{q}}}}}}}}_{2}$ states with two heavy quarks and two light antiquarks have been widely discussed for a long time^33–38^. In the limit of large masses of the heavy quarks the corresponding ground state should be deeply bound. In this limit, the two heavy quarks Q_1_Q_2_ form a point-like colour-antitriplet object, analogous to an antiquark, and as a result, the ${{{{{{\rm{Q}}}}}}}_{1}{{{{{{\rm{Q}}}}}}}_{2}{\bar{{{{{{\rm{q}}}}}}}}_{1}{\bar{{{{{{\rm{q}}}}}}}}_{2}$ system has similar degrees of freedom for its light quarks as an antibaryon with a single heavy quark, e.g., the ${\overline{\Lambda }}_{{{{{{\rm{c}}}}}}}^{-}$ or ${\overline{\Lambda }}_{{{{{{\rm{b}}}}}}}^{0}$ antibaryons. The beauty quark is considered heavy enough to sustain the existence of a ${{{{{\rm{b}}}}}}{{{{{\rm{b}}}}}}\overline{{{{{{\rm{u}}}}}}}\overline{{{{{{\rm{d}}}}}}}$ state that is stable with respect to the strong and electromagnetic interactions with a mass of about 200 MeV/*c*^2^ below the BB^*^ mass threshold. In the case of the ${{{{{\rm{b}}}}}}{{{{{\rm{c}}}}}}\overline{{{{{{\rm{u}}}}}}}\overline{{{{{{\rm{d}}}}}}}$ and ${{{{{\rm{c}}}}}}{{{{{\rm{c}}}}}}\overline{{{{{{\rm{u}}}}}}}\overline{{{{{{\rm{d}}}}}}}$ systems, there is currently no consensus in the literature whether such states exist and if their natural widths are narrow enough to allow for experimental observation. The theoretical predictions for the mass of the ${{\mbox{}}}{{{{{\rm{c}}}}}}{{\mbox{}}}{{{{{\rm{c}}}}}}{{\mbox{}}}\overline{{{{{{\rm{u}}}}}}}{{\mbox{}}}\overline{{{{{{\rm{d}}}}}}}{{\mbox{}}}$ ground state with spin-parity quantum numbers J^P^ = 1^+^ and isospin I = 0, denoted hereafter as ${{{{{{\rm{T}}}}}}}_{{{{{{\rm{c}}}}}}{{{{{\rm{c}}}}}}}^{+}$, relative to the D^*+^D^0^ mass threshold

1 $$\delta m\equiv {m}_{{{{{{{\rm{T}}}}}}}_{{{{{{\rm{c}}}}}}{{{{{\rm{c}}}}}}}^{+}}-\left({m}_{{{{{{{\rm{D}}}}}}}^{* +}}+{m}_{{{{{{{\rm{D}}}}}}}^{0}}\right)$$

lie in the range −300 < *δm* < 300 MeV/*c*^2 ^^39–70^, where ${m}_{{{{{{{\rm{D}}}}}}}^{* +}}$ and ${m}_{{{{{{{\rm{D}}}}}}}^{0}}$ denote the known masses of the D^*+^ and D^0^ mesons^10^, with ${{{{{\rm{c}}}}}}\overline{{{{{{\rm{d}}}}}}}$ and ${{{{{\rm{c}}}}}}\overline{{{{{{\rm{u}}}}}}}$ quark content, respectively. The observation of a narrow state in the D^0^D^0^*π*^+^ mass spectrum near the D^*+^D^0^ mass threshold, compatible with being a ${{{{{{\rm{T}}}}}}}_{{{{{{\rm{c}}}}}}{{{{{\rm{c}}}}}}}^{+}$ tetraquark state with ${{{{{\rm{c}}}}}}{{{{{\rm{c}}}}}}\overline{{{{{{\rm{u}}}}}}}\overline{{{{{{\rm{d}}}}}}}$ quark content is reported in Ref. ^71^.

In the work presented here, the properties of the ${{{{{{\rm{T}}}}}}}_{{{{{{\rm{c}}}}}}{{{{{\rm{c}}}}}}}^{+}$ state are studied by constructing a dedicated amplitude model that accounts for the D^*+^D^0^ and D^*0^D^+^ decay channels. In addition, the mass spectra of other DD^(*)^ and opposite-sign ${{{{{\rm{D}}}}}}{\overline{{{{{{\rm{D}}}}}}}}^{(* )}$ combinations are explored. Furthermore, production-related observables, such as the event multiplicity and transverse momentum (*p*_T_) spectra that are sensitive to the internal structure of the state, are discussed. This analysis is based on proton–proton (pp) collision data, corresponding to an integrated luminosity of 9 fb^−1^, collected with the LHCb detector at centre-of-mass energies of 7, 8 and 13 TeV. The LHCb detector^72,73^ is a single-arm forward spectrometer covering the pseudorapidity range 2 < *η* < 5, designed for the study of particles containing b or c quarks and is further described in Methods.

## Results

### ${{{{{{\rm{T}}}}}}}_{{{{{{\rm{c}}}}}}{{{{{\rm{c}}}}}}}^{+}$ signal in the D^0^D^0^*π*^+^ mass spectrum

**Fig. 1:**
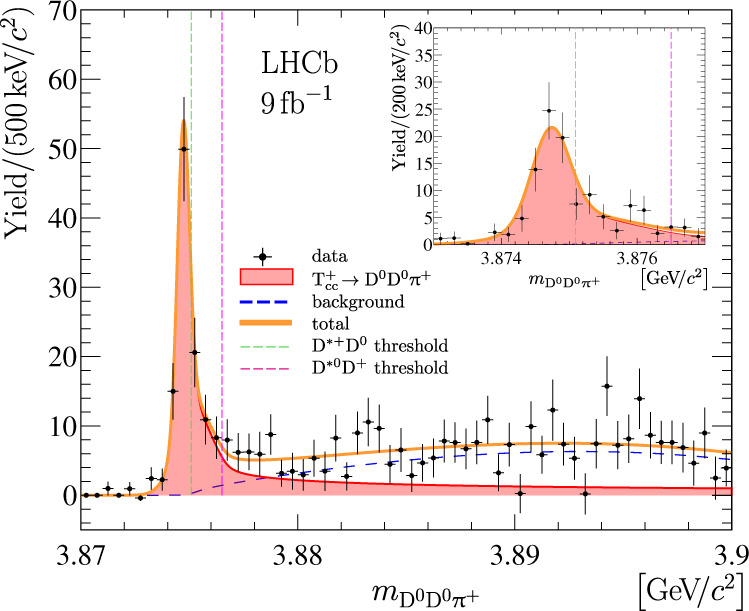
Distribution of D^0^D^0^*π*^+^ mass. Distribution of D^0^D^0^*π*^+^ mass where the contribution of the non-D^0^ background has been statistically subtracted. The result of the fit described in the text is overlaid. Uncertainties on the data points are statistical only and represent one standard deviation, calculated as a sum in quadrature of the assigned weights from the background-subtraction procedure.

The D^0^D^0^*π*^+^ final state is reconstructed using the D^0^ → K^−^*π*^+^ decay channel with two D^0^ mesons and a pion all produced promptly in the same pp collision. The inclusion of charge-conjugated processes is implied throughout the paper. The selection criteria are similar to those used in Refs. ^74–77^ and described in detail in Methods. The background not originating from true D^0^ mesons is subtracted using an extended unbinned maximum-likelihood fit to the two-dimensional distribution of the masses of the two D^0^ candidates from selected D^0^D^0^*π*^+^ combinations, see Methods and Supplementary Fig. 1a. The obtained D^0^D^0^*π*^+^ mass distribution for selected D^0^D^0^*π*^+^ combinations is shown in Fig. 1.

An extended unbinned maximum-likelihood fit to the D^0^D^0^*π*^+^ mass distribution is performed using a model consisting of signal and background components. The signal component corresponds to the ${{{{{{\rm{T}}}}}}}_{{{{{{\rm{c}}}}}}{{{{{\rm{c}}}}}}}^{+}\,\to {{{{{{\rm{D}}}}}}}^{0}{{{{{{\rm{D}}}}}}}^{0}{\pi }^{+}$ decay and is described as the convolution of the natural resonance profile with the detector mass resolution function. A relativistic P-wave two-body Breit–Wigner function ${{\mathfrak{F}}}^{{{{{{\rm{BW}}}}}}}$ with a Blatt–Weisskopf form factor^78,79^ is used in Ref. ^71^ as the natural resonance profile. That function, while sufficient to reveal the existence of the state, does not account for the resonance being in close vicinity of the D^*^D threshold. To assess the fundamental properties of resonances that are close to thresholds, advanced parametrisations ought to be used^80–90^. A unitarised Breit–Wigner profile ${{\mathfrak{F}}}^{{{{{{\rm{U}}}}}}}$, described in Methods Eq. (47), is used in this analysis. The function ${{\mathfrak{F}}}^{{{{{{\rm{U}}}}}}}$ is built under two main assumptions.

#### Assumption 1

The newly observed state has quantum numbers J^P^ = 1^+^ and isospin I = 0 in accordance with the theoretical expectation for the ${{{{{{\rm{T}}}}}}}_{{{{{{\rm{c}}}}}}{{{{{\rm{c}}}}}}}^{+}$ ground state.

#### Assumption 2

The ${{{{{{\rm{T}}}}}}}_{{{{{{\rm{c}}}}}}{{{{{\rm{c}}}}}}}^{+}$ state is strongly coupled to the D^*^D channel, which is motivated by the proximity of the ${{{{{{\rm{T}}}}}}}_{{{{{{\rm{c}}}}}}{{{{{\rm{c}}}}}}}^{+}$ mass to the D^*^D mass threshold.

The derivation of the ${{\mathfrak{F}}}^{{{{{{\rm{U}}}}}}}$ profile relies on the assumed isospin symmetry for the ${{{{{{\rm{T}}}}}}}_{{{{{{\rm{c}}}}}}{{{{{\rm{c}}}}}}}^{+}\,\to {{{{{{\rm{D}}}}}}}^{* }{{{{{\rm{D}}}}}}$ decays and the coupled-channel interaction of the D^*+^D^0^ and D^*0^D^0^ system as required by unitarity and causality following Ref. ^91^. The resulting energy-dependent width of the ${{{{{{\rm{T}}}}}}}_{{{{{{\rm{c}}}}}}{{{{{\rm{c}}}}}}}^{+}$ state accounts explicitly for the ${{{{{{\rm{T}}}}}}}_{{{{{{\rm{c}}}}}}{{{{{\rm{c}}}}}}}^{+}\,\to {{{{{{\rm{D}}}}}}}^{0}{{{{{{\rm{D}}}}}}}^{0}{\pi }^{+}$, ${{{{{{\rm{T}}}}}}}_{{{{{{\rm{c}}}}}}{{{{{\rm{c}}}}}}}^{+}\,\to {{{{{{\rm{D}}}}}}}^{0}{{{{{{\rm{D}}}}}}}^{+}{\pi }^{0}$ and ${{{{{{\rm{T}}}}}}}_{{{{{{\rm{c}}}}}}{{{{{\rm{c}}}}}}}^{+}\,\to {{{{{{\rm{D}}}}}}}^{0}{{{{{{\rm{D}}}}}}}^{+}\gamma$ decays. The modification of the D^*^ meson lineshape^92^ due to contributions from triangle diagrams^93^ to the final-state interactions is neglected. Similarly to the ${{\mathfrak{F}}}^{{{{{{\rm{BW}}}}}}}$ profile, the ${{\mathfrak{F}}}^{{{{{{\rm{U}}}}}}}$ function has two parameters: the peak location *m*_U_, defined as the mass value where the real part of the complex amplitude vanishes, and the absolute value of the coupling constant *g* for the ${{{{{{\rm{T}}}}}}}_{{{{{{\rm{c}}}}}}{{{{{\rm{c}}}}}}}^{+}\,\to {{{{{{\rm{D}}}}}}}^{* }{{{{{\rm{D}}}}}}$ decay.

The detector mass resolution, ${\mathfrak{R}}$, is modelled with the sum of two Gaussian functions with a common mean, and parameters taken from simulation, see Methods. The widths of the Gaussian functions are corrected by a factor of 1.05, which accounts for a small residual difference between simulation and data^94–96^. The root mean square (RMS) of the resolution function is around 400 keV/*c*^2^.

A study of the D^0^*π*^+^ mass distribution for selected D^0^D^0^*π*^+^ combinations in the region above the D^*0^D^+^ mass threshold and below 3.9 GeV/*c*^2^ shows that approximately 90% of all D^0^D^0^*π*^+^ combinations contain a true D^*+^ meson. Therefore, the background component is parameterised with a product of the two-body phase-space function ${{{\Phi }}}_{{{{{{{\rm{D}}}}}}}^{* +}{{{{{{\rm{D}}}}}}}^{0}}$^97^ and a positive polynomial function *P*_*n*_, convolved with the detector resolution function ${\mathfrak{R}}$

2 $${B}_{n}=\left({{{\Phi }}}_{{{{{{{\rm{D}}}}}}}^{* +}{{{{{{\rm{D}}}}}}}^{0}}\times {P}_{n}\right)* {\mathfrak{R}},$$

where *n* denotes the order of the polynomial function, *n* = 2 is used in the default fit.

The D^0^D^0^*π*^+^ mass spectrum with non-D^0^ background subtracted is shown in Fig. 1 with the result of the fit using a model based on the ${{\mathfrak{F}}}^{{{{{{\rm{U}}}}}}}$ signal profile overlaid. The fit gives a signal yield of 186 ± 24 and a mass parameter relative to the D^*+^D^0^ mass threshold, *δm*_U_ of −359 ± 40 keV/*c*^2^. The statistical significances of the observed ${{{{{{\rm{T}}}}}}}_{{{{{{\rm{c}}}}}}{{{{{\rm{c}}}}}}}^{+}\,\to {{{{{{\rm{D}}}}}}}^{0}{{{{{{\rm{D}}}}}}}^{0}{\pi }^{+}$ signal and for the *δm*_U_ < 0 hypothesis are determined using Wilks’ theorem to be 22 and 9 standard deviations, respectively.

**Fig. 2:**
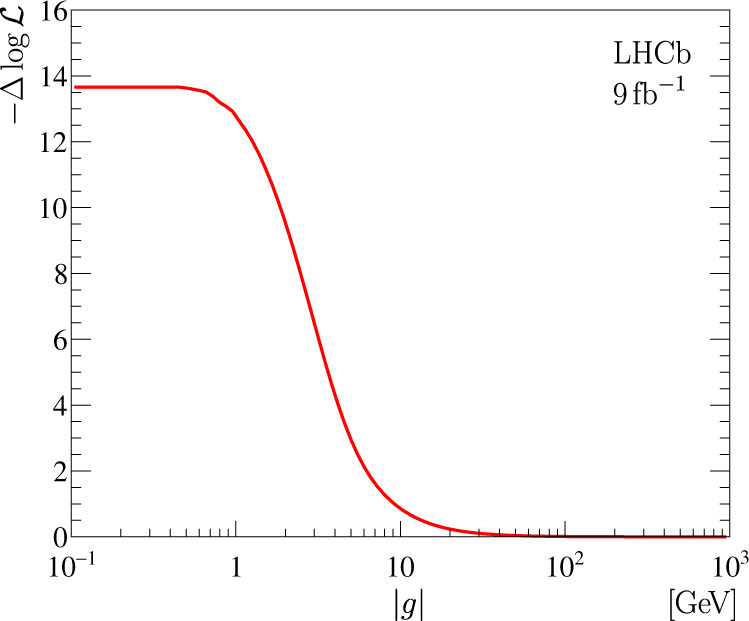
Likelihood profile for the $\left|g\right|$ parameter. Likelihood profile for the absolute value of the coupling constant *g* from the fit to the background-subtracted D^0^D^0^*π*^+^ mass spectrum with a model based on the ${{\mathfrak{F}}}^{{{{{{\rm{U}}}}}}}$ signal profile.

The width of the resonance is determined by the coupling constant *g* for small values of $\left|g\right|$. With increasing $\left|g\right|$, the width increases to an asymptotic value determined by the width of the D^*+^ meson, see Methods and Supplementary Fig. 7. In this regime of large $\left|g\right|$, the ${{\mathfrak{F}}}^{{{{{{\rm{U}}}}}}}$ signal profile exhibits a scaling property similar to the Flatté function^94,98,99^. The parameter $\left|g\right|$ effectively decouples from the fit model, and the model resembles the scattering-length approximation^81^. The likelihood profile for the parameter $\left|g\right|$ is shown in Fig. 2, where one can see a plateau at large values. At small values of the $\left|g\right|$ parameter, $\left|g\right|\, < \, 1\,{{{{{\rm{GeV}}}}}}$, the likelihood function is independent of $\left|g\right|$ because the resonance is too narrow for the details of the ${{\mathfrak{F}}}^{{{{{{\rm{U}}}}}}}$ signal profile to be resolved by the detector. The lower limits on the $\left|g\right|$ parameter of $\left|g\right| \, > \, 7.7\,(6.2)\,{{{{{\rm{GeV}}}}}}$ at 90% (95%) confidence level (CL) are obtained as the values where the difference in the negative log-likelihood $-{{\Delta }}\log {{{{{\mathcal{L}}}}}}$ is equal to 1.35 and 1.92, respectively. Smaller values for $\left|g\right|$ are further used for systematic uncertainty evaluation.

The mode relative to the D^*+^D^0^ mass threshold, $\delta {\mathfrak{m}}$, and the full width at half maximum (FWHM), ${\mathfrak{w}}$, for the ${{\mathfrak{F}}}^{{{{{{\rm{U}}}}}}}$ profile are found to be $\delta {\mathfrak{m}}=-361\pm 40 \,{{{{{\rm{keV}}}}}}/{c}^{2}$ and ${\mathfrak{w}}=47.8\pm 1.9{{{{{\rm{keV}}}}}}/{c}^{2}$, to be compared with those quantities determined for the ${{\mathfrak{F}}}^{{{{{{\rm{BW}}}}}}}$ signal profile of $\delta {\mathfrak{m}}=-279\pm 59 \,{{{{{\rm{keV}}}}}}/{c}^{2}$ and ${\mathfrak{w}}=409\pm 163 \,{{{{{\rm{keV}}}}}}/{c}^{2}$. They appear to be rather different. Nonetheless, both functions properly describe the data given the limited sample size, and accounting for the detector resolution, and residual background. To quantify the impact of these experimental effects, two ensembles of pseudoexperiments are performed. Firstly, pseudodata samples are generated with a model based on the ${{\mathfrak{F}}}^{{{{{{\rm{U}}}}}}}$ profile. The parameters used here are obtained from the default fit, and the size of the sample corresponds to the size of data sample. Each pseudodata sample is then analysed with a model based on the ${{\mathfrak{F}}}^{{{{{{\rm{BW}}}}}}}$ function. The obtained mean and RMS values for the parameters *δm*_BW_ and Γ_BW_ over the ensemble are shown in Table 2. The mass parameter *δm*_BW_ agrees well with the value determined from data^71^. The difference for the parameter Γ_BW_ does not exceed one standard deviation. Secondly, an ensemble of pseudodata samples generated with a model based on the ${{\mathfrak{F}}}^{{{{{{\rm{BW}}}}}}}$ profile is analysed with a model based on the ${{\mathfrak{F}}}^{{{{{{\rm{U}}}}}}}$ function. The obtained mean and RMS values for the *δm*_U_ parameter over an ensemble are also reported in Table 2. These values agree well with the result of the default fit to data. The results of these pseudoexperiments explain the seeming inconsistency between the models and illustrate the importance of an accurate description of the detector resolution and residual background given the limited sample size.

**Table 2:** Mean and root mean square (RMS) values for the *δm*_BW_, Γ_BW_ and *δm*_U_ parameters obtained from pseudoexperiments produced as a consistency check.

		Pseudoexperiments		
Parameter		Mean	RMS	Data
*δm*_BW_	$\left[\,{{{{{\rm{keV}}}}}}/{c}^{2}\right]$	−301	50	−273 ± 61^71^
Γ_BW_	$\left[\,{{{{{\rm{keV}}}}}}\right]$	222	121	410 ± 165^71^
*δm*_U_	$\left[\,{{{{{\rm{keV}}}}}}/{c}^{2}\right]$	−378	46	−359 ± 40

### Systematic uncertainties

Systematic uncertainties for the *δm*_U_ parameter are summarised in Table 3 and described in greater detail below. The systematic uncertainty related to the fit model is studied using pseudoexperiments with a set of alternative parameterisations. For each alternative model, an ensemble of pseudoexperiments is performed with parameters obtained from a fit to data. A fit with the baseline model is performed on each pseudoexperiment, and the mean values of the parameters of interest are evaluated over the ensemble. The absolute values of the differences between these mean values and the corresponding parameter values obtained from the fit to data are used to assess the systematic uncertainty due to the choice of the fit model. The maximal value of such differences over the considered set of alternative models is taken as the corresponding systematic uncertainty. The following sources of systematic uncertainty related to the fit model are considered:

- Imperfect knowledge of the detector resolution model. To estimate the associated systematic uncertainty a set of alternative resolution functions is tested: a symmetric variant of an Apollonios function^100^, a modified Gaussian function with symmetric power-law tails on both sides of the distribution^101,102^, a generalised symmetric Student’s *t*-distribution^103,104^, a symmetric Johnson’s S_U_ distribution^105,106^, and a modified Novosibirsk function^107^.
- A small difference in the detector resolution between data and simulation. A correction factor of 1.05 is applied to account for known discrepancies in modelling the detector resolution in simulation. This factor was studied for different decays^94–96,108–110^ and found to lie between 1.0 and 1.1. For decays with relatively low momentum tracks, this factor is close to 1.05, which is the nominal value used in this analysis. This factor is also cross-checked using large samples of D^*+^ → D^0^*π*^+^ decays, where a value of 1.06 is obtained. To assess the systematic uncertainty related to this factor, detector resolution models with correction factors of 1.0 and 1.1 are studied as alternatives.
- Parameterisation of the background component. To assess the associated systematic uncertainty, the order of the positive polynomial function of Eq. (2) is varied. In addition, to estimate a possible effect from a small contribution from three-body D^0^D^0^*π*^+^ combinations without an intermediate D^*+^ meson, a more general family of background models is tested
  3 $${B}_{nm}^{\prime}={B}_{n}+{{{\Phi }}}_{{{{{{{\rm{D}}}}}}}^{0}{{{{{{\rm{D}}}}}}}^{0}{\pi }^{+}}\times {P}_{m},$$
  where ${{{\Phi }}}_{{{{{{{\rm{D}}}}}}}^{0}{{{{{{\rm{D}}}}}}}^{0}{\pi }^{+}}$ denotes the three-body phase-space function^111^. The functions *B*_0_, *B*_1_, *B*_3_ and ${B}_{nm}^{\prime}$ with *n* ≤ 2, *m* ≤ 1 are used as alternative models for the estimation of the systematic uncertainty.
- Values of the coupling constants for the D^*^ → D*π* and D^*^ → D*γ* decays affecting the shape of the ${{\mathfrak{F}}}^{{{{{{\rm{U}}}}}}}$ signal profile. These coupling constants are calculated from the known branching fractions of the D^*^ → D*π* and D^*^ → D*γ* decays^10^, the measured natural width of the D^*+^ meson^10,112^ and the derived value for the natural width of the D^*0^ meson^66,81,113^. To assess the associated systematic uncertainty, a set of alternative models built around the ${{\mathfrak{F}}}^{{{{{{\rm{U}}}}}}}$ profiles, obtained with coupling constants varying within their calculated uncertainties, is studied.
- Unknown value of the $\left|g\right|$ parameter. In the baseline fit the value of the $\left|g\right|$ parameter is fixed to a large value. To assess the effect of this constraint the fit is repeated using the value of $\left|g\right|=8.08\,{{{{{\rm{GeV}}}}}}$, that corresponds to $-2{{\Delta }}\log {{{{{\mathcal{L}}}}}}=1$ for the most conservative likelihood profile for $\left|g\right|$ that accounts for the systematic uncertainty. The change of 7 keV/*c*^2^ of the *δm*_U_ parameter is assigned as the systematic uncertainty.

**Table 3:** Systematic uncertainties for the *δm*_U_ parameter.

Source	${\sigma }_{\delta {m}_{{{{{{\rm{U}}}}}}}}\left[\,{{{{{\rm{keV}}}}}}/{c}^{2}\right]$
Fit model	
Resolution model	2
Resolution correction factor	2
Background model	2
Coupling constants	1
Unknown value of $\left|g\right|$	${}_{-\,0}^{+\,7}$
Momentum scaling	3
Energy loss	1
D^*+^ − D^0^ mass difference	2
Total	${}_{-\,6}^{+\,9}$

The total uncertainty is calculated as the sum in quadrature of all components.

The calibration of the momentum scale of the tracking system is based upon large samples of B^+^ → J/*ψ*K^+^ and J/*ψ* → *μ*^+^*μ*^−^ decays^114^. The accuracy of the procedure has been checked using fully reconstructed B decays together with two-body ϒ(nS) and ${{{{{{\rm{K}}}}}}}_{{{{{{\rm{S}}}}}}}^{0}$ decays and the largest deviation of the bias in the momentum scale of *δα* = 3 × 10^−4^ is taken as the uncertainty^115^. This uncertainty is propagated for the parameters of interest using simulated samples, with momentum scale corrections of $\left(1\pm \delta \alpha \right)$ applied. Half of the difference between the obtained peak locations is taken as an estimate of the systematic uncertainty.

In the reconstruction the momenta of the charged tracks are corrected for energy loss in the detector material using the Bethe–Bloch formula^116,117^. The amount of the material traversed in the tracking system by a charged particle is known to have 10% accuracy^118^. To assess the corresponding uncertainty the magnitude of the calculated corrections is varied by ±10%. Half of the difference between the obtained peak locations is taken as an estimate of the systematic uncertainty due to energy loss corrections.

The mass of D^0^D^0^*π*^+^ combinations is calculated with the mass of each D^0^ meson constrained to the known value of the D^0^ mass^10^. This procedure produces negligible uncertainties for the *δm*_U_ parameter due to imprecise knowledge of the D^0^ mass. However, the small uncertainty of 2 keV/*c*^2^ for the known D^*+^ − D^0^ mass difference^10,112,119^ directly affects the values of these parameters and is assigned as a corresponding systematic uncertainty.

For the lower limit on the parameter $\left|g\right|$, only systematic uncertainties related to the fit model are considered. For each alternative model the likelihood profile curves are built and corresponding 90 and 95% CL lower limits are calculated using the procedure described above. The smallest of the resulting values is taken as the lower limit that accounts for the systematic uncertainty: $\left|g\right| \, > \, 5.1\,(4.3)\,{{{{{\rm{GeV}}}}}}$ at 90 (95%) CL.

### Results

**Fig. 3:**
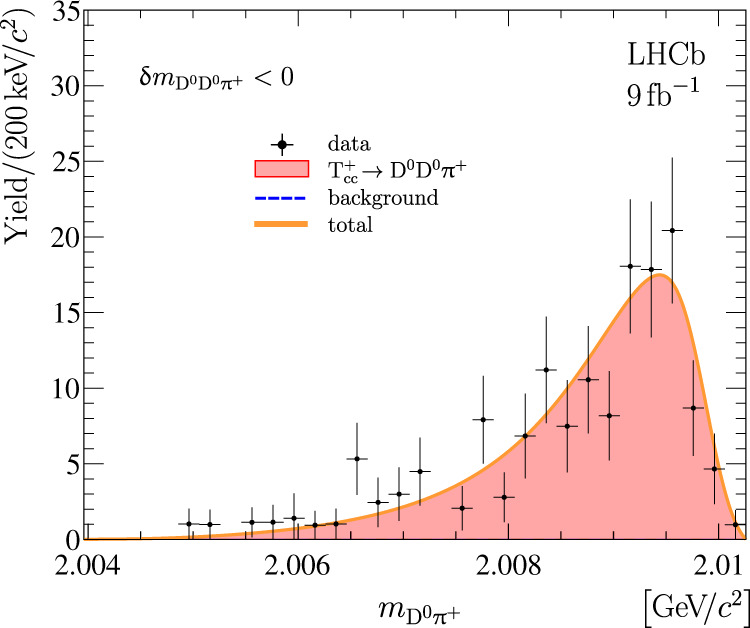
Mass distribution for D^0^*π*^+^ pairs. Mass distribution for D^0^*π*^+^ pairs from selected D^0^D^0^*π*^+^ candidates with a mass below the D^*+^D^0^ mass threshold with non-D^0^ background subtracted. The overlaid fit result is described in the text. The background component vanishes in the fit. Uncertainties on the data points are statistical only and represent one standard deviation, calculated as a sum in quadrature of the assigned weights from the background-subtraction procedure.

Studying the D^0^*π*^+^ mass distribution for ${{{{{{\rm{T}}}}}}}_{{{{{{\rm{c}}}}}}{{{{{\rm{c}}}}}}}^{+}\,\to {{{{{{\rm{D}}}}}}}^{0}{{{{{{\rm{D}}}}}}}^{0}{\pi }^{+}$ decays allows testing the hypothesis that the ${{{{{{\rm{T}}}}}}}_{{{{{{\rm{c}}}}}}{{{{{\rm{c}}}}}}}^{+}\,\to {{{{{{\rm{D}}}}}}}^{0}{{{{{{\rm{D}}}}}}}^{0}{\pi }^{+}$ decay proceeds through an intermediate off-shell D^*+^ meson. The background-subtracted D^0^*π*^+^ mass distribution for selected D^0^D^0^*π*^+^ candidates with the D^0^D^0^*π*^+^ mass with respect to the D^*+^D^0^ mass threshold, $\delta {m}_{{{{{{{\rm{D}}}}}}}^{0}{{{{{{\rm{D}}}}}}}^{0}{\pi }^{+}}$, below zero is shown in Fig. 3. Both D^0^*π*^+^ combinations are included in this plot. The two-dimensional distribution of the mass of one D^0^*π*^+^ combination versus the mass of another D^0^*π*^+^ combination is presented in Supplementary Fig. 10.

A fit is performed to this distribution with a model containing signal and background components. The signal component is derived from the ${{{{{{\mathcal{A}}}}}}}_{{{{{{\rm{U}}}}}}}$ amplitude, see Methods Eq. (48), and is convolved with a detector resolution for the D^0^*π*^+^ mass. This detector resolution function is modelled with a modified Gaussian function with power-law tails on both sides of the distribution^101,102^ and parameters taken from simulation. Similarly to the correction used for the D^0^D^0^*π*^+^ mass resolution function ${\mathfrak{R}}$, the width of the Gaussian function is corrected by a factor of 1.06 which is determined by studying large samples of D^*+^ → D^0^*π*^+^ decays. The RMS of the resolution function is around 220 keV/*c*^2^. The shape of the background component is derived from data for $\delta {m}_{{{{{{{\rm{D}}}}}}}^{0}{{{{{{\rm{D}}}}}}}^{0}{\pi }^{+}} > 0.6\,{{{{{\rm{MeV}}}}}}/{c}^{2}$. The fit results are overlaid in Fig. 3. The background component vanishes in the fit, and the D^0^*π*^+^ spectrum is consistent with the hypothesis that the ${{{{{{\rm{T}}}}}}}_{{{{{{\rm{c}}}}}}{{{{{\rm{c}}}}}}}^{+}\,\to {{{{{{\rm{D}}}}}}}^{0}{{{{{{\rm{D}}}}}}}^{0}{\pi }^{+}$ decay proceeds through an intermediate off-shell D^*+^ meson. This in turn favours the 1^+^ assignment for the spin-parity of the state.

**Fig. 4:**
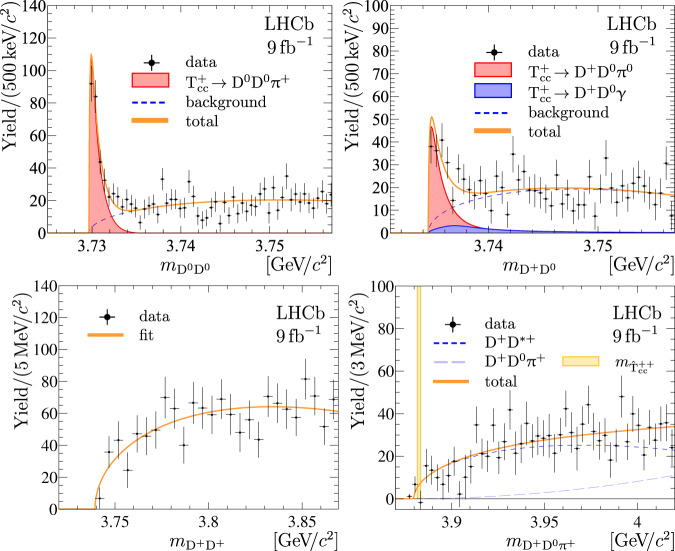
Mass distributions for selected D^0^D^0^, D^+^D^0^, D^+^D^+^ and D^+^D^0^*π*^+^ combinations. (Top) DD and DD*π*^+^ mass distributions for selected (left) D^0^D^0^ and (right) D^+^D^0^ candidates with the non-D background subtracted. The overlaid fit results are described in the text. For visibility the${{{{{{\rm{T}}}}}}}_{{{{{{\rm{c}}}}}}{{{{{\rm{c}}}}}}}^{+}\,\to {{{{{{\rm{D}}}}}}}^{+}{{{{{{\rm{D}}}}}}}^{0}{\pi }^{0}$ is stacked on top of the ${{{{{{\rm{T}}}}}}}_{{{{{{\rm{c}}}}}}{{{{{\rm{c}}}}}}}^{+}\,\to {{{{{{\rm{D}}}}}}}^{+}{{{{{{\rm{D}}}}}}}^{0}\gamma$ component. (Bottom) Mass distributions for selected (left) D^+^D^+^ and (right) D^+^D^0^*π*^+^ candidates with the non-D background subtracted. The vertical coloured band indicates the expected mass for the hypothetical ${\hat{{{{{{\rm{T}}}}}}}}_{{{{{{\rm{c}}}}}}{{{{{\rm{c}}}}}}}^{++}$ state. The overlaid fit results with background-only functions are described in the text. Uncertainties on the data points are statistical only and represent one standard deviation, calculated as a sum in quadrature of the assigned weights from the background-subtraction procedure.

Due to the proximity of the observed ${{{{{{\rm{T}}}}}}}_{{{{{{\rm{c}}}}}}{{{{{\rm{c}}}}}}}^{+}$ signal to the D^*+^D^0^ mass threshold, and the small energy release in the D^*+^ → D^0^*π*^+^ decay, the D^0^D^0^ mass distribution from the${{{{{{\rm{T}}}}}}}_{{{{{{\rm{c}}}}}}{{{{{\rm{c}}}}}}}^{+}\,\to {{{{{{\rm{D}}}}}}}^{0}{{{{{{\rm{D}}}}}}}^{0}{\pi }^{+}$ decay forms a narrow peak just above the D^0^D^0^ mass threshold. In a similar way, a peaking structure in the D^+^D^0^ mass spectrum just above the D^+^D^0^ mass threshold is expected from ${{{{{{\rm{T}}}}}}}_{{{{{{\rm{c}}}}}}{{{{{\rm{c}}}}}}}^{+}\,\to {{{{{{\rm{D}}}}}}}^{+}{{{{{{\rm{D}}}}}}}^{0}{\pi }^{0}$ and ${{{{{{\rm{T}}}}}}}_{{{{{{\rm{c}}}}}}{{{{{\rm{c}}}}}}}^{+}\,\to {{{{{{\rm{D}}}}}}}^{+}{{{{{{\rm{D}}}}}}}^{0}\gamma$ decays, both proceeding via off-shell intermediate D^*+^D^0^ and D^*0^D^+^ states. The D^0^D^0^ and D^+^D^0^ final states are reconstructed and selected similarly to the D^0^D^0^*π*^+^ final state, where the D^+^ → K^−^*π*^+^*π*^−^ decay channel is used. The background-subtracted D^0^D^0^ and D^+^D^0^ mass distributions are shown in Fig. 4 (top), where narrow structures are clearly visible just above the DD thresholds. Fits to these distributions are performed using models consisting of two components: a signal component *F*_DD_ described in Methods Eqs. (70) and (71) and obtained via integration of the matrix elements for the ${{{{{{\rm{T}}}}}}}_{{{{{{\rm{c}}}}}}{{{{{\rm{c}}}}}}}^{+}\,\to {{{{{\rm{D}}}}}}{{{{{\rm{D}}}}}}\pi /\gamma$ decays with the ${{\mathfrak{F}}}^{{{{{{\rm{U}}}}}}}$ profile, and a background component, parameterised as a product of the two-body phase-space function Φ_DD_ and a positive linear function *P*_1_. The fit results are overlaid in Fig. 4 (top). The signal yields in the D^0^D^0^ and D^+^D^0^ spectra are found to be 263 ± 23 and 171 ± 26, respectively. The statistical significance of the observed ${{{{{{\rm{T}}}}}}}_{{{{{{\rm{c}}}}}}{{{{{\rm{c}}}}}}}^{+}\,\to {{{{{{\rm{D}}}}}}}^{0}{{{{{{\rm{D}}}}}}}^{0}{{{{{\rm{X}}}}}}$ and ${{{{{{\rm{T}}}}}}}_{{{{{{\rm{c}}}}}}{{{{{\rm{c}}}}}}}^{+}\,\to {{{{{{\rm{D}}}}}}}^{+}{{{{{{\rm{D}}}}}}}^{0}{{{{{\rm{X}}}}}}$ signals, where X stands for non-reconstructed pions or photons, is estimated using Wilks’ theorem^120^ and is found to be in excess of 20 and 10 standard deviations, respectively. The relative yields for the signals observed in the D^0^D^0^*π*^+^, D^0^D^0^ and D^0^D^+^ mass spectra agree with the expectations of the model described in Methods where the decay of an isoscalar ${{{{{{\rm{T}}}}}}}_{{{{{{\rm{c}}}}}}{{{{{\rm{c}}}}}}}^{+}$ state via the D^*^D channel with an intermediate off-shell D^*^ meson is assumed.

The observation of the near-threshold signals in the D^0^D^0^ and D^+^D^0^ mass spectra, along with the signal shapes and yields, all agree with the isoscalar ${{{{{{\rm{T}}}}}}}_{{{{{{\rm{c}}}}}}{{{{{\rm{c}}}}}}}^{+}$ hypothesis for the narrow signal observed in the D^0^D^0^*π*^+^ mass spectrum. However, an alternative interpretation could be that this state is the I_3_ = 0 component of a ${\hat{{{{{{\rm{T}}}}}}}}_{{{{{{\rm{c}}}}}}{{{{{\rm{c}}}}}}}$ isotriplet (${\hat{{{{{{\rm{T}}}}}}}}_{{{{{{\rm{c}}}}}}{{{{{\rm{c}}}}}}}^{0}$,${\hat{{{{{{\rm{T}}}}}}}}_{{{{{{\rm{c}}}}}}{{{{{\rm{c}}}}}}}^{+}$,${\hat{{{{{{\rm{T}}}}}}}}_{{{{{{\rm{c}}}}}}{{{{{\rm{c}}}}}}}^{++}$) with ${{{{{\rm{c}}}}}}{{{{{\rm{c}}}}}}\overline{{{{{{\rm{u}}}}}}}\overline{{{{{{\rm{u}}}}}}}$, ${{{{{\rm{c}}}}}}{{{{{\rm{c}}}}}}\overline{{{{{{\rm{u}}}}}}}\overline{{{{{{\rm{d}}}}}}}$ and ${{{{{\rm{c}}}}}}{{{{{\rm{c}}}}}}\overline{{{{{{\rm{d}}}}}}}\overline{{{{{{\rm{d}}}}}}}$ quark content, respectively. Assuming that the observed peak corresponds to the ${\hat{{{{{{\rm{T}}}}}}}}_{{{{{{\rm{c}}}}}}{{{{{\rm{c}}}}}}}^{+}$ component and using the estimates for the ${\hat{{{{{{\rm{T}}}}}}}}_{{{{{{\rm{c}}}}}}{{{{{\rm{c}}}}}}}$ mass splitting from Methods Eqs. (85) and (86), the masses of the ${\hat{{{{{{\rm{T}}}}}}}}_{{{{{{\rm{c}}}}}}{{{{{\rm{c}}}}}}}^{0}$ and ${\hat{{{{{{\rm{T}}}}}}}}_{{{{{{\rm{c}}}}}}{{{{{\rm{c}}}}}}}^{++}$ states are estimated to be slightly below the D^0^D^*0^ and slightly above the D^+^D^*+^ mass thresholds, respectively:

4 $${m}_{{\hat{{{{{{\rm{T}}}}}}}}_{{{{{{\rm{c}}}}}}{{{{{\rm{c}}}}}}}^{0}}-\left({m}_{{{{{{{\rm{D}}}}}}}^{0}}+{m}_{{{{{{{\rm{D}}}}}}}^{* 0}}\right)=-2.8\pm 1.5\,{{{{{\rm{MeV}}}}}}/{c}^{2},$$

5 $${m}_{{\hat{{{{{{\rm{T}}}}}}}}_{{{{{{\rm{c}}}}}}{{{{{\rm{c}}}}}}}^{++}}-\left({m}_{{{{{{{\rm{D}}}}}}}^{+}}+{m}_{{{{{{{\rm{D}}}}}}}^{* +}}\right)=-2.7\pm 1.3\,{{{{{\rm{MeV}}}}}}/{c}^{2}.$$

With these mass assignments, assuming equal production of all three ${\hat{{{{{{\rm{T}}}}}}}}_{{{{{{\rm{c}}}}}}{{{{{\rm{c}}}}}}}$ components, the ${\hat{{{{{{\rm{T}}}}}}}}_{{{{{{\rm{c}}}}}}{{{{{\rm{c}}}}}}}^{0}$ state would be an extra narrow state that decays into the D^0^D^0^*π*^0^ and D^0^D^0^*γ* final states via an off-shell D^*0^ meson. These decays would contribute to the narrow near-threshold enhancement in the D^0^D^0^ spectrum, and increase the signal in the D^0^D^0^ mass spectrum by almost a factor of three. The ${\hat{{{{{{\rm{T}}}}}}}}_{{{{{{\rm{c}}}}}}{{{{{\rm{c}}}}}}}^{++}$ state would decay via an on-shell D^*+^ meson ${\hat{{{{{{\rm{T}}}}}}}}_{{{{{{\rm{c}}}}}}{{{{{\rm{c}}}}}}}^{++}\,\to {{{{{{\rm{D}}}}}}}^{+}{{{{{{\rm{D}}}}}}}^{* +}$; therefore, it could be a relatively wide state, with a width up to a few Me^121^. Therefore, it would manifest itself as a peak with a moderate width in the D^+^D^0^*π*^+^ mass spectrum with a yield comparable to that of the ${\hat{{{{{{\rm{T}}}}}}}}_{{{{{{\rm{c}}}}}}{{{{{\rm{c}}}}}}}^{+}\,\to {{{{{{\rm{D}}}}}}}^{0}{{{{{{\rm{D}}}}}}}^{0}{\pi }^{+}$ decays. In addition, it would contribute to the D^+^D^0^ mass spectrum, tripling the contribution from the ${\hat{{{{{{\rm{T}}}}}}}}_{{{{{{\rm{c}}}}}}{{{{{\rm{c}}}}}}}^{+}$ decays. However, due to the larger mass of the ${\hat{{{{{{\rm{T}}}}}}}}_{{{{{{\rm{c}}}}}}{{{{{\rm{c}}}}}}}^{++}$ state and its larger width, this contribution should be wider, making it more difficult to disentangle from the background. Finally, the ${\hat{{{{{{\rm{T}}}}}}}}_{{{{{{\rm{c}}}}}}{{{{{\rm{c}}}}}}}^{++}$ state would make a contribution to the D^+^D^+^ spectrum with a yield similar to the contribution from ${\hat{{{{{{\rm{T}}}}}}}}_{{{{{{\rm{c}}}}}}{{{{{\rm{c}}}}}}}^{+}\,\to {{{{{{\rm{D}}}}}}}^{0}{{{{{{\rm{D}}}}}}}^{+}{\pi }^{0}/\gamma$ decays to the D^0^D^+^ spectrum, but wider. The mass spectra for D^+^D^+^ and D^+^D^0^*π*^+^ combinations are shown in Fig. 4 (bottom).

Neither distribution exhibits any narrow signal-like structure. Fits to these spectra are performed using the following background-only functions:

6 $${B}_{{{{{{{\rm{D}}}}}}}^{+}{{{{{{\rm{D}}}}}}}^{+}}={{{\Phi }}}_{{{{{{{\rm{D}}}}}}}^{+}{{{{{{\rm{D}}}}}}}^{+}}\times {P}_{1},$$

7 $${B}_{{{{{{{\rm{D}}}}}}}^{+}{{{{{{\rm{D}}}}}}}^{0}{\pi }^{+}}=\left({{{\Phi }}}_{{{{{{{\rm{D}}}}}}}^{+}{{{{{{\rm{D}}}}}}}^{* +}}\times {P}_{1}\right)* {\mathfrak{R}}+{{{\Phi }}}_{{{{{{{\rm{D}}}}}}}^{+}{{{{{{\rm{D}}}}}}}^{0}{\pi }^{+}}\times {P}_{0}.$$

The results of these fits are overlaid in Fig. 4 (bottom). The absence of any signals in the D^+^D^+^ and D^+^D^0^*π*^+^ mass spectra is therefore a strong argument in favour of the isoscalar nature of the observed peak in the D^0^D^0^*π*^+^ mass spectrum.

The interference between two virtual channels for the ${{{{{{\rm{T}}}}}}}_{{{{{{\rm{c}}}}}}{{{{{\rm{c}}}}}}}^{+}\,\to {{{{{{\rm{D}}}}}}}^{0}{{{{{{\rm{D}}}}}}}^{0}{\pi }^{+}$ decay, corresponding to two amplitude terms, see Methods Eq. (35), is studied by setting the term proportional to *C* in Methods Eq. (39) to be equal to zero. This causes a 43% reduction in the decay rate, pointing to a large interference. The same procedure applied to the ${{{{{{\rm{T}}}}}}}_{{{{{{\rm{c}}}}}}{{{{{\rm{c}}}}}}}^{+}\,\to {{{{{{\rm{D}}}}}}}^{+}{{{{{{\rm{D}}}}}}}^{0}{\pi }^{0}$ decays gives the contribution of 45% for the interference between the $\left({{{{{{\rm{D}}}}}}}^{* +}\,\to {{{{{{\rm{D}}}}}}}^{+}{\pi }^{0}\right){{{{{{\rm{D}}}}}}}^{0}$ and $\left({{{{{{\rm{D}}}}}}}^{* 0}\,\to {{{{{{\rm{D}}}}}}}^{0}{\pi }^{0}\right){{{{{{\rm{D}}}}}}}^{+}$ channels. For ${{{{{{\rm{T}}}}}}}_{{{{{{\rm{c}}}}}}{{{{{\rm{c}}}}}}}^{+}\,\to {{{{{{\rm{D}}}}}}}^{+}{{{{{{\rm{D}}}}}}}^{0}\gamma$ decays the role of the interference between the $\left({{{{{{\rm{D}}}}}}}^{* +}\,\to {{{{{{\rm{D}}}}}}}^{+}\gamma \right){{{{{{\rm{D}}}}}}}^{0}$ and $\left({{{{{{\rm{D}}}}}}}^{* 0}\,\to {{{{{{\rm{D}}}}}}}^{0}\gamma \right){{{{{{\rm{D}}}}}}}^{+}$ channels is estimated by equating to zero the ${{\mathfrak{F}}}_{+}{{\mathfrak{F}}}_{0}^{* }$ and ${{\mathfrak{F}}}_{+}^{* }{{\mathfrak{F}}}_{0}$ terms in Methods Eqs. (45) and (46). The interference contribution is found to be 33%.

Using the model described earlier and results of the fit to the D^0^D^0^*π*^+^ mass spectrum, the position of the amplitude pole $\hat{s}$ in the complex plane, responsible for the appearance of the narrow structure in the D^0^D^0^*π*^+^ mass spectrum is determined. The pole parameters, mass *m*_pole_ and width Γ_pole_, are defined through the pole location $\hat{s}$ as

8 $$\sqrt{\hat{s}}\equiv {m}_{{{{{{\rm{pole}}}}}}}-\frac{i}{2}{{{\Gamma }}}_{{{{{{\rm{pole}}}}}}}.$$

**Fig. 5:**
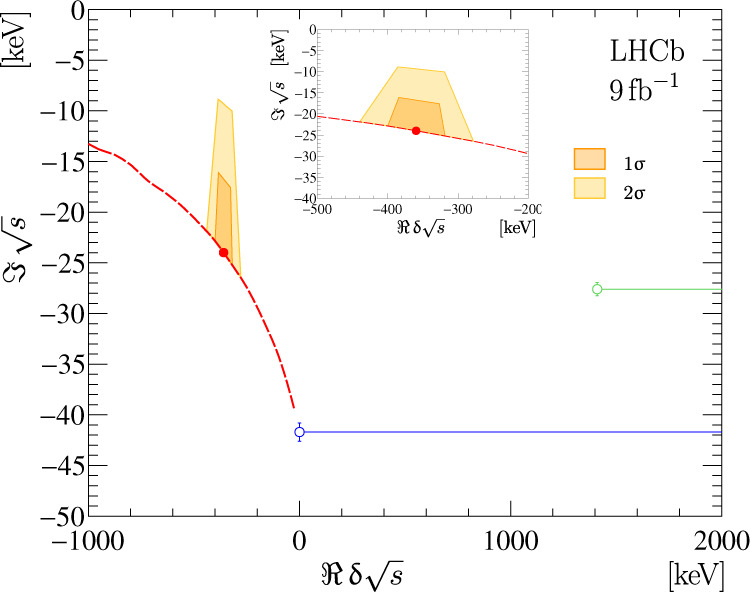
Complex plane of the $\delta \sqrt{s}$ variable. Complex plane of the $\delta \sqrt{s}$ variable. The dashed red line shows the allowed region for large $\left|g\right|$ values. The filled red circle indicates the best estimate for the pole location and the filled regions show 1*σ* and 2*σ* confidence regions. Open blue and green circles show the branch points corresponding to the D^*+^D^0^ and D^*0^D^+^ channels, respectively, and the corresponding blue and green lines indicate branch cuts. Three other branch points at $\sqrt{s}$ of ${m}_{{{{{{{\rm{D}}}}}}}^{0}}+{m}_{{{{{{{\rm{D}}}}}}}^{+}}$, ${m}_{{{{{{{\rm{D}}}}}}}^{0}}+{m}_{{{{{{{\rm{D}}}}}}}^{+}}+{m}_{{\pi }^{0}}$ and $2{m}_{{{{{{{\rm{D}}}}}}}^{0}}+{m}_{{\pi }^{+}}$, corresponding to the openings of the D^0^D^+^*γ*, D^0^D^+^*π*^0^ and D^0^D^0^*π*^+^ decay channels, are outside of the displayed region.

The pole location $\hat{s}$ is a solution to the equation

9 $$\begin{array}{r}\frac{1}{{{{{{{\mathcal{A}}}}}}}_{{{{{{\rm{U}}}}}}}^{II}(\hat{s})}=0,\end{array}$$

where ${{{{{{\mathcal{A}}}}}}}_{{{{{{\rm{U}}}}}}}^{II}(s)$ denotes the amplitude on the second Riemann sheet defined in Methods Eq. (64). For large coupling $\left|g\right|$ the position of the resonance pole is uniquely determined by the parameter *δm*_U_, i.e., the binding energy and the width of the D^*+^ meson. Figure 5 shows the complex plane of the $\delta \sqrt{s}$ variable, defined as

10 $$\delta \sqrt{s}\equiv \sqrt{s}-\left({m}_{{{{{{{\rm{D}}}}}}}^{* +}}+{m}_{{{{{{{\rm{D}}}}}}}^{0}}\right).$$

All possible positions of the pole for $\left|g\right|\gg {m}_{{{{{{{\rm{D}}}}}}}^{0}}+{m}_{{{{{{{\rm{D}}}}}}}^{* +}}$ are located on a red dashed curve in Fig. 5. The behaviour of the curve can be understood as follows: with an increase of the binding energy (distance to the D^*+^D^0^ mass threshold), the width gets narrower; and when the parameter *δm*_U_ approaches zero, the pole touches the D^0^D^*+^ cut and moves to the other complex sheet, i.e., the state becomes virtual. For smaller values of $\left|g\right|$, the pole is located between the limiting curve and the *ℜ* *s* = 0 line. The pole parameters are found to be

11 $$\delta {m}_{{{{{{\rm{pole}}}}}}}=-360\pm 4{0}_{-0}^{+4}\,{{{{{\rm{keV}}}}}}/{c}^{2},$$

12 $${{{\Gamma }}}_{{{{{{\rm{pole}}}}}}}=48\pm {2}_{-14}^{+0}\,{{{{{\rm{keV}}}}}},$$

where the first uncertainty is due to the *δm*_U_ parameter and the second is due to the unknown value of the $\left|g\right|$ parameter. The peak is well separated from the D^*+^D^0^ threshold in the D^0^D^0^*π*^+^ mass spectrum. Hence, as for an isolated narrow resonance, the parameters of the pole are similar to the visible peak parameters, namely the mode $\delta {\mathfrak{m}}$ and FWHM ${\mathfrak{w}}$.

The systematic uncertainties quoted here do not account for the possibility that any of the underlying assumptions on which the model is built are not valid. For example, as shown earlier the data are consistent with a wide range of FHWM ${\mathfrak{w}}$ values for the signal profile. Therefore the pole width Γ_pole_ is based mainly on the ${{{{{{\rm{T}}}}}}}_{{{{{{\rm{c}}}}}}{{{{{\rm{c}}}}}}}^{+}$ amplitude model and the value of the *m*_U_ parameter determined from the fit to the D^0^D^0^*π*^+^ mass spectrum.

A study of the behaviour of the ${{{{{{\mathcal{A}}}}}}}_{{{{{{\rm{U}}}}}}}(s)$ amplitude in the vicinity of the D^*+^D^0^ mass threshold leads to the determination of the low-energy scattering parameters, namely the scattering length, *a*, and the effective range, *r*. These parameters are defined via the coefficients of the first two terms of the Taylor expansion of the inverse non-relativistic amplitude^122^, i.e.,

13 $${{{{{{\mathcal{A}}}}}}}_{{{{{{\rm{NR}}}}}}}^{-1}=\frac{1}{a}+r\frac{{k}^{2}}{2}-ik+{{{{{\mathcal{O}}}}}}({k}^{4}),$$

where *k* is the wave number. For $\delta \sqrt{s}\, \lesssim -{{{\Gamma }}}_{{{{{{{\rm{D}}}}}}}^{* +}}$ the inverse amplitude from Eq. (47) matches Eq. (13) up to a scale parameter obtained numerically, see Methods Eq. (74). The value of the scattering length is found to be

14 $$a=\left[-\left(7.16\pm 0.51\right)+i\, \left(1.85\pm 0.28\right)\right]\,{{{{{\rm{fm}}}}}}.$$

Typically, a non-vanishing imaginary part of the scattering length indicates the presence of inelastic channels^123^; however, in this case the non-zero imaginary part is related to the lower threshold, ${{{{{{\rm{T}}}}}}}_{{{{{{\rm{c}}}}}}{{{{{\rm{c}}}}}}}^{+}\,\to {{{{{{\rm{D}}}}}}}^{0}{{{{{{\rm{D}}}}}}}^{0}{\pi }^{+}$, and is determined by the width of the D^*+^ meson. The real part of the scattering length *a* is negative indicating attraction. This can be interpreted as the characteristic size of the state^16^,

15 $${R}_{a}\equiv -\Re \,a=7.16\pm 0.51\,{{{{{\rm{fm}}}}}}.$$

For the ${{{{{{\mathcal{A}}}}}}}_{{{{{{\rm{U}}}}}}}$ amplitude the effective range *r* is non-positive and proportional to ${\left|g\right|}^{-2}$, see Methods Eq. (76). Its value is consistent with zero for the baseline fit. An upper limit on the −*r* value is set as

16 $$0\,\,\le \,\,-r\, < \, 11.9\,(16.9)\,{{{{{\rm{fm}}}}}}{{{{{\rm{at}}}}}}\,90(95) \% {{{{{\rm{CL}}}}}}.$$

The Weinberg compositeness criterion^124,125^ makes use of the relation between the scattering length and the effective range to construct the compositeness variable *Z*,

17 $$\begin{array}{r}Z=1-\sqrt{\frac{1}{1+2\left|r/\Re \,a\right|}},\end{array}$$

for which *Z* = 1 corresponds to a compact state that does not interact with the continuum, while *Z* = 0 indicates a composite state formed by compound interaction. Using the relation between *r* and ∣*g*∣ from Methods Eq. (76), one finds $Z\propto {\left|g\right|}^{-2}$ for large values of $\left|g\right|$. The default fit corresponds to large values of $\left|g\right|$, and thus, *Z* approaching to zero. A non-zero value of *Z* would require a smaller value of $\left|g\right|$, i.e., smaller resonance width, see Supplementary Fig. 7. The following upper limit of the compositeness parameter *Z* is set:

18 $$Z\, < \, 0.52\,(0.58)\,{{{{{\rm{at}}}}}}\,90\,(95) \% \,{{{{{\rm{CL}}}}}}.$$

Another estimate of the characteristic size is obtained from the value of the binding energy Δ*E*. Within the interpretation of the ${{{{{{\rm{T}}}}}}}_{{{{{{\rm{c}}}}}}{{{{{\rm{c}}}}}}}^{+}$ state as a bound D^*+^D^0^ molecular-like state, the binding energy is Δ*E* = − *δm*_U_. The characteristic momentum scale *γ*^16^ is estimated to be

19 $$\gamma =\sqrt{2\mu {{\Delta }}E}=26.4\pm 1.5\,{{{{{\rm{MeV}}}}}}/c,$$

where *μ* is the reduced mass of the D^*+^D^0^ system. This value of the momentum scale in turn corresponds to a characteristic size *R*_Δ*E*_ of the molecular-like state,

20 $${R}_{{{\Delta }}E}\equiv \frac{1}{\gamma }=7.5\pm 0.4\,{{{{{\rm{fm}}}}}},$$

which is consistent with the *R*_*a*_ estimate from the scattering length.

**Fig. 6:**
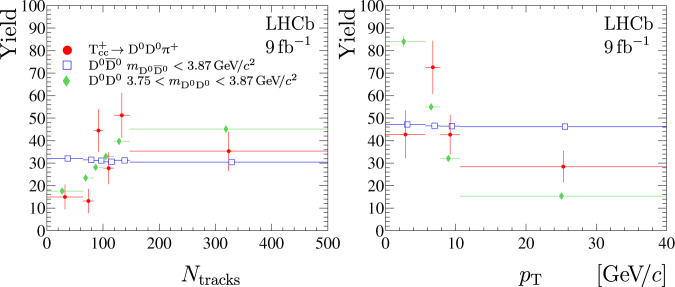
Track multiplicity and transverse momentum distributions. (Left) Background-subtracted distributions for the multiplicity of tracks reconstructed in the vertex detector for (red circles) ${{{{{{\rm{T}}}}}}}_{{{{{{\rm{c}}}}}}{{{{{\rm{c}}}}}}}^{+}\,\to {{{{{{\rm{D}}}}}}}^{0}{{{{{{\rm{D}}}}}}}^{0}{\pi }^{+}$ signal, low-mass (blue open squares) ${{{{{{\rm{D}}}}}}}^{0}{\overline{{{{{{\rm{D}}}}}}}}^{0}$ and (green filled diamonds) D^0^D^0^ pairs. The binning scheme is chosen to have an approximately uniform distribution for ${{{{{{\rm{D}}}}}}}^{0}{\overline{{{{{{\rm{D}}}}}}}}^{0}$ pairs. The distributions for the ${{{{{{\rm{D}}}}}}}^{0}{\overline{{{{{{\rm{D}}}}}}}}^{0}$ and D^0^D^0^ pairs are normalised to the same yields as the ${{{{{{\rm{T}}}}}}}_{{{{{{\rm{c}}}}}}{{{{{\rm{c}}}}}}}^{+}\,\to {{{{{{\rm{D}}}}}}}^{0}{{{{{{\rm{D}}}}}}}^{0}{\pi }^{+}$ signal. (right) Background-subtracted transverse momentum spectra for (red circles) ${{{{{{\rm{T}}}}}}}_{{{{{{\rm{c}}}}}}{{{{{\rm{c}}}}}}}^{+}\,\to {{{{{{\rm{D}}}}}}}^{0}{{{{{{\rm{D}}}}}}}^{0}{\pi }^{+}$ signal, (blue open squares) low-mass${{{{{{\rm{D}}}}}}}^{0}{\overline{{{{{{\rm{D}}}}}}}}^{0}$ and (green filled diamonds) D^0^D^0^ pairs. The binning scheme is chosen to have an approximately uniform distribution for ${{{{{{\rm{D}}}}}}}^{0}{\overline{{{{{{\rm{D}}}}}}}}^{0}$ pairs. The distributions for the ${{{{{{\rm{D}}}}}}}^{0}{\overline{{{{{{\rm{D}}}}}}}}^{0}$ and D^0^D^0^ pairs are normalised to the same yields as ${{{{{{\rm{T}}}}}}}_{{{{{{\rm{c}}}}}}{{{{{\rm{c}}}}}}}^{+}\,\to {{{{{{\rm{D}}}}}}}^{0}{{{{{{\rm{D}}}}}}}^{0}{\pi }^{+}$ signal. For better visualisation, the points are slightly displaced from the bin centres. For better visualisation, the points are slightly displaced from the bin centres. Uncertainties on the data points are statistical only and represent one standard deviation.

**Fig. 7:**
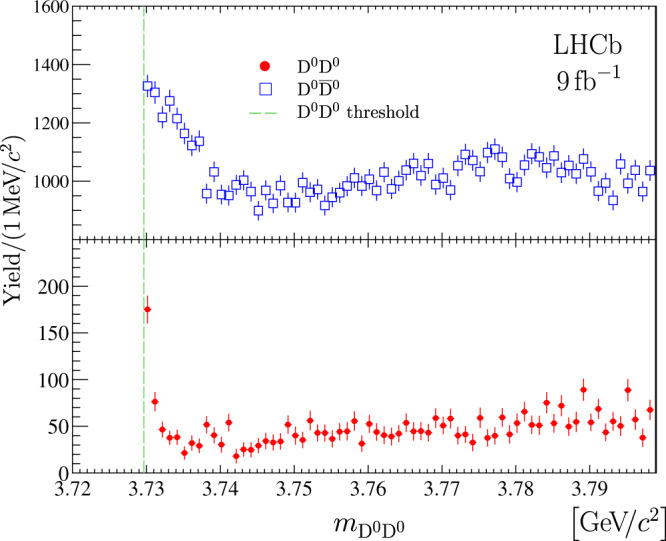
Mass distributions for D^0^D^0^ and ${{{{{{\rm{D}}}}}}}^{0}{\overline{{{{{{\rm{D}}}}}}}}^{0}$ candidates. Background-subtracted D^0^D^0^ and ${{{{{{\rm{D}}}}}}}^{0}{\overline{{{{{{\rm{D}}}}}}}}^{0}$ mass distributions. The near-threshold enhancement in the D^0^D^0^ channel corresponds to partially reconstructed ${{{{{{\rm{T}}}}}}}_{{{{{{\rm{c}}}}}}{{{{{\rm{c}}}}}}}^{+}\to {{{{{{\rm{D}}}}}}}^{0}{{{{{{\rm{D}}}}}}}^{0}{\pi }^{+}$ decays, while in the${{{{{{\rm{D}}}}}}}^{0}{\overline{{{{{{\rm{D}}}}}}}}^{0}$ channel the threshold enhancement corresponds to partially reconstructed ${\chi }_{{{{{{\rm{c}}}}}}1}(3872)\to {{{{{{\rm{D}}}}}}}^{0}{\overline{{{{{{\rm{D}}}}}}}}^{0}{\pi }^{0}$ decays. The ${{{{{{\rm{D}}}}}}}^{0}{\overline{{{{{{\rm{D}}}}}}}}^{0}$ mass distribution is zero-suppressed for better visualisation. Uncertainties on the data points are statistical only and represent one standard deviation, calculated as a sum in quadrature of the assigned weights from the background-subtraction procedure.

For high-energy hadroproduction of a state with such a large size, *R*_*a*_ or *R*_Δ*E*_, one expects a strong dependency of the production rate on event multiplicity, similar to that observed for the *χ*_c1_(3872) state^126^. The background-subtracted distribution of the number of tracks reconstructed in the vertex detector, *N*_tracks_, is shown in Fig. 6 (left) together with the distributions for low-mass ${{{{{{\rm{D}}}}}}}^{0}{\overline{{{{{{\rm{D}}}}}}}}^{0}$ pairs with ${m}_{{{{{{{\rm{D}}}}}}}^{0}{\overline{{{{{{\rm{D}}}}}}}}^{0}}\, < \, 3.87\,{{{{{\rm{GeV}}}}}}/{c}^{2}$ and low-mass D^0^D^0^ pairs with mass $3.75 < {m}_{{{{{{{\rm{D}}}}}}}^{0}{{{{{{\rm{D}}}}}}}^{0}}\, < \, 3.87\,{{{{{\rm{GeV}}}}}}/{c}^{2}$. The former is dominated by ${{{{{\rm{p}}}}}}{{{{{\rm{p}}}}}}\,\to {{{{{\rm{c}}}}}}{\overline{{{{{{\rm{c}}}}}}}}{{{{{\rm{X}}}}}}$ production, while the latter is presumably dominated by the double-parton scattering process^74,127^. The chosen interval for ${{{{{{\rm{D}}}}}}}^{0}{\overline{{{{{{\rm{D}}}}}}}}^{0}$ pairs includes the region populated by the ${\chi }_{{{{{{\rm{c}}}}}}1}(3872)\,\to {{{{{{\rm{D}}}}}}}^{0}{\overline{{{{{{\rm{D}}}}}}}}^{0}{\pi }^{0}/\gamma$ decays; however, this contribution is small, see Fig. 7. The *χ*_c1_(3872) production cross-section is suppressed with respect to the conventional charmonium state *ψ*(2S) at large track multiplicities^126^. It is noteworthy that the track multiplicity distribution for the ${{{{{{\rm{T}}}}}}}_{{{{{{\rm{c}}}}}}{{{{{\rm{c}}}}}}}^{+}$ state differs from that of the low-mass ${{{{{{\rm{D}}}}}}}^{0}{\overline{{{{{{\rm{D}}}}}}}}^{0}$ pairs, in particular, no suppression at large multiplicity is observed. A *p* value for the consistency of the track multiplicity distributions for ${{{{{{\rm{T}}}}}}}_{{{{{{\rm{c}}}}}}{{{{{\rm{c}}}}}}}^{+}$ production and low-mass ${{{{{{\rm{D}}}}}}}^{0}{\overline{{{{{{\rm{D}}}}}}}}^{0}$ pairs is found to be 0.1%. It is interesting to note that the multiplicity distribution for ${{{{{{\rm{T}}}}}}}_{{{{{{\rm{c}}}}}}{{{{{\rm{c}}}}}}}^{+}$ production and the one for D^0^D^0^-pairs with $3.75\, < \, {m}_{{{{{{{\rm{D}}}}}}}^{0}{{{{{{\rm{D}}}}}}}^{0}}\, < \, 3.87\,{{{{{\rm{GeV}}}}}}/{c}^{2}$ are consistent with a corresponding *p* value of 12%. The similarity between ${{{{{{\rm{T}}}}}}}_{{{{{{\rm{c}}}}}}{{{{{\rm{c}}}}}}}^{+}$ production, which is inherently a single parton scattering process, and the distribution for process dominated by a double-parton scattering is surprising.

The transverse momentum spectrum for the ${{{{{{\rm{T}}}}}}}_{{{{{{\rm{c}}}}}}{{{{{\rm{c}}}}}}}^{+}$ state is compared with those for the low-mass ${{{{{{\rm{D}}}}}}}^{0}{\overline{{{{{{\rm{D}}}}}}}}^{0}$ and D^0^D^0^ pairs in Fig. 6 (right). The *p* values for the consistency of the *p*_T_ spectra for the ${{{{{{\rm{T}}}}}}}_{{{{{{\rm{c}}}}}}{{{{{\rm{c}}}}}}}^{+}$ state and low-mass ${{{{{{\rm{D}}}}}}}^{0}{\overline{{{{{{\rm{D}}}}}}}}^{0}$ pairs are 1.4%, and 0.02% for low-mass D^0^D^0^ pairs. More data are needed for further conclusions.

The background-subtracted D^0^D^0^ mass distribution in a wider mass range is shown in Fig. 7 together with a similar distribution for ${{{{{{\rm{D}}}}}}}^{0}{\overline{{{{{{\rm{D}}}}}}}}^{0}$ pairs. In the D^0^${\overline{{{{{{\rm{D}}}}}}}}^{0}$ mass spectrum the near-threshold enhancement is due to ${\chi }_{{{{{{\rm{c}}}}}}1}(3872)\,\to {{{{{{\rm{D}}}}}}}^{0}{\overline{{{{{{\rm{D}}}}}}}}^{0}{\pi }^{0}$ and ${\chi }_{{{{{{\rm{c}}}}}}1}(3872)\,\to {{{{{{\rm{D}}}}}}}^{0}{\overline{{{{{{\rm{D}}}}}}}}^{0}\gamma$ decays via intermediate D^*0^ mesons^77^. This structure is significantly wider than the structure in the D^0^D^0^ mass spectrum from ${{{{{{\rm{T}}}}}}}_{{{{{{\rm{c}}}}}}{{{{{\rm{c}}}}}}}^{+}\,\to {{{{{{\rm{D}}}}}}}^{0}{{{{{{\rm{D}}}}}}}^{0}{\pi }^{+}$ decays primarily due to the larger natural width and smaller binding energy for the *χ*_c1_(3872) state^94,95^. With more data, and with a better understanding of the dynamics of ${\chi }_{{{{{{\rm{c}}}}}}1}(3872)\,\to {{{{{{\rm{D}}}}}}}^{0}{\overline{{{{{{\rm{D}}}}}}}}^{0}{\pi }^{0}/\gamma$ decays, and therefore of the corresponding shape in the D^0^${\overline{{{{{{\rm{D}}}}}}}}^{0}$ mass spectrum, it will be possible to estimate the relative production rates for the ${{{{{{\rm{T}}}}}}}_{{{{{{\rm{c}}}}}}{{{{{\rm{c}}}}}}}^{+}$ and *χ*_c1_(3872) states. Background-subtracted D^0^D^0^*π*^+^ and D^0^D^+^ mass distributions together with those for ${\overline{{{{{{\rm{D}}}}}}}}^{0}{{{{{{\rm{D}}}}}}}^{0}{\pi }^{+}$ and D^0^D^−^ are shown in Supplementary Figs. 3 and 4.

## Discussion

The exotic narrow tetraquark state ${{{{{{\rm{T}}}}}}}_{{{{{{\rm{c}}}}}}{{{{{\rm{c}}}}}}}^{+}$ observed in Ref. ^71^ is studied using a dataset corresponding to an integrated luminosity of 9 fb^−1^, collected by the LHCb experiment in pp collisions at centre-of-mass energies of 7, 8 and 13 TeV. The observed D^0^*π*^+^ mass distribution indicates that the ${{{{{{\rm{T}}}}}}}_{{{{{{\rm{c}}}}}}{{{{{\rm{c}}}}}}}^{+}\,\to {{{{{{\rm{D}}}}}}}^{0}{{{{{{\rm{D}}}}}}}^{0}{\pi }^{+}$ decay proceeds via an intermediate off-shell D^*+^ meson. Together with the proximity of the state to the D^*^D^0^ mass threshold, this favours the spin-parity quantum numbers J^P^ to be 1^+^. Narrow near-threshold structures are observed in the D^0^D^0^ and D^0^D^+^ mass spectra with high significance. These are found to be consistent with originating from off-shell ${{{{{{\rm{T}}}}}}}_{{{{{{\rm{c}}}}}}{{{{{\rm{c}}}}}}}^{+}\,\to {{{{{{\rm{D}}}}}}}^{* }{{{{{\rm{D}}}}}}$ decays followed by the D^*^ → D*π* and D^*^ → D*γ* decays. No signal is observed in the D^+^D^0^*π*^+^ mass spectrum, and no structure is observed in the D^+^D^+^ mass spectrum. These non-observations provide a strong argument in favour of the isoscalar nature for the observed state, supporting its interpretation as the isoscalar J^P^ = 1^+^ ${{{{{\rm{c}}}}}}{{{{{\rm{c}}}}}}\overline{{{{{{\rm{u}}}}}}}\overline{{{{{{\rm{d}}}}}}}$-tetraquark ground state. A dedicated unitarised three-body Breit–Wigner amplitude is built on the assumption of strong isoscalar coupling of the axial-vector ${{{{{{\rm{T}}}}}}}_{{{{{{\rm{c}}}}}}{{{{{\rm{c}}}}}}}^{+}$ state to the D^*^D channel. This assumption is supported by the data; however, alternative models are not excluded by the distributions studied in this analysis. Probing alternative models and the validity of the underlying assumptions of this analysis will be a subject for future studies.

Using the developed amplitude model, the mass of the ${{{{{{\rm{T}}}}}}}_{{{{{{\rm{c}}}}}}{{{{{\rm{c}}}}}}}^{+}$ state, relative to the D^*+^D^0^ mass threshold, is determined to be

21 $$\delta {m}_{{{{{{\rm{U}}}}}}}=-359\pm 4{0}_{-6}^{+9}\,{{{{{\rm{keV}}}}}}/{c}^{2},$$

where the first uncertainty is statistic and the second systematic. The lower limit on the absolute value of the coupling constant of the ${{{{{{\rm{T}}}}}}}_{{{{{{\rm{c}}}}}}{{{{{\rm{c}}}}}}}^{+}$ state to the D^*^D system is

22 $$\left|g\right| \, > \, 5.1\,(4.3)\,{{{{{\rm{GeV}}}}}}\,{{{{{\rm{at}}}}}}\,90\,(95)\, \% \,{{{{{\rm{CL}}}}}}.$$

Using the same model, the estimates for the scattering length *a*, effective range *r*, and the compositeness, *Z* are obtained from the low-energy limit of the amplitude to be

23 $$a=\left[-\left(7.16\pm 0.51\right)+i\left(1.85\pm 0.28\right)\right]\,{{{{{\rm{fm}}}}}},$$

24 $$-r \, < \, 11.9\,(16.9)\,{{{{{\rm{fm}}}}}}\,{{{{{\rm{at}}}}}}\,90\,(95) \% \,{{{{{\rm{CL}}}}}},$$

25 $$Z \, < \, 0.52\,(0.58)\,{{{{{\rm{at}}}}}}\,90\,(95) \% \,{{{{{\rm{CL}}}}}}.$$

The characteristic size calculated from the binding energy is *R*_Δ*E*_ = 7.49 ± 0.42 fm. This value is consistent with the estimation from the scattering length, *R*_*a*_ = 7.16 ± 0.51 fm. Both *R*_Δ*E*_ and *R*_*a*_ correspond to a spatial extension significantly exceeding the typical scale for heavy-flavour hadrons. Within this model the resonance pole is found to be located on the second Riemann sheet with respect to the D^0^D^0^*π*^+^ threshold, at $\hat{s}={m}_{{{{{{\rm{pole}}}}}}}-\frac{i}{2}{{{\Gamma }}}_{{{{{{\rm{pole}}}}}}}$, where

26 $$\delta {m}_{{{{{{\rm{pole}}}}}}}=-360\pm 4{0}_{-0}^{+4}\,{{{{{\rm{keV}}}}}}/{c}^{2},$$

27 $${{{\Gamma }}}_{{{{{{\rm{pole}}}}}}}=48\pm {2}_{-14}^{+0}\,{{{{{\rm{keV}}}}}},$$

where the first uncertainty accounts for statistical and systematic uncertainties for the *δm*_U_ parameters, and the second is due to the unknown value of the $\left|g\right|$ parameter. The pole position, scattering length, effective range and compositeness form a complete set of observables related to the ${{{{{{\rm{T}}}}}}}_{{{{{{\rm{c}}}}}}{{{{{\rm{c}}}}}}}^{+}\,\to {{{{{{\rm{D}}}}}}}^{0}{{{{{{\rm{D}}}}}}}^{0}{\pi }^{+}$ reaction amplitude, which are crucial for inferring the nature of the ${{{{{{\rm{T}}}}}}}_{{{{{{\rm{c}}}}}}{{{{{\rm{c}}}}}}}^{+}$ tetraquark.

Unlike in the prompt production of the *χ*_c1_(3872) state, no suppression of the ${{{{{{\rm{T}}}}}}}_{{{{{{\rm{c}}}}}}{{{{{\rm{c}}}}}}}^{+}$ production at high track multiplicities is observed relative to the low-mass ${{{{{{\rm{D}}}}}}}^{0}{\overline{{{{{{\rm{D}}}}}}}}^{0}$ pairs. The observed similarity with the multiplicity distribution for the low-mass D^0^D^0^ production process, that is presumably double-parton-scattering dominated, is unexpected. In the future with a larger dataset and including other decay modes, e.g., D^0^ → K^−^*π*^+^*π*^+^*π*^−^, detailed studies of the properties of this new state and its production mechanisms could be possible.

In conclusion, the ${{{{{{\rm{T}}}}}}}_{{{{{{\rm{c}}}}}}{{{{{\rm{c}}}}}}}^{+}$ tetraquark observed in D^0^D^0^*π*^+^ decays is studied in detail, using a unitarised model that accounts for the relevant thresholds by taking into account the D^0^D^0^*π*^+^ and D^0^D^+^*π*^0^(*γ*) decay channels with intermediate D^*^ resonances. This model is found to give an excellent description of the D^0^*π*^+^ mass distribution in the ${{{{{{\rm{T}}}}}}}_{{{{{{\rm{c}}}}}}{{{{{\rm{c}}}}}}}^{+}\,\to {{{{{{\rm{D}}}}}}}^{0}{{{{{{\rm{D}}}}}}}^{0}{\pi }^{+}$ decay and of the threshold enhancements observed in the D^0^D^0^ and D^0^D^+^ spectra. Together with the absence of a signal in the D^0^D^+^ and D^+^D^0^*π*^+^ mass distributions this provides a strong argument for interpreting the observed state as the isoscalar ${{{{{{\rm{T}}}}}}}_{{{{{{\rm{c}}}}}}{{{{{\rm{c}}}}}}}^{+}$ tetraquark with spin-parity J^P^ = 1^+^. The precise ${{{{{{\rm{T}}}}}}}_{{{{{{\rm{c}}}}}}{{{{{\rm{c}}}}}}}^{+}$ mass measurement will rule out or improve on a considerable range of theoretical models on heavy quark systems. The determined pole position and physical quantities derived from low-energy scattering parameters reveal important information about the nature of the ${{{{{{\rm{T}}}}}}}_{{{{{{\rm{c}}}}}}{{{{{\rm{c}}}}}}}^{+}$ tetraquark. In addition, the counter-intuitive dependence of the production rate on track multiplicity will pose a challenge for theoretical explanations.

## Methods

### Experimental setup

The LHCb detector^72,73^ is a single-arm forward spectrometer covering the pseudorapidity range 2 < *η* < 5, designed for the study of particles containing b or c quarks. The detector includes a high-precision tracking system consisting of a silicon-strip vertex detector surrounding the pp interaction region, a large-area silicon-strip detector located upstream of a dipole magnet with a bending power of about 4 Tm, and three stations of silicon-strip detectors and straw drift tubes placed downstream of the magnet. The tracking system provides a measurement of the momentum, *p*, of charged particles with a relative uncertainty that varies from 0.5% at low momentum to 1.0% at 200 GeV/*c*. The minimum distance of a track to a primary pp collision vertex, the impact parameter, is measured with a resolution of (15 + 29/*p*_T_) μm, where *p*_T_ is the component of the momentum transverse to the beam, in GeV/*c*. Different types of charged hadrons are distinguished using information from two ring-imaging Cherenkov detectors^128^. Photons, electrons and hadrons are identified by a calorimeter system consisting of scintillating-pad and preshower detectors, an electromagnetic and a hadronic calorimeter. Muons are identified by a system composed of alternating layers of iron and multiwire proportional chambers. The online event selection is performed by a trigger, which consists of a hardware stage, based on information from the calorimeter and muon systems, followed by a software stage, which applies a full event reconstruction.

### Simulation

Simulation is required to model the effects of the detector acceptance, resolution, and the efficiency of the imposed selection requirements. In the simulation, pp collisions are generated using PYTHIA^129^ with a specific LHCb configuration^130^. Decays of unstable particles are described by EVTGEN^131^, in which final-state radiation is generated using PHOTOS^132^. The interaction of the generated particles with the detector, and its response, are implemented using the GEANT4 toolkit^133,134^ as described in Ref. ^135^.

### Event selection

The D^0^D^0^, D^0^D^+^ and D^0^D^0^*π*^+^ final states are reconstructed using the D^0^ → K^−^*π*^+^ and D^+^ → K^−^*π*^+^*π*^+^ decay channels. The selection criteria are similar to those used in Refs. ^74–77^. Kaons and pions are selected from well-reconstructed tracks within the acceptance of the spectrometer that are identified using information from the ring-imaging Cherenkov detectors. The kaon and pion candidates that have transverse momenta larger than 250 MeV/*c* and are inconsistent with being produced at a pp interaction vertex are combined together to form D^0^ and D^+^ candidates, referred to as D hereafter. The resulting D candidates are required to have good vertex quality, mass within ±65 and ±50 MeV/*c*^2^ of the known D^0^ and D^+^ masses^10^, respectively, transverse momentum larger than 1 GeV/*c*, decay time larger than 100 μm/*c* and a momentum direction that is consistent with the vector from the primary to secondary vertex. Selected D^0^ and D^+^ candidates consistent with originating from a common primary vertex are combined to form D^0^D^0^ and D^0^D^+^ candidates. The resulting D^0^D^0^ candidates are combined with a pion to form D^0^D^0^*π*^+^ candidates. At least one of the two D^0^*π*^+^ combinations is required to have good vertex quality and mass not exceeding the known D^*+^ mass by more than 155 MeV/*c*^2^. For each D^0^D^0^, D^0^D^+^ and D^0^D^0^*π*^+^ candidate a kinematic fit^136^ is performed. This fit constrains the mass of the D candidates to their known values and requires both D mesons, and a pion in the case of D^0^D^0^*π*^+^, to originate from the same primary vertex. A requirement is applied to the quality of this fit to further suppress combinatorial background and reduce background from D candidates produced in two independent pp interactions or in the decays of beauty hadrons^74^. To suppress background from kaon and pion candidates reconstructed from a common track, all track pairs of the same charge are required to have an opening angle inconsistent with zero and the mass of the combination must be inconsistent with the sum of the masses of the two constituents. For cross-checks additional final states D^+^D^+^, D^+^D^0^*π*^+^, ${{{{{{\rm{D}}}}}}}^{0}{\overline{{{{{{\rm{D}}}}}}}}^{0}$, D^0^D^−^ and ${\overline{{{{{{\rm{D}}}}}}}}^{0}{{{{{{\rm{D}}}}}}}^{0}{\pi }^{+}$ are reconstructed, selected and treated in the same way.

### Non-D background subtraction

Two-dimensional distributions of the mass of one D candidate versus the mass of the other D candidate from selected D^0^D^0^*π*^+^, D^0^D^0^ and D^0^D^+^ combinations are shown in Supplementary Fig. 1. These distributions illustrate the relatively small combinatorial background levels due to fake D candidates. This background is subtracted using the *s*Plot technique^137^, which is based on an extended unbinned maximum-likelihood fit to these two-dimensional distributions with the function described in Ref. ^74^.

This function consists of four components:

- a component corresponding to genuine D_1_D_2_ pairs and described as a product of two signal functions, each parameterised with a modified Novosibirsk function^107^;
- two components corresponding to combinations of one of the D mesons with combinatorial background, described as a product of the signal function and a background function, which is parameterised with a product of an exponential function and a positive first-order polynomial;
- a component corresponding to pure background pairs and described by a product of exponential functions and a positive two-dimensional non-factorisable second-order polynomial function.

Based on the results of the fit, each candidate is assigned a positive weight for being signal-like or a negative weight for being background-like, with the masses of the two D^0^ candidates as discriminating variables. The D^0^D^0^*π*^+^ mass distributions for each of the subtracted background components are presented in Supplementary Fig. 2. where fit results with background-only functions ${B}_{10}^{\prime}$, defined in Eq. (3) are overlaid.

### Resolution model for the D^0^D^0^*π*^+^ mass

In the vicinity of the D^*+^D^0^ mass threshold the resolution function ${\mathfrak{R}}$ for the D^0^D^0^*π*^+^ mass is parametrised with the sum of two Gaussian functions with a common mean. The widths of the Gaussian functions are *σ*_1_ = 1.05 × 263 keV/*c*^2^ and *σ*_2_ = 2.413 × *σ*_1_ for the narrow and wide components, respectively, and the fraction of the narrow Gaussian is *α* = 0.778. The parameters *α* and *σ*_1,2_ are taken from simulation, and *σ*_1,2_ are corrected with a factor of 1.05 that accounts for a small difference between simulation and data for the mass resolution^94–96^. The RMS of the resolution function is around 400 keV/*c*^2^.

### Matrix elements for ${{{{{{\rm{T}}}}}}}_{{{{{{\rm{c}}}}}}{{{{{\rm{c}}}}}}}^{+}\,\to {{{{{\rm{D}}}}}}{{{{{\rm{D}}}}}}\pi /\gamma$ decays

Assuming isospin symmetry, the isoscalar vector state ${{{{{{\rm{T}}}}}}}_{{{{{{\rm{c}}}}}}{{{{{\rm{c}}}}}}}^{+}$ that decays into the D^*^D final state can be expressed as

28 $$\left|{{{{{{\rm{T}}}}}}}_{{{{{{\rm{c}}}}}}{{{{{\rm{c}}}}}}}^{+}\right\rangle =\frac{1}{\sqrt{2}}\left(\left|{{{{{{\rm{D}}}}}}}^{* +}{{{{{{\rm{D}}}}}}}^{0}\right\rangle -\left|{{{{{{\rm{D}}}}}}}^{* 0}{{{{{{\rm{D}}}}}}}^{+}\right\rangle \right).$$

Therefore, the S-wave amplitudes for the ${{{{{{\rm{T}}}}}}}_{{{{{{\rm{c}}}}}}{{{{{\rm{c}}}}}}}^{+}\,\to {{{{{{\rm{D}}}}}}}^{* +}{{{{{{\rm{D}}}}}}}^{0}$ and ${{{{{{\rm{T}}}}}}}_{{{{{{\rm{c}}}}}}{{{{{\rm{c}}}}}}}^{+}\,\to {{{{{{\rm{D}}}}}}}^{* 0}{{{{{{\rm{D}}}}}}}^{+}$ decays have different signs

29 $${{{{{{\mathcal{A}}}}}}}_{{{{{{{\rm{T}}}}}}}_{{{{{{\rm{c}}}}}}{{{{{\rm{c}}}}}}}^{+}\,\to {{{{{{\rm{D}}}}}}}^{* +}{{{{{{\rm{D}}}}}}}^{0}}^{{{{{{\rm{S-wave}}}}}}}=+\frac{g}{\sqrt{2}}{\epsilon }_{{{{{{{\rm{T}}}}}}}_{{{{{{\rm{c}}}}}}{{{{{\rm{c}}}}}}}^{+}\mu }{\epsilon }_{{{{{{{\rm{D}}}}}}}^{* }}^{* \mu },$$

30 $${{{{{{\mathcal{A}}}}}}}_{{{{{{{\rm{T}}}}}}}_{{{{{{\rm{c}}}}}}{{{{{\rm{c}}}}}}}^{+}\,\to {{{{{{\rm{D}}}}}}}^{* 0}{{{{{{\rm{D}}}}}}}^{+}}^{{{{{{\rm{S-wave}}}}}}}=-\frac{g}{\sqrt{2}}{\epsilon }_{{{{{{{\rm{T}}}}}}}_{{{{{{\rm{c}}}}}}{{{{{\rm{c}}}}}}}^{+}\mu }{\epsilon }_{{{{{{{\rm{D}}}}}}}^{* }}^{* \mu },$$

where *g* is a coupling constant, ${\epsilon }_{{{{{{{\rm{T}}}}}}}_{{{{{{\rm{c}}}}}}{{{{{\rm{c}}}}}}}^{+}}$ is the polarisation vector of the ${{{{{{\rm{T}}}}}}}_{{{{{{\rm{c}}}}}}{{{{{\rm{c}}}}}}}^{+}$ particle and ${\epsilon }_{{{{{{{\rm{D}}}}}}}^{* }}$ is the polarisation vector of D^*^ meson, and the upper and lower Greek indices imply the summation in the Einstein notation. The S-wave (corresponding to orbital angular momentum equal to zero) approximation is valid for a near-threshold peak. For ${{{{{{\rm{T}}}}}}}_{{{{{{\rm{c}}}}}}{{{{{\rm{c}}}}}}}^{+}$ masses significantly above the D^*^D threshold, higher-order waves also need to be considered. The amplitudes for the D^*^ → D*π* decays are written as

31 $${{{{{{\mathcal{A}}}}}}}_{{{{{{{\rm{D}}}}}}}^{* +}\to {{{{{{\rm{D}}}}}}}^{0}{\pi }^{+}}=f{\epsilon }_{{{{{{{\rm{D}}}}}}}^{* }}^{\alpha }{p}_{{{{{{\rm{D}}}}}}\alpha }$$

32 $$\begin{array}{rcl}{{{{{{\mathcal{A}}}}}}}_{{{{{{{\rm{D}}}}}}}^{* +}\to {{{{{{\rm{D}}}}}}}^{+}{\pi }^{0}}&=&-\frac{f}{\sqrt{2}}{\epsilon }_{{{{{{{\rm{D}}}}}}}^{* }}^{\alpha }{p}_{{{{{{\rm{D}}}}}}\alpha }\end{array}$$

33 $$\begin{array}{rcl}{{{{{{\mathcal{A}}}}}}}_{{{{{{{\rm{D}}}}}}}^{* 0}\to {{{{{{\rm{D}}}}}}}^{0}{\pi }^{0}}&=&+\frac{f}{\sqrt{2}}{\epsilon }_{{{{{{{\rm{D}}}}}}}^{* }}^{\alpha }{p}_{{{{{{\rm{D}}}}}}\alpha },\end{array}$$

where *f* denotes a coupling constant, and *p*_D_ stands for the momentum of the D meson. The amplitude for the D^*^ → D*γ* decays is

34 $${{{{{{\mathcal{A}}}}}}}_{{{{{{{\rm{D}}}}}}}^{* }\to \gamma {{{{{\rm{D}}}}}}}=i\mu h{\epsilon }_{\alpha \beta \eta \xi }{\epsilon }_{{{{{{{\rm{D}}}}}}}^{* }}^{\alpha }{p}_{{{{{{{\rm{D}}}}}}}^{* }}^{\beta }{\epsilon }_{\gamma }^{* \eta }{p}_{\gamma }^{\xi },$$

where *h* denotes a coupling constant, *μ* stands for the magnetic moment for D^*^ → D*γ* transitions, ${p}_{{{{{{{\rm{D}}}}}}}^{* }}$ and *p*_*γ*_ are the D^*^-meson and photon momenta, respectively, and *ϵ*_*γ*_ is the polarisation vector of the photon. The three amplitudes for ${{{{{{\rm{T}}}}}}}_{{{{{{\rm{c}}}}}}{{{{{\rm{c}}}}}}}^{+}\,\to \pi {{{{{\rm{D}}}}}}{{{{{\rm{D}}}}}}$ and ${{{{{{\rm{T}}}}}}}_{{{{{{\rm{c}}}}}}{{{{{\rm{c}}}}}}}^{+}\,\to \gamma {{{{{\rm{D}}}}}}{{{{{\rm{D}}}}}}$ decays are

35 $${{{{{{\mathcal{A}}}}}}}_{{\pi }^{+}{{{{{{\rm{D}}}}}}}^{0}{{{{{{\rm{D}}}}}}}^{0}}=\frac{fg}{\sqrt{2}}{\epsilon }_{{{{{{{\rm{T}}}}}}}_{{{{{{\rm{c}}}}}}{{{{{\rm{c}}}}}}}^{+}\nu }\left[{{\mathfrak{F}}}_{+}({s}_{12})\times \left(-{p}_{2}^{\nu }+\frac{({p}_{2}{p}_{12}){p}_{12}^{\nu }}{{s}_{12}}\right)+({p}_{2}\leftrightarrow {p}_{3})\right],$$

36 $$\begin{array}{rcl}{{{{{{\mathcal{A}}}}}}}_{{\pi }^{0}{{{{{{\rm{D}}}}}}}^{+}{{{{{{\rm{D}}}}}}}^{0}}&=&-\frac{fg}{2}{\epsilon }_{{{{{{{\rm{T}}}}}}}_{{{{{{\rm{c}}}}}}{{{{{\rm{c}}}}}}}^{+}\nu }\left[{{\mathfrak{F}}}_{+}({s}_{12})\times \left(-{p}_{2}^{\nu }+\frac{({p}_{2}{p}_{12}){p}_{12}^{\nu }}{{s}_{12}}\right)+\left(\begin{array}{c}{p}_{2}\leftrightarrow {p}_{3}\\ {{\mathfrak{F}}}_{+}\leftrightarrow {{\mathfrak{F}}}_{0}\end{array}\right)\right],\\ \end{array}$$

37 $${{{{{{\mathcal{A}}}}}}}_{\gamma {{{{{{\rm{D}}}}}}}^{+}{{{{{{\rm{D}}}}}}}^{0}}=i\frac{hg}{\sqrt{2}}{\epsilon }_{\alpha \beta \eta \xi }{\epsilon }_{{{{{{{\rm{T}}}}}}}_{{{{{{\rm{c}}}}}}{{{{{\rm{c}}}}}}}^{+}}^{\beta }{\epsilon }_{\gamma }^{\eta }{p}_{\gamma }^{\xi }\left[{\mu }_{+}{{\mathfrak{F}}}_{+}({s}_{12}){p}_{12}^{\alpha }-{\mu }_{0}{{\mathfrak{F}}}_{0}({s}_{13}){p}_{13}^{\alpha }\right],$$

where ${s}_{ij}={p}_{ij}^{2}={({p}_{i}+{p}_{j})}^{2}$ and the ${\mathfrak{F}}$ functions that denote the Breit–Wigner amplitude for the D^*^ mesons are

38 $${\mathfrak{F}}(s)=\frac{1}{{m}_{{{{{{{\rm{D}}}}}}}^{* }}^{2}-s-i{m}_{{{{{{{\rm{D}}}}}}}^{* }}{{{\Gamma }}}_{{{{{{{\rm{D}}}}}}}^{* }}}.$$

A small possible distortion of the Breit–Wigner shape of the D^*^ meson due to three-body final-state interactions is neglected in the model. The impact of the energy-dependence of the D^*^ meson self-energy is found to be insignificant. The decays of the ${{{{{{\rm{T}}}}}}}_{{{{{{\rm{c}}}}}}{{{{{\rm{c}}}}}}}^{+}$ state into the D^+^D^+^*π*^−^ final state via off-shell D^*0^ → D^+^*π*^−^ decays are highly suppressed and are not considered here. The last terms in Eqs. (35) and (36) imply the same amplitudes with swapped momenta.

The ${{{{{{\rm{T}}}}}}}_{{{{{{\rm{c}}}}}}{{{{{\rm{c}}}}}}}^{+}$ state is assumed to be produced unpolarised; therefore, the squared absolute value of the decay amplitudes with pions in the final state, averaged over the initial spin state are

39 $${\left|{{\mathfrak{M}}}_{{\pi }^{+}{{{{{{\rm{D}}}}}}}^{0}{{{{{{\rm{D}}}}}}}^{0}}\right|}^{2}=\frac{1}{3}\frac{{f}^{2}{g}^{2}}{2!\cdot 2}\left[{\left|{{\mathfrak{F}}}_{+}({s}_{12})\right|}^{2}A+{\left|{{\mathfrak{F}}}_{+}({s}_{13})\right|}^{2}B+2\Re \left\{{{\mathfrak{F}}}_{+}({s}_{12}){{\mathfrak{F}}}_{+}^{* }({s}_{13})\right\}C\right],$$

40 $${\left|{{\mathfrak{M}}}_{{\pi }^{0}{{{{{{\rm{D}}}}}}}^{+}{{{{{{\rm{D}}}}}}}^{0}}\right|}^{2}=\frac{1}{3}\frac{{f}^{2}{g}^{2}}{4}\left[{\left|{{\mathfrak{F}}}_{+}({s}_{12})\right|}^{2}A+{\left|{{\mathfrak{F}}}_{0}({s}_{13})\right|}^{2}B+2\Re \left\{{{\mathfrak{F}}}_{+}({s}_{12}){{\mathfrak{F}}}_{0}^{* }({s}_{13})\right\}C\right],$$

where

41 $$A=\frac{\lambda ({s}_{12},{m}_{1}^{2},{m}_{2}^{2})}{4{s}_{12}}+\frac{1}{s}{\left(\frac{(p{p}_{12})({p}_{2}{p}_{12})}{{s}_{12}}-p{p}_{2}\right)}^{2},$$

42 $$B=\frac{\lambda ({s}_{13},{m}_{1}^{2},{m}_{3}^{2})}{4{s}_{13}}+\frac{1}{s}{\left(\frac{(p{p}_{13})({p}_{3}{p}_{13})}{{s}_{13}}-p{p}_{3}\right)}^{2},$$

$$C=D+E$$

43 $$D=\frac{({p}_{3}{p}_{12})({p}_{2}{p}_{12})}{{s}_{12}}+\frac{({p}_{2}{p}_{13})({p}_{3}{p}_{13})}{{s}_{13}}-\frac{({p}_{12}{p}_{13})({p}_{2}{p}_{12})({p}_{3}{p}_{13})}{{s}_{12}{s}_{13}}-{p}_{2}{p}_{3},$$

44 $$E=	\frac{(p{p}_{12})(p{p}_{13})({p}_{2}{p}_{12})({p}_{3}{p}_{13})}{s{s}_{12}{s}_{13}}+\frac{(p{p}_{2})(p{p}_{3})}{s}\\ 	 -\frac{(p{p}_{12})({p}_{2}{p}_{12})(p{p}_{3})}{s{s}_{12}}-\frac{(p{p}_{13})({p}_{3}{p}_{13})(p{p}_{2})}{s{s}_{13}},$$

and $\lambda \left(x,y,z\right)$ stands for the Källén function^97^. The additional factor of 2! in the denominator of Eq. (39) is due to the presence of two identical particles (D^0^) in the final state. The squared absolute values of the decay amplitude with a photon in the final state, averaged over the initial spin state is

45 $${\left|{{\mathfrak{M}}}_{{{{{{{\rm{T}}}}}}}_{{{{{{\rm{c}}}}}}{{{{{\rm{c}}}}}}}^{+}\to \gamma {{{{{{\rm{D}}}}}}}^{+}{{{{{{\rm{D}}}}}}}^{0}}\right|}^{2}=	\,\frac{1}{3}{\left|gh\right|}^{2}{\left|{\mu }_{+}{{\mathfrak{F}}}_{+}({s}_{12})({p}_{1}{p}_{2})-{\mu }_{0}{{\mathfrak{F}}}_{0}({s}_{13})({p}_{1}{p}_{3})\right|}^{2}\\ 	+\frac{1}{3}{\left|gh\right|}^{2}{\left|{\mu }_{+}{{\mathfrak{F}}}_{+}({s}_{12})+{\mu }_{0}{{\mathfrak{F}}}_{0}({s}_{13})\right|}^{2}G,$$

46 $$\begin{array}{rcl}G&=&\frac{1}{2s}\left[2({p}_{1}{p}_{2})({p}_{1}{p}_{3})({p}_{2}{p}_{3})-{m}_{2}^{2}{({p}_{1}{p}_{3})}^{2}-{m}_{3}^{2}{({p}_{1}{p}_{2})}^{2}\right].\end{array}$$

The coupling constants *f* and *h* for the D^*^ → D*π* and D^*^ → D*γ* decays are calculated using Eqs. (31)–(34), from the known branching fractions of the D^*^ → D*π* and D^*^ → D*γ* decays^10^, the measured natural width of the D^*+^ meson^10,112^ and the derived value for the natural width for the D^*0^ meson^66,81,113^. The magnetic moment *μ*_+_ is taken to be 1 and the ratio of magnetic moments *μ*_0_/*μ*_+_ is calculated according to Refs. ^138–140^.

### Unitarised Breit–Wigner shape

A unitarised three-body Breit–Wigner function is defined as

47 $${{\mathfrak{F}}}_{f}^{{{{{{\rm{U}}}}}}}\left(s\right)={\varrho }_{f}\left(s\right){\left|{{{{{{\mathcal{A}}}}}}}_{{{{{{\rm{U}}}}}}}\left(s\right)\right|}^{2},$$

48 $${{{{{{\mathcal{A}}}}}}}_{{{{{{\rm{U}}}}}}}\left(s\right)=\frac{1}{{m}_{{{{{{\rm{U}}}}}}}^{2}-s-i{m}_{{{{{{\rm{U}}}}}}}\hat{{{\Gamma }}}(s)},$$

where $f\in \left\{{{{{{{\rm{D}}}}}}}^{0}{{{{{{\rm{D}}}}}}}^{0}{\pi }^{+},{{{{{{\rm{D}}}}}}}^{0}{{{{{{\rm{D}}}}}}}^{+}{\pi }^{0},{{{{{{\rm{D}}}}}}}^{0}{{{{{{\rm{D}}}}}}}^{+}\gamma \right\}$ denotes the final state. The decay matrix element for each channel integrated over the three-body phase-space is denoted by

49 $${\varrho }_{f}(s)=\frac{1}{{(2\pi )}^{5}}\frac{{\pi }^{2}}{4s}\iint d{s}_{12}d{s}_{23}\frac{{\left|{{\mathfrak{M}}}_{f}\left(s,{s}_{12},{s}_{23}\right)\right|}^{2}}{{\left|g\right|}^{2}},$$

where ${\left|{{\mathfrak{M}}}_{f}\right|}^{2}$ is defined by Eqs. (39)–(46) and the unknown coupling constant *g* is taken out of the expression for ${\left|{{\mathfrak{M}}}_{f}\right|}^{2}$. For large values of *s*, in excess of s^*^, such as $\sqrt{{s}^{* }}-\left({m}_{{{{{{{\rm{D}}}}}}}^{* }}+{m}_{{{{{{\rm{D}}}}}}}\right)\gg {{{\Gamma }}}_{{{{{{{\rm{D}}}}}}}^{* }}$, the functions *ϱ*_*f*_(*s*) are defined as

50 $${\left.{\varrho }_{{{{{{{\rm{D}}}}}}}^{0}{{{{{{\rm{D}}}}}}}^{0}{\pi }^{+}}(s)\right|}_{s { > }{s}^{* }}={c}_{1}{{{\Phi }}}_{{{{{{{\rm{D}}}}}}}^{* +}{{{{{{\rm{D}}}}}}}^{0}}(s),$$

51 $${\left.{\varrho }_{{{{{{{\rm{D}}}}}}}^{0}{{{{{{\rm{D}}}}}}}^{+}{\pi }^{0}}(s)\right|}_{s { > }{s}^{* }}={c}_{2}{{{\Phi }}}_{{{{{{{\rm{D}}}}}}}^{* 0}{{{{{{\rm{D}}}}}}}^{+}}(s),$$

52 $${\left.{\varrho }_{{{{{{{\rm{D}}}}}}}^{0}{{{{{{\rm{D}}}}}}}^{+}\gamma }(s)\right|}_{s { > }{s}^{* }}={c}_{3}{{{\Phi }}}_{{{{{{{\rm{D}}}}}}}^{* 0}{{{{{{\rm{D}}}}}}}^{+}}(s),$$

where ${{{\Phi }}}_{{{{{{{\rm{D}}}}}}}^{* }{{{{{\rm{D}}}}}}}(s)$ denotes the two-body phase-space function, the constants *c*_1_,*c*_2_ and *c*_3_ are chosen to ensure the continuity of the functions *ϱ*_*f*_(*s*), and a value of $\sqrt{{s}^{* }}=3.9\,{{{{{\rm{GeV}}}}}}/{c}^{2}$ is used. The functions *ϱ*_*f*_(*s*) are shown in Supplementary Fig. 5. The complex-valued width $\hat{{{\Gamma }}}(s)$ is defined via the self-energy function Σ(*s*)^141^

53 $$i{m}_{{{{{{\rm{U}}}}}}}\hat{{{\Gamma }}}(s)\equiv {\left|g\right|}^{2}{{\Sigma }}\,(s),$$

where ${\left|g\right|}^{2}$ is again factored out for convenience. The imaginary part of Σ(*s*) for real physical values of *s* is computed through the optical theorem as half of the sum of the decay probability to all available channels^142^:

54 $$\Im \,{\left.{{\Sigma }}(s)\right|}_{\Im s = {0}^{+}}=\frac{1}{2}{\varrho }_{{{{{{\rm{tot}}}}}}}(s),$$

55 $${\varrho }_{{{{{{\rm{tot}}}}}}}\left(s\right)\equiv \mathop{\sum}\limits_{f}{\varrho }_{f}\left(s\right).$$

The real part of the self-energy function is computed using Kramers–Kronig dispersion relations with a single subtraction^143,144^,

56 $${\left.\Re {{\Sigma }}(s)\right|}_{\Im s = {0}^{+}}=\xi (s)-\xi ({m}_{{{{{{\rm{U}}}}}}}^{2}),$$

57 $$\xi (s)=\frac{s}{2\pi }\,{{{{{\rm{p.v.}}}}}}\,\,\,\int\limits_{{s}_{{{{{{\rm{th}}}}}}}^{* }}^{+\infty }\frac{{\varrho }_{{{{{{\rm{tot}}}}}}}({s}^{\prime})}{{s}^{\prime}\left({s}^{\prime}-s\right)}d{s}^{\prime},$$

where the Cauchy principal value (p.v.) integral over *ϱ*_tot_(*s*) is understood as

58 $${{{{{\rm{p.v.}}}}}}\,\,\,\int\limits_{{s}_{{{{{{\rm{th}}}}}}}^{* }}^{+\infty }ds\,{\varrho }_{{{{{{\rm{tot}}}}}}}(s)\,...\equiv {\sum }_{f}{{{{{\rm{p.v.}}}}}}\,\,\,\int\limits_{{s}_{f}}^{+\infty }ds\,{\varrho }_{f}(s)\,...,$$

and *s*_*f*_ denotes the threshold value for the channel *f*. The subtraction is needed since the integral ∫*ϱ*_tot_(*s*)/*s* *ds* diverges. The term $\xi ({m}_{{{{{{\rm{U}}}}}}}^{2})$ in Eq. (56) corresponds to the choice of subtraction constant such that $\Re \,{{{{{{\mathcal{A}}}}}}}_{{{{{{\rm{U}}}}}}}({m}_{{{{{{\rm{U}}}}}}}^{2})=0$. The function *ξ*(*s*) is shown in Supplementary Fig. 6.

Alternatively, the isoscalar amplitude ${{{{{{\mathcal{A}}}}}}}_{{{{{{\rm{U}}}}}}}$ is constructed using the *K*-matrix approach^145^ with two coupled channels, D^*+^D^0^ and D^*0^D^+^. The relation reads:

59 $${{{{{{\mathcal{A}}}}}}}_{{{{{{\rm{U}}}}}}}\left(\begin{array}{r}g\\ -g\end{array}\right)={[1-KG]}^{-1}P,$$

where a production vector *P* and an isoscalar potential *K* are defined as

60 $$P=\frac{1}{{m}_{{{{{{\rm{U}}}}}}}^{2}-s}\left(\begin{array}{l}g\\ -g\end{array}\right),\quad K(s)=\frac{1}{{m}_{{{{{{\rm{U}}}}}}}^{2}-s}\left(\begin{array}{rc}{\left|g\right|}^{2}&-{\left|g\right|}^{2}\\ -{\left|g\right|}^{2}&{\left|g\right|}^{2}\end{array}\right).$$

The propagation matrix *G* describes the D^*^D → D^*^D rescattering via the virtual loops including the one-particle exchange process^91^ and expressed in a symbolical way in Supplementary Eq. (1). where suppressed D^*0^ → D^+^*π*^−^ transition is neglected. The D^*+^D^0^ ↔ D^*0^D^+^ rescattering occurs due to non-diagonal element of the *K*-matrix (contact interaction) and non-diagonal elements of the *G* matrix (long-range interaction). The matrix *G* and the self-energy function Σ(*s*) from Eqs. (54) and (56), are related as

61 $${\left|g\right|}^{2}{{\Sigma }}(s)=\left(\begin{array}{rc}{g}^{* }&-{g}^{* }\end{array}\right)G\left(\begin{array}{r}g\\ -g\end{array}\right).$$

Similar to the Flatté function^98^ for large values of the $\left|g\right|$ parameter, the ${{\mathfrak{F}}}^{{{{{{\rm{U}}}}}}}$ signal profile exhibits a scaling property^94,99^. For large values of the $\left|g\right|$ parameter the width approaches asymptotic behaviour, see Supplementary Fig. 7. The unitarised three-body Breit–Wigner function ${{\mathfrak{F}}}^{{{{{{\rm{U}}}}}}}$ for ${{{{{{\rm{T}}}}}}}_{{{{{{\rm{c}}}}}}{{{{{\rm{c}}}}}}}^{+}\,\to {{{{{{\rm{D}}}}}}}^{0}{{{{{{\rm{D}}}}}}}^{0}{\pi }^{+}$ decays with parameters *m*_U_ and $\left|g\right|$ obtained from the fit to data is shown in Supplementary Fig. 8. The inset illustrates the similarity of the profile with the single-pole profile in the vicinity of the pole

62 $$\sqrt{\hat{s}}={\mathfrak{m}}-\frac{i}{2}{\mathfrak{w}},$$

where ${\mathfrak{m}}$ and ${\mathfrak{w}}$ are the mode and FWHM, respectively.

### Analytic continuation

Equation (56) defines Σ(*s*) and the amplitude ${{{{{{\mathcal{A}}}}}}}_{{{{{{\rm{U}}}}}}}(s)$ for real values of *s*. Analytic continuation to the whole first Riemann sheet is calculated as

63 $$\begin{array}{r}{{\Sigma }}\left(s\right)=\frac{s}{2\pi }\int\limits_{{s}_{{{{{{\rm{th}}}}}}}^{* }}^{+\infty }\frac{{\varrho }_{{{{{{\rm{tot}}}}}}}(s^{\prime} )}{s^{\prime} \left(s^{\prime} -s\right)}\,ds^{\prime} -\xi \left({m}_{{{{{{\rm{U}}}}}}}^{2}\right),\end{array}$$

where the integral is understood as in Eq. (58). The search for the resonance pole requires knowledge of the amplitude on the second Riemann sheet denoted by ${{{{{{\mathcal{A}}}}}}}_{{{{{{\rm{U}}}}}}}^{II}$. According to the optical theorem^142^, the discontinuity of the inverse amplitude across the unitarity cut is given by $i{\left|g\right|}^{2}{\varrho }_{{{{{{\rm{tot}}}}}}}(s)$:

64 $$\begin{array}{rc}\frac{1}{{{{{{{\mathcal{A}}}}}}}_{{{{{{\rm{U}}}}}}}^{II}\left(s\right)}&={m}_{{{{{{\rm{U}}}}}}}^{2}-s-{\left|g\right|}^{2}{{\Sigma }}(s)+i{\left|g\right|}^{2}{\varrho }_{{{{{{\rm{tot}}}}}}}(s).\end{array}$$

For the complex-values *s*, the analytic continuation of *ϱ*_tot_(*s*) is needed: the phase-space integral in Eq. (49) is performed over a two-dimensional complex manifold ${{{{{\mathcal{D}}}}}}$ (see discussion on the continuation in Ref. ^146^):

65 $$\int _{{{{{{\mathcal{D}}}}}}}{\left|{\mathfrak{M}}\right|}^{2}\,d{{{\Phi }}}_{3}=\frac{1}{2\pi {(8\pi )}^{2}s}\int\limits_{{({m}_{2}+{m}_{3})}^{2}}^{{(\sqrt{s}-{m}_{1})}^{2}}d{s}_{23}\int\limits_{{s}_{12}^{-}(s,{s}_{23})}^{{s}_{12}^{+}(s,{s}_{23})}{\left|{\mathfrak{M}}\right|}^{2}\,d{s}_{12},$$

where the limits of the second integral represent the Dalitz plot borders^111^,

66 $${s}_{12}^{\pm }\left(s,{s}_{23}\right)	={m}_{1}^{2}+{m}_{2}^{2}-\frac{\left({s}_{23}-s+{m}_{1}^{2}\right)\left({s}_{23}+{m}_{2}^{2}-{m}_{3}^{2}\right)}{2{s}_{23}}\\ 	 \pm \frac{{\lambda }^{1/2}\left({s}_{23},s,{m}_{1}^{2}\right){\lambda }^{1/2}\left({s}_{23},{m}_{2}^{2},{m}_{3}^{2}\right)}{2{s}_{23}}.$$

The integration is performed along straight lines connecting the end points in the complex plane.

### DD spectra from ${{{{{{\rm{T}}}}}}}_{{{{{{\rm{c}}}}}}{{{{{\rm{c}}}}}}}^{+}\,\to {{{{{\rm{D}}}}}}{{{{{\rm{D}}}}}}\pi /\gamma$ decays

The shapes of the D^0^D^0^ and D^+^D^−^ mass spectra from ${{{{{{\rm{T}}}}}}}_{{{{{{\rm{c}}}}}}{{{{{\rm{c}}}}}}}^{+}\,\to {{{{{\rm{D}}}}}}{{{{{\rm{D}}}}}}\pi /\gamma$ decays are obtained via integration of the ${|{{\mathfrak{M}}}_{f}|}^{2}$ expressions from Eqs. (39)–(46) over the *s* and *s*_12_ variables with the ${{{{{{\rm{T}}}}}}}_{{{{{{\rm{c}}}}}}{{{{{\rm{c}}}}}}}^{+}$ amplitude squared, ${\left|{{{{{{\mathcal{A}}}}}}}_{{{{{{\rm{U}}}}}}}\left(s\right)\right|}^{2}$, from Eq. (47):

67 $${R}_{f}\left({s}_{23}\right)\equiv \int\limits_{{\left({m}_{1}+\sqrt{{s}_{23}}\right)}^{2}}^{+\infty }ds\,{\left|{{{{{{\mathcal{A}}}}}}}_{{{{{{\rm{U}}}}}}}\left(s\right)\right|}^{2}{f}_{C}(s)\,\frac{1}{s}\int\limits_{{s}_{12}^{-}(s,{s}_{23})}^{{s}_{12}^{+}(s,{s}_{23})}d{s}_{12}\,{\left|{{\mathfrak{M}}}_{f}(s,{s}_{12},{s}_{23})\right|}^{2},$$

where the lower and upper integration limits for *s*_12_ at fixed *s* and *s*_23_ are given in Eq. (66). The function *f*_*C*_(*s*) is introduced to perform a smooth cutoff of the long tail of the ${{{{{{\rm{T}}}}}}}_{{{{{{\rm{c}}}}}}{{{{{\rm{c}}}}}}}^{+}$ profile. Cutoffs are chosen to suppress the profile for regions $\left|\sqrt{s}-{\mathfrak{m}}\right|\gg {\mathfrak{w}}$, where ${\mathfrak{m}}$ and ${\mathfrak{w}}$ are the mode and FWHM for the ${{\mathfrak{F}}}^{{{{{{\rm{U}}}}}}}(s)$ distribution. Two cutoff functions *f*_*C*_(*s*) are studied:

1. A Gaussian cutoff ${f}_{C}^{G}(s)$ is defined as

68 $${f}_{C}^{G}(s\,| \,{x}_{c},{\sigma }_{c})=\left\{\begin{array}{ll}1 &{{{{{\rm{for}}}}}}\sqrt{s}\le {x}_{c};\\ {{{{{{\rm{e}}}}}}}^{-\frac{{\left(\sqrt{s}-{x}_{c}\right)}^{2}}{2{\sigma }_{c}^{2}}} &{{{{{\rm{for}}}}}}\sqrt{s} > {x}_{c}.\end{array}\right.$$

2. A power-law cutoff ${f}_{C}^{P}(s)$ defined as

69 $${f}_{C}^{P}(s\,| \,{x}_{c},{\sigma }_{c},{\nu }_{c})=\left\{\begin{array}{ll}1 &{{{{{\rm{for}}}}}}\sqrt{s}\le {x}_{c};\\ {\left(1+\frac{1}{{\nu }_{c}}\frac{{(\sqrt{s}-{x}_{c})}^{2}}{{\sigma }_{c}^{2}}\right)}^{-\frac{{\nu }_{c}+1}{2}} &{{{{{\rm{for}}}}}}\sqrt{s} > {x}_{c}.\end{array}\right.$$

Fits to the background-subtracted D^0^D^0^*π*^+^ mass spectrum using a signal profile of the form ${{\mathfrak{F}}}^{{{{{{\rm{U}}}}}}}(s)\times {f}_{C}(s)$ show that the parameter *δm*_U_ is insensitive to the choice of cutoff function when ${x}_{c}\ge {m}_{{{{{{{\rm{D}}}}}}}^{* 0}}+{m}_{{{{{{{\rm{D}}}}}}}^{+}}$ and *σ*_*c*_≥1 MeV/*c*^2^. The power-law cutoff function ${f}_{C}^{P}(s)$ with parameters ${x}_{c}={m}_{{{{{{{\rm{D}}}}}}}^{* 0}}+{m}_{{{{{{{\rm{D}}}}}}}^{+}}$ and *σ*_*c*_ = 1 MeV/*c*^2^ is chosen. The shapes for the D^0^D^0^ and D^+^D^0^ mass distributions are defined as

70 $$\begin{array}{rcl}{F}_{{{{{{{\rm{D}}}}}}}^{0}{{{{{{\rm{D}}}}}}}^{0}}(m)&=&m{R}_{{{{{{{\rm{D}}}}}}}^{0}{{{{{{\rm{D}}}}}}}^{0}{\pi }^{+}}({m}^{2}),\end{array}$$

71 $$\begin{array}{rcl}{F}_{{{{{{{\rm{D}}}}}}}^{+}{{{{{{\rm{D}}}}}}}^{0}}(m)&=&m{R}_{{{{{{{\rm{D}}}}}}}^{+}{{{{{{\rm{D}}}}}}}^{0}{\pi }^{+}}({m}^{2})+m{R}_{{{{{{{\rm{D}}}}}}}^{+}{{{{{{\rm{D}}}}}}}^{0}\gamma }({m}^{2}).\end{array}$$

### Low-energy scattering amplitude

The unitarized Breit–Wigner amplitude is formally similar to the low-energy expansion given by Eq. (13) once the factor $\frac{1}{2}{\left|g\right|}^{2}$ is divided out

72 $${{{{{{\mathcal{A}}}}}}}_{{{{{{\rm{NR}}}}}}}^{-1}=\frac{1}{a}+r\frac{{k}^{2}}{2}-ik+{{{{{\mathcal{O}}}}}}({k}^{4}),$$

73 $$\frac{2}{{\left|g\right|}^{2}}{{{{{{\mathcal{A}}}}}}}_{{{{{{\rm{U}}}}}}}^{-1}=-\left[\xi (s)-\xi ({m}_{{{{{{\rm{U}}}}}}}^{2})\right]+2\frac{{m}_{{{{{{\rm{U}}}}}}}^{2}-s}{{\left|g\right|}^{2}}-i{\varrho }_{{{{{{\rm{tot}}}}}}}(s).$$

The function *iϱ*_tot_(*s*) matches *ik* up to a slowly varying energy factor that can be approximated by a constant in the threshold region. The proportionality factor *w* has the dimension of an inverse mass and is found by matching the decay probability to the two-body phase-space expression:

74 $$\begin{array}{r}w=\frac{24\pi }{{m}_{{{{{{{\rm{D}}}}}}}^{* +}}+{m}_{{{{{{{\rm{D}}}}}}}^{0}}}\frac{1}{{c}_{1}},\end{array}$$

where *c*_1_ is a coefficient computed in Eq. (50). The comparison of ${{{{{{\mathcal{A}}}}}}}_{{{{{{\rm{NR}}}}}}}^{-1}$ and ${{{{{{\mathcal{A}}}}}}}_{{{{{{\rm{U}}}}}}}^{-1}\times 2w/{\left|g\right|}^{2}$ that validates the matching is shown in Supplementary Fig. 9.

The inverse scattering length is defined as the value of the amplitude in Eq. (72) at the D^*+^D^0^ threshold:

75 $$\begin{array}{rc}\frac{1}{a}&=-\frac{1}{w}\left\{\left[\xi ({s}_{{{{{{\rm{th}}}}}}})-\xi ({m}_{{{{{{\rm{U}}}}}}}^{2})\right]+i{\varrho }_{{{{{{\rm{tot}}}}}}}({s}_{{{{{{\rm{th}}}}}}})\right\}.\end{array}$$

The imaginary part is fully determined by the available decay channels, while the real part depends on the constant $\xi ({m}_{{{{{{\rm{U}}}}}}}^{2})$ adjusted in the fit. The quadratic term, *k*^2^ in Eq. (72), corresponds to the linear correction in*s* since *k*^2^ = (*s* − *s*_th_)/4 for the non-relativistic case. Hence, the slope of the linear term in the ${{{{{{\mathcal{A}}}}}}}_{{{{{{\rm{U}}}}}}}^{-1}$ amplitude is related to the effective range as follows:

76 $$\begin{array}{r}r=-\frac{1}{w}\frac{16}{{\left|g\right|}^{2}}.\end{array}$$

### Mass splitting for the ${\hat{{{{{{\rm{T}}}}}}}}_{{{{{{\rm{c}}}}}}{{{{{\rm{c}}}}}}}$ isotriplet

While the degrees of freedom of the light diquark for the isoscalar ${{{{{{\rm{T}}}}}}}_{{{{{{\rm{c}}}}}}{{{{{\rm{c}}}}}}}^{+}$ state are similar to those for the ${\overline{\Lambda }}_{{{{{{\rm{c}}}}}}}^{-}$ state, for the ${\hat{{{{{{\rm{T}}}}}}}}_{{{{{{\rm{c}}}}}}{{{{{\rm{c}}}}}}}$ isotriplet (${\hat{{{{{{\rm{T}}}}}}}}_{{{{{{\rm{c}}}}}}{{{{{\rm{c}}}}}}}^{0}$,${\hat{{{{{{\rm{T}}}}}}}}_{{{{{{\rm{c}}}}}}{{{{{\rm{c}}}}}}}^{+}$,${\hat{{{{{{\rm{T}}}}}}}}_{{{{{{\rm{c}}}}}}{{{{{\rm{c}}}}}}}^{++}$) the light diquark degrees of freedom would be similar to those for the ${\overline{\Sigma }}_{{{{{{\rm{c}}}}}}}$ (anti)triplet. Assuming that the difference in the light quark masses, the Coulomb interaction of light quarks in the diquark, and the Coulomb interaction of the light diquark with the c-quark are responsible for the observed mass splitting in the Σ_c_ isotriplet, the masses for the Σ_c_ states can be written as

77 $$\begin{array}{rcl}{m}_{\Sigma_{c}^{++}}&=&{m}_{{{\Sigma }}}+{m}_{{{{{{\rm{u}}}}}}}+{m}_{{{{{{\rm{u}}}}}}}-a\,{q}_{{{{{{\rm{u}}}}}}}{q}_{{{{{{\rm{u}}}}}}}-b\,{q}_{{{{{{\rm{c}}}}}}}\left({q}_{{{{{{\rm{u}}}}}}}+{q}_{{{{{{\rm{u}}}}}}}\right),\end{array}$$

78 $$\begin{array}{rcl}{m}_{\Sigma_{c}^{+}}&=&{m}_{{{\Sigma }}}+{m}_{{{{{{\rm{u}}}}}}}+{m}_{{{{{{\rm{d}}}}}}}-a\,{q}_{{{{{{\rm{u}}}}}}}{q}_{{{{{{\rm{d}}}}}}}-b\,{q}_{{{{{{\rm{c}}}}}}}\left({q}_{{{{{{\rm{u}}}}}}}+{q}_{{{{{{\rm{d}}}}}}}\right),\end{array}$$

79 $$\begin{array}{rcl}{m}_{\Sigma_{c}^{0}}&=&{m}_{{{\Sigma }}}+{m}_{{{{{{\rm{d}}}}}}}+{m}_{{{{{{\rm{d}}}}}}}-a\,{q}_{{{{{{\rm{d}}}}}}}{q}_{{{{{{\rm{d}}}}}}}-b\,{q}_{{{{{{\rm{c}}}}}}}\left({q}_{{{{{{\rm{d}}}}}}}+{q}_{{{{{{\rm{d}}}}}}}\right),\end{array}$$

where *m*_Σ_ is a common mass parameter; the second and third terms describe the contribution from the light quark masses, *m*_u_ and *m*_d_, into the mass splitting; terms proportional to *a* describe Coulomb interactions of light quarks in the diquark; terms proportional to *b* describe the Coulomb interactions of the diquark with the c-quark; and *q*_q_ denotes the charge of the q-quark. Similar expressions can be written for the ${\hat{{{{{{\rm{T}}}}}}}}_{{{{{{\rm{c}}}}}}{{{{{\rm{c}}}}}}}$ isotriplet:

80 $$\begin{array}{rcl}{m}_{{\hat{{{{{{\rm{T}}}}}}}}_{{{{{{\rm{c}}}}}}{{{{{\rm{c}}}}}}}^{0}}&=&{m}_{{\hat{{{{{{\rm{T}}}}}}}}_{{{{{{\rm{c}}}}}}{{{{{\rm{c}}}}}}}}+{m}_{{{{{{\rm{u}}}}}}}+{m}_{{{{{{\rm{u}}}}}}}-{a}^{\prime}\,{q}_{\overline{{{{{{\rm{u}}}}}}}}{q}_{\overline{{{{{{\rm{u}}}}}}}}-{b}^{\prime}\,{q}_{{{{{{\rm{c}}}}}}{{{{{\rm{c}}}}}}}\left({q}_{\overline{{{{{{\rm{u}}}}}}}}+{q}_{\overline{{{{{{\rm{u}}}}}}}}\right),\end{array}$$

81 $$\begin{array}{rcl}{m}_{{\hat{{{{{{\rm{T}}}}}}}}_{{{{{{\rm{c}}}}}}{{{{{\rm{c}}}}}}}^{+}}&=&{m}_{{\hat{{{{{{\rm{T}}}}}}}}_{{{{{{\rm{c}}}}}}{{{{{\rm{c}}}}}}}}+{m}_{{{{{{\rm{u}}}}}}}+{m}_{{{{{{\rm{d}}}}}}}-{a}^{\prime}\,{q}_{\overline{{{{{{\rm{u}}}}}}}}{q}_{\overline{{{{{{\rm{d}}}}}}}}-{b}^{\prime}\,{q}_{{{{{{\rm{c}}}}}}{{{{{\rm{c}}}}}}}\left({q}_{\overline{{{{{{\rm{u}}}}}}}}+{q}_{\overline{{{{{{\rm{d}}}}}}}}\right),\end{array}$$

82 $$\begin{array}{rcl}{m}_{{\hat{{{{{{\rm{T}}}}}}}}_{{{{{{\rm{c}}}}}}{{{{{\rm{c}}}}}}}^{++}}&=&{m}_{{\hat{{{{{{\rm{T}}}}}}}}_{{{{{{\rm{c}}}}}}{{{{{\rm{c}}}}}}}}+{m}_{{{{{{\rm{d}}}}}}}+{m}_{{{{{{\rm{d}}}}}}}-{a}^{\prime}\,{q}_{\overline{{{{{{\rm{d}}}}}}}}{q}_{\overline{{{{{{\rm{d}}}}}}}}-{b}^{\prime}\,{q}_{{{{{{\rm{c}}}}}}{{{{{\rm{c}}}}}}}\left({q}_{\overline{{{{{{\rm{d}}}}}}}}+{q}_{\overline{{{{{{\rm{d}}}}}}}}\right),\end{array}$$

where ${m}_{{\hat{{{{{{\rm{T}}}}}}}}_{{{{{{\rm{c}}}}}}{{{{{\rm{c}}}}}}}}$ is the common mass parameter, ${q}_{\overline{{{{{{\rm{q}}}}}}}}=-{q}_{{{{{{\rm{q}}}}}}}$ and *q*_cc_ = 2*q*_c_ is the charge of a cc diquark. Using the known masses of the light quarks and Σ_c_ states^10^ and taking ${a}^{\prime}=a$ and ${b}^{\prime}=b$, the mass splitting for the ${\hat{{{{{{\rm{T}}}}}}}}_{{{{{{\rm{c}}}}}}{{{{{\rm{c}}}}}}}$ isotriplet is estimated to be

83 $$\begin{array}{rcl}{m}_{{\hat{{{{{{\rm{T}}}}}}}}_{{{{{{\rm{c}}}}}}{{{{{\rm{c}}}}}}}^{0}}-{m}_{{\hat{{{{{{\rm{T}}}}}}}}_{{{{{{\rm{c}}}}}}{{{{{\rm{c}}}}}}}^{+}}&=&-5.9\pm 1.3{{{{{\rm{MeV}}}}}}/{c}^{2},\end{array}$$

84 $$\begin{array}{rcl}{m}_{{\hat{{{{{{\rm{T}}}}}}}}_{{{{{{\rm{c}}}}}}{{{{{\rm{c}}}}}}}^{++}}-{m}_{{\hat{{{{{{\rm{T}}}}}}}}_{{{{{{\rm{c}}}}}}{{{{{\rm{c}}}}}}}^{+}}&=&-7.9\pm 1.0{{{{{\rm{MeV}}}}}}/{c}^{2}.\end{array}$$

The validity of this approach is tested by comparing the calculated mass splitting between Σ*bp* and Σ*bm* states of −6.7 ± 0.7 MeV/*c*^2^ with the measured value of −5.1 ± 0.2 MeV/*c*^2^^10^. Based on the small observed difference, an additional uncertainty of 0.8 MeV/*c*^2^ is added in quadrature to the results from Eqs. (83) and (84), and finally one gets

85 $${m}_{{\hat{{{{{{\rm{T}}}}}}}}_{{{{{{\rm{c}}}}}}{{{{{\rm{c}}}}}}}^{0}}-{m}_{{\hat{{{{{{\rm{T}}}}}}}}_{{{{{{\rm{c}}}}}}{{{{{\rm{c}}}}}}}^{+}}=-5.9\pm 1.5{{{{{\rm{MeV}}}}}}/{c}^{2},$$

86 $${m}_{{\hat{{{{{{\rm{T}}}}}}}}_{{{{{{\rm{c}}}}}}{{{{{\rm{c}}}}}}}^{++}}-{m}_{{\hat{{{{{{\rm{T}}}}}}}}_{{{{{{\rm{c}}}}}}{{{{{\rm{c}}}}}}}^{+}}=-7.9\pm 1.3{{{{{\rm{MeV}}}}}}/{c}^{2}.$$

These results agree with the assigned uncertainty with results based on a more advanced model from Ref. ^147^.

## Supplementary information

Supplementary information

Editorial Assessment Report

## Data Availability

LHCb data used in this analysis will be released according to the LHCb external data access policy, which can be downloaded from https://opendata.cern.ch/record/410/files/LHCb-Data-Policy.pdf. The raw data in all of the figures of this manuscript can be downloaded from https://cds.cern.ch/record/2780001, where no access codes are required. In addition, the unbinned background-subtracted data, shown in Figs. 1, 3 and 4 have been added to the HEPData record at https://www.hepdata.net/record/ins1915358.

## Code availability

LHCb software used to process the data analysed in this manuscript is available at GitLab repository. The specific software used in data analysis is available at Zenodo repository 10.5281/zenodo.5595937.
