# Supplementary material for: Study of the doubly charmed tetraquark Tcc+
Source: Nat Commun. 2022 Jun 16;13:3351. doi: 10.1038/s41467-022-30206-w (PMC9203551; doi:10.1038/s41467-022-30206-w)
Supplement: Supplementary file 1 — Supplementary information [file 41467_2022_30206_MOESM1_ESM.pdf]

Supplementary Information: Study of the doubly charmed tetraquark T_{cc}^{+}

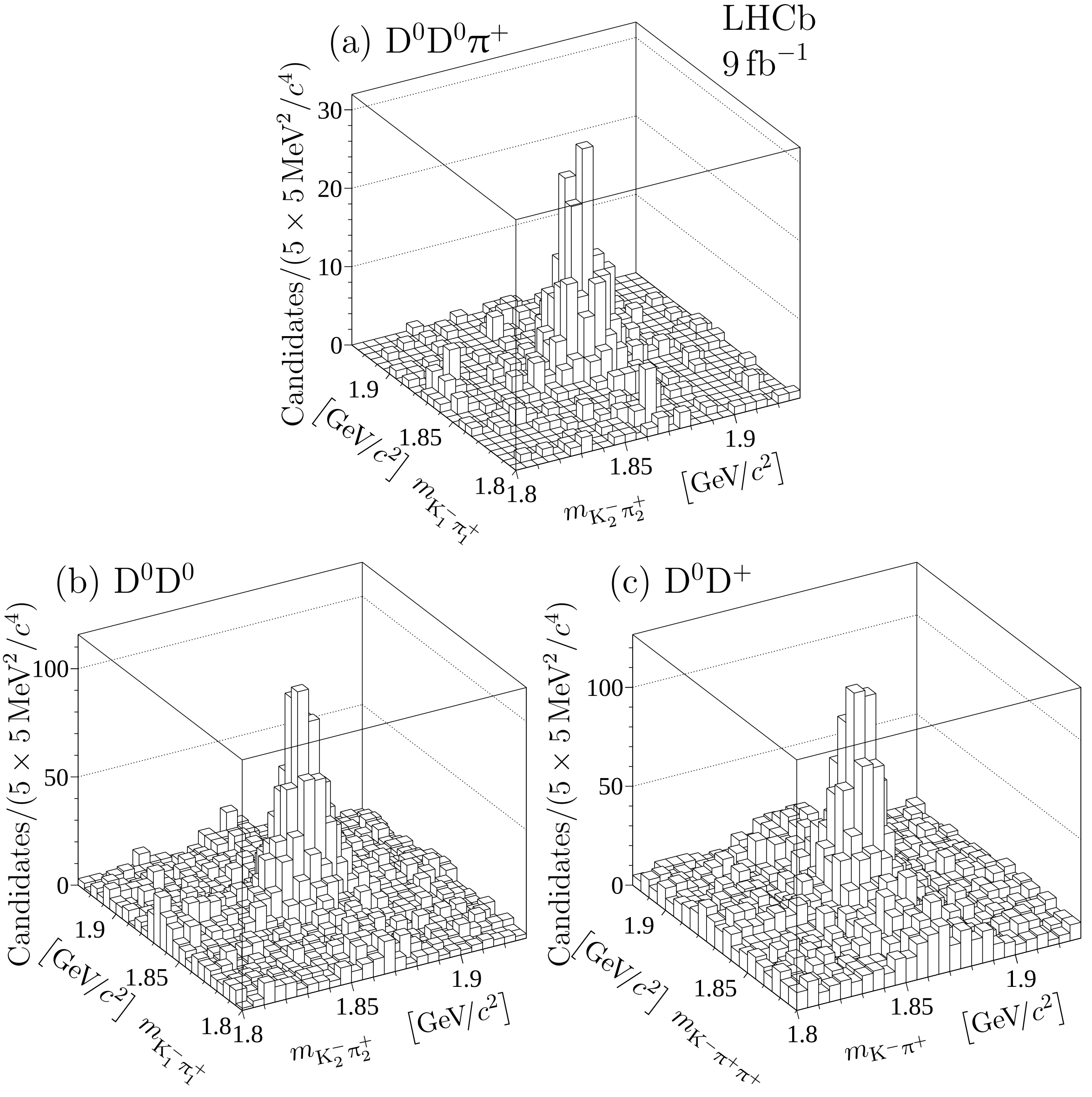

Supplementary Fig. 1: **Two-dimensional mass distributions for selected $D^0D^0\pi^+$, D^0D^0 and D^0D^+ combinations.** Two-dimensional mass distributions for D^0 and D^+ candidates from selected (a) $D^0D^0\pi^+$, (b) D^0D^0 and (c) D^0D^+ combinations. $K_{1/2}$ and $\pi_{1/2}$ correspond to daughters of the first/second D^0 candidate in D^0D^0 and $D^0D^0\pi^+$ final states.

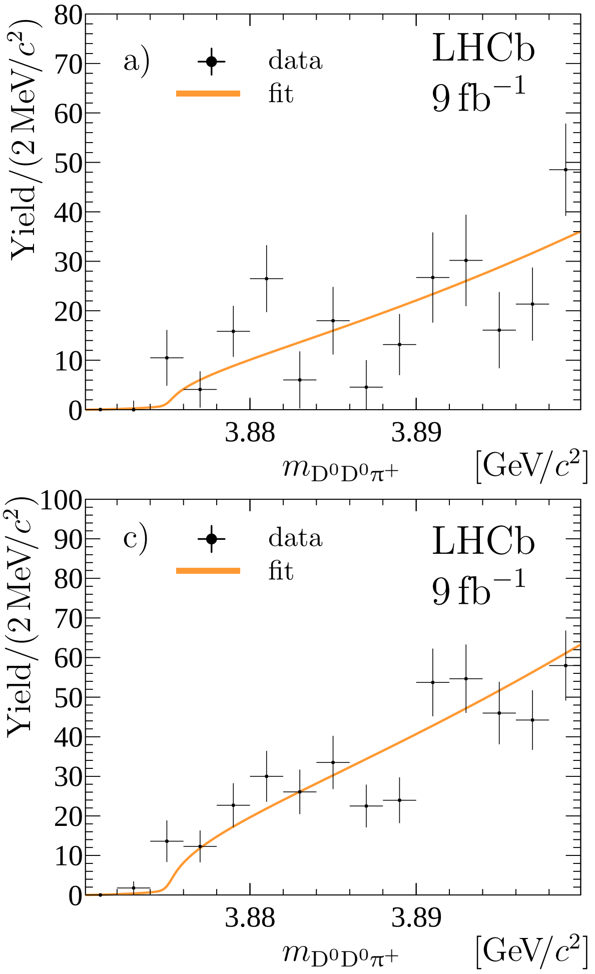

Supplementary Fig. 2: **Mass distributions for $\mathbf{D^0D^0\pi^+}$ combinations with fake $\mathbf{D^0}$ candidates.** Mass distributions for $D^0D^0\pi^+$ combinations with (a) one true and one fake D^0 candidate, (b) two fake D^0 candidates and (c) at least one fake D^0 candidate. Results of the fits with background-only functions are overlaid. Uncertainties on the data points are statistical only and represent one standard deviation, calculated as a sum in quadrature of the assigned weights from the background-subtraction procedure.

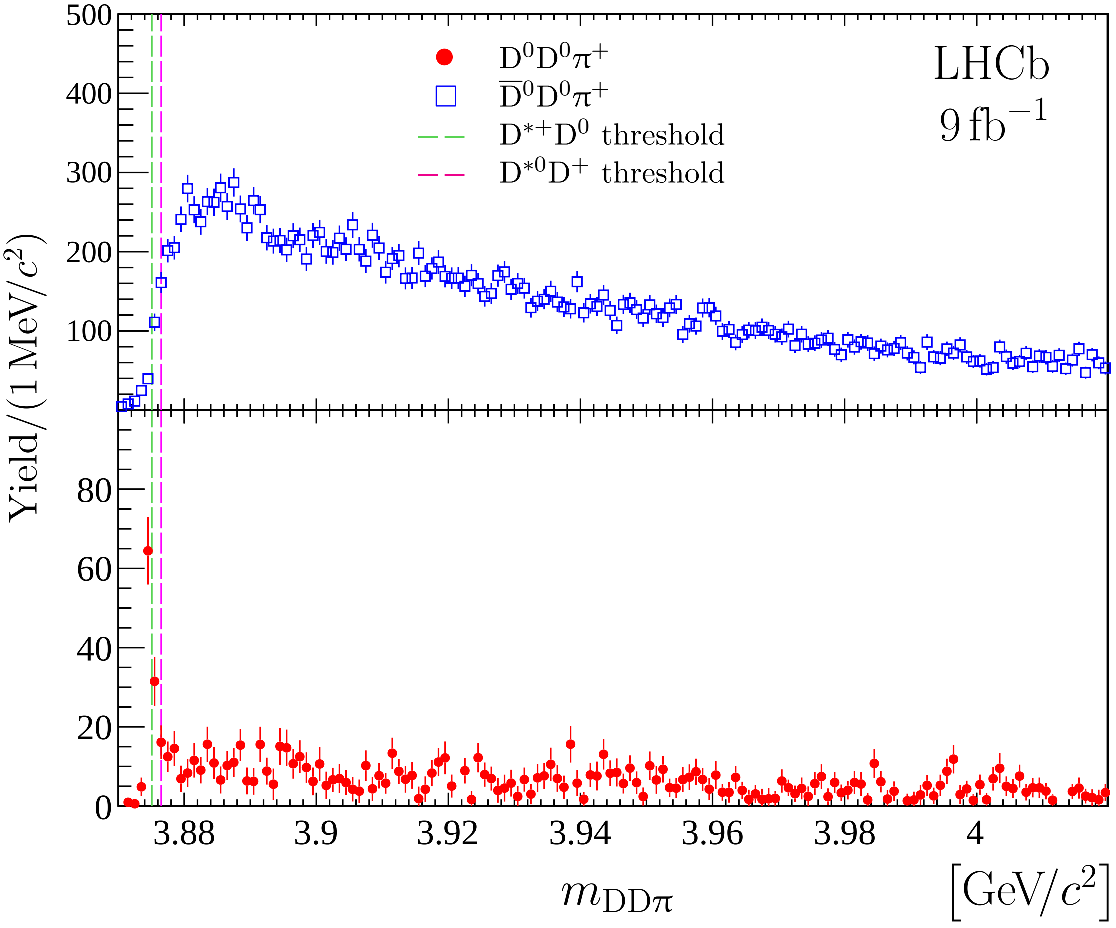

Supplementary Fig. 3: **Mass distributions for $D^0D^0\pi^+$ and $D^0\overline{D}^0\pi^-$ candidates.** Background-subtracted $D^0D^0\pi^+$ and $D^0\overline{D}^0\pi^-$ mass distributions. Uncertainties on the data points are statistical only and represent one standard deviation, calculated as a sum in quadrature of the assigned weights from the background-subtraction procedure.

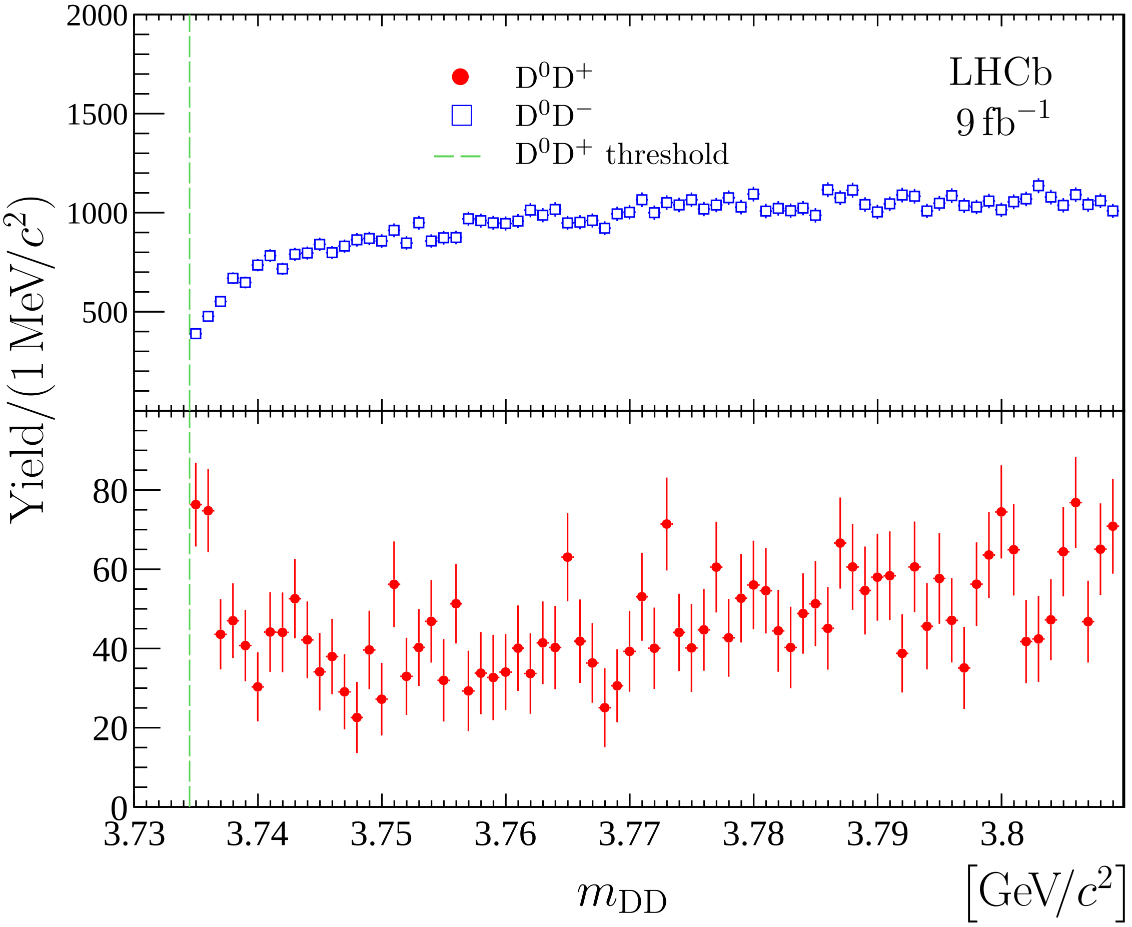

Supplementary Fig. 4: Mass distributions for D^0D^+ and D^0D^- candidates. Background-subtracted D^0D^+ and D^0D^- mass distributions. Uncertainties on the data points are statistical only and represent one standard deviation, calculated as a sum in quadrature of the assigned weights from the background-subtraction procedure.

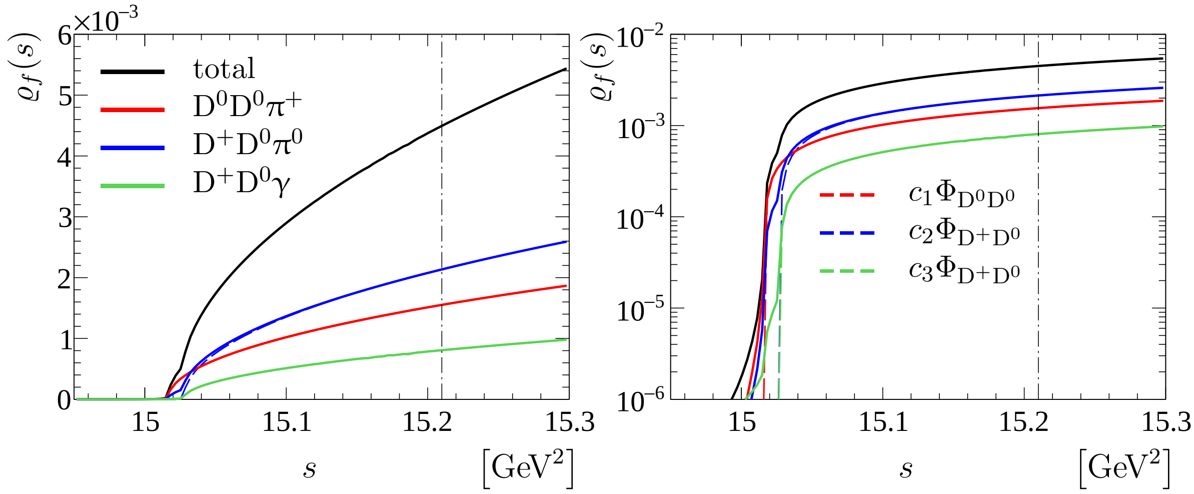

Supplementary Fig. 5: **Three-body phase-space functions $\varrho_f(s)$.** Three-body phase-space functions $\varrho_f(s)$ with (left) linear and (right) logarithmic vertical-axis scale: (red) $T_{cc}^+ \to D^0D^0\pi^+$, (blue) $T_{cc}^+ \to D^+D^0\pi^0$ and (green) $T_{cc}^+ \to D^+D^0\gamma$. The sum, $\varrho_{tot}(s)$, is shown with a black line. The two-body D^*D phase-space shapes are shown by the dashed lines and are different from the $\varrho_f(s)$ functions only in the vicinity of the thresholds or below them. Vertical dash-dotted line indicates $\sqrt{s^*} = 3.9\,$GeV.

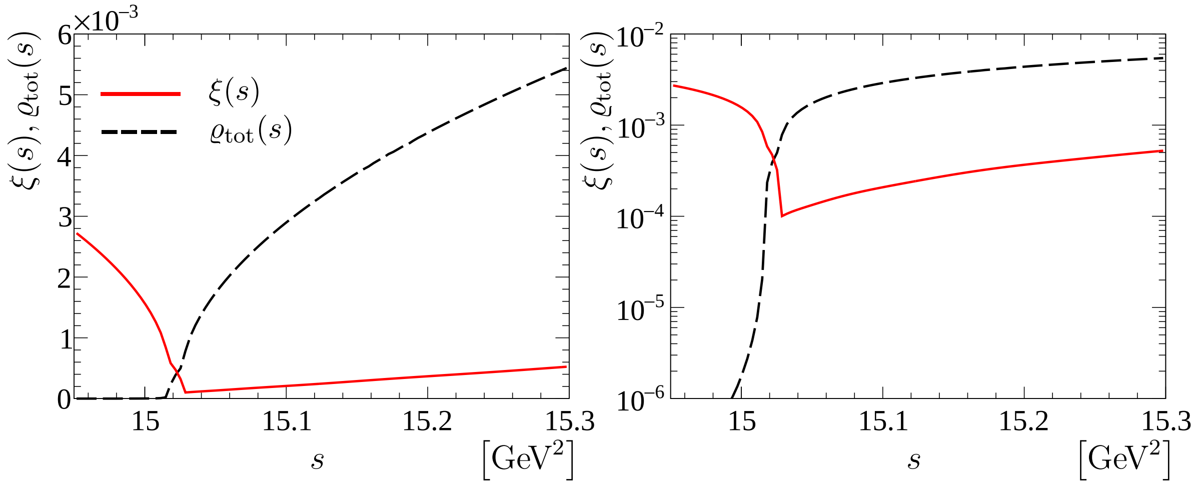

Supplementary Fig. 6: **Function $\xi(s)$.** Function $\xi(s)$ with (left) linear and (right) logarithmic vertical-axis scale is shown with a red line. The three-body phase-space function $\varrho_{tot}(s)$ is shown for comparison with a dashed line.

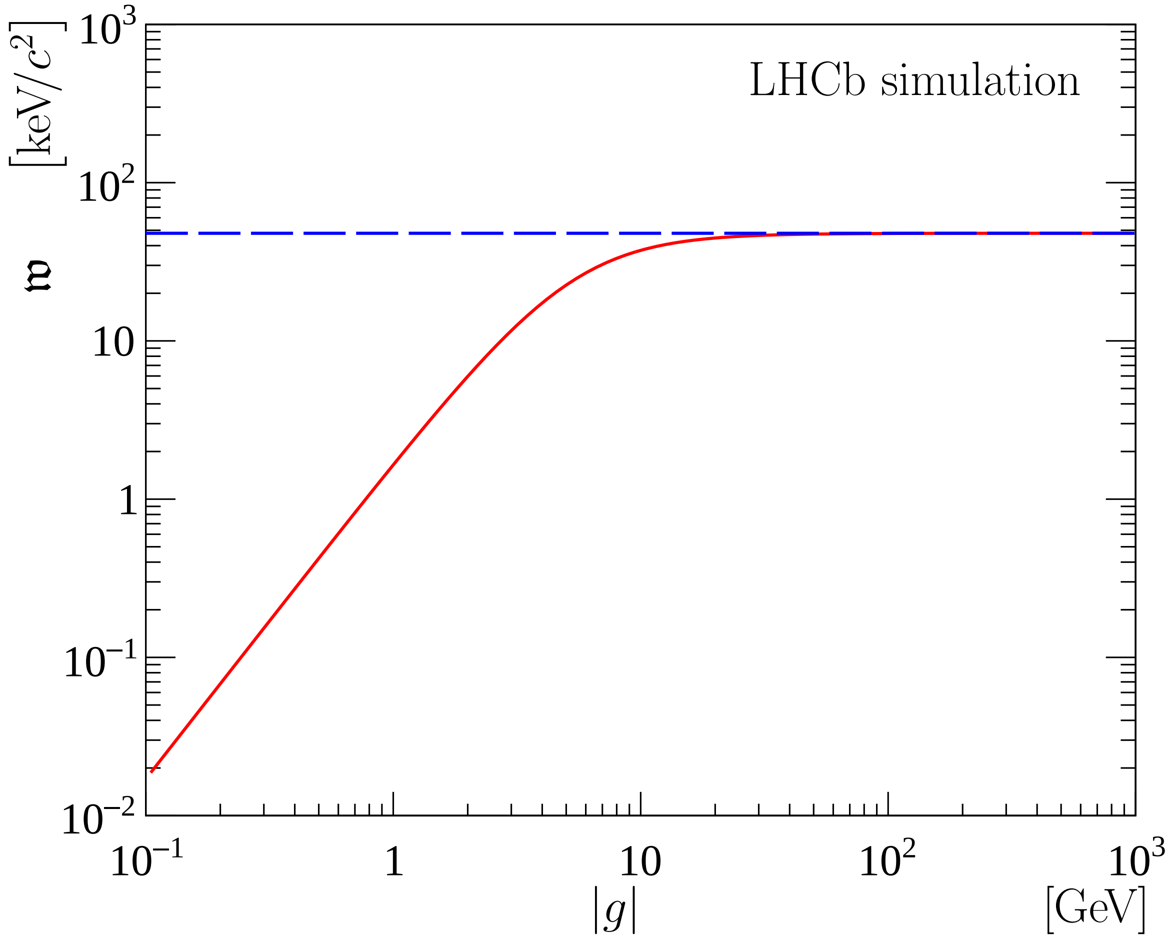

Supplementary Fig. 7: **Scaling behaviour of the $\mathfrak{F}^{\mathrm{U}}$ profile.** The full width at half maximum $\mathfrak{w}$ as a function of the $|g|$ parameter for a fixed value of the δm_{U} parameter $\delta m_{\mathrm{U}} = -359\,\mathrm{keV}/c^2$. The horizontal dashed blue line indicates the value of $\mathfrak{w}$ corresponding to the best fit parameters.

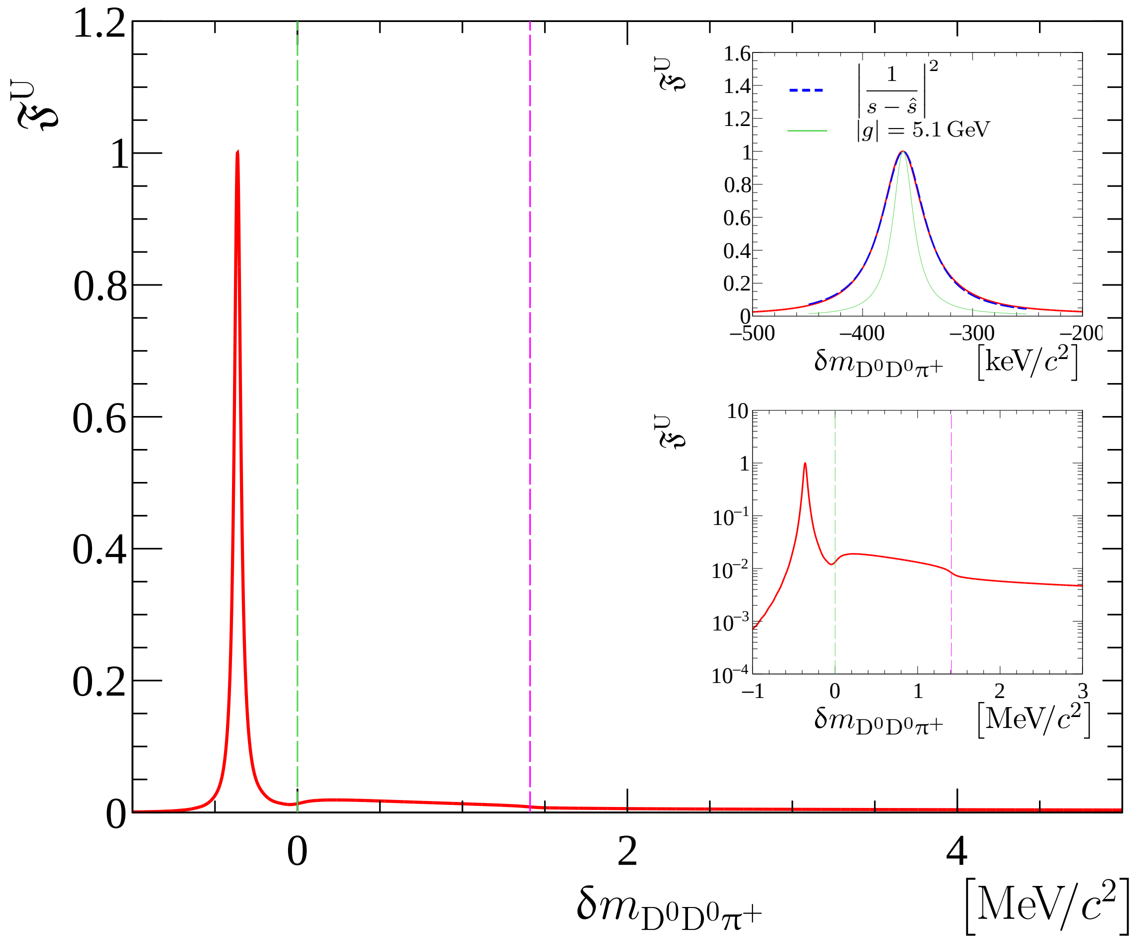

Supplementary Fig. 8: **Unitarised three-body Breit–Wigner function $\mathfrak{F}^{\mathrm{U}}$.** Unitarised three-body Breit–Wigner function $\mathfrak{F}^{\mathrm{U}}$ for $\mathrm{T}_{\mathrm{cc}}^{+} \to \mathrm{D}^0\mathrm{D}^0\pi^+$ decays (red line) for a large value of the $|g|$ parameter and $\delta m_{\mathrm{U}} = -359\,\mathrm{keV}/c^2$, normalized to unity for $\delta m_{\mathrm{D^0D^0\pi^+}} = \delta m_{\mathrm{U}}$. Top inset shows a zoomed region with overlaid (blue dashed line) *single-pole* profile with $\sqrt{\hat{s}} = \mathfrak{m} - \frac{i}{2}\mathfrak{w}$, and (thin green line) three-body Breit–Wigner profile with $|g| = 5.1\,\mathrm{GeV}$. Bottom inset shows the $\mathfrak{F}^{\mathrm{U}}$ profile in log-scale. Vertical dashed lines indicate (left-to-right) $\mathrm{D}^{*+}\mathrm{D}^0$ and $\mathrm{D}^{*0}\mathrm{D}^+$ mass thresholds.

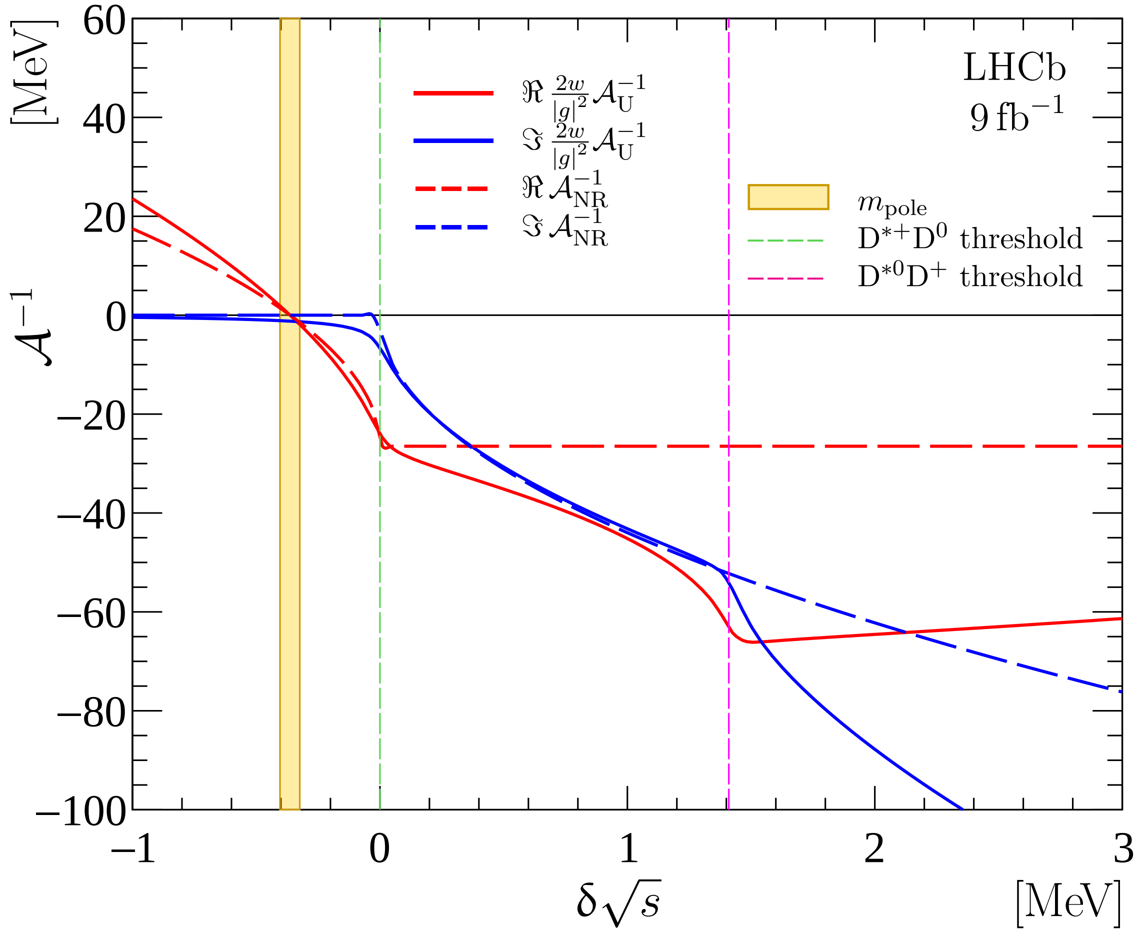

Supplementary Fig. 9: **Comparison of the $\mathcal{A}_{\mathrm{U}}$ and $\mathcal{A}_{\mathrm{NR}}$ amplitudes.** The real and imaginary parts of the inverse $\mathcal{A}_{\mathrm{U}}$ and $\mathcal{A}_{\mathrm{NR}}$ amplitudes. The yellow band correspond to the pole position and vertical dashed lines show the $\mathrm{D}^{*+}\mathrm{D}^0$ and $\mathrm{D}^{*0}\mathrm{D}^+$ mass thresholds.

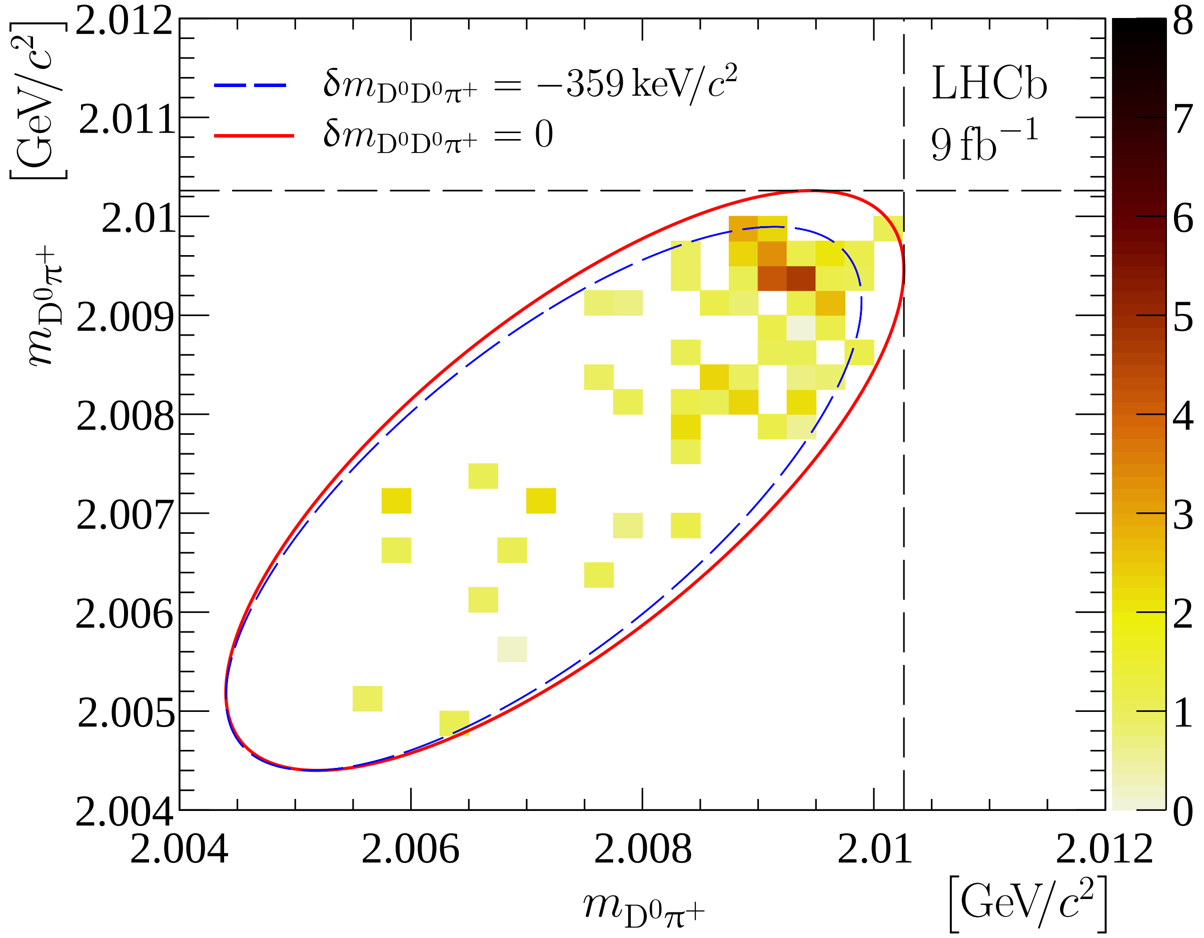

Supplementary Fig. 10: Two-dimensional $D^0\pi^+$ mass distribution. Background-subtracted two-dimensional $D^0\pi^+$ mass distribution for the $D^0D^0\pi^+$ events with $\delta m_{D^0D^0\pi^+} \leq 0$. Dashed vertical and horizontal lines indicate the known D^{*+} mass. Red and dashed blue lines show the boundary corresponding to $\delta m_{D^0D^0\pi^+} = 0$ and $\delta m_{D^0D^0\pi^+} = -359\,\text{keV}/c^2$, respectively.

Propagation matrix G

The propagation matrix G describes the $D^*D \to D^*D$ rescattering via the virtual loops including the one-particle exchange process and expressed in a symbolical way as

$G =$ 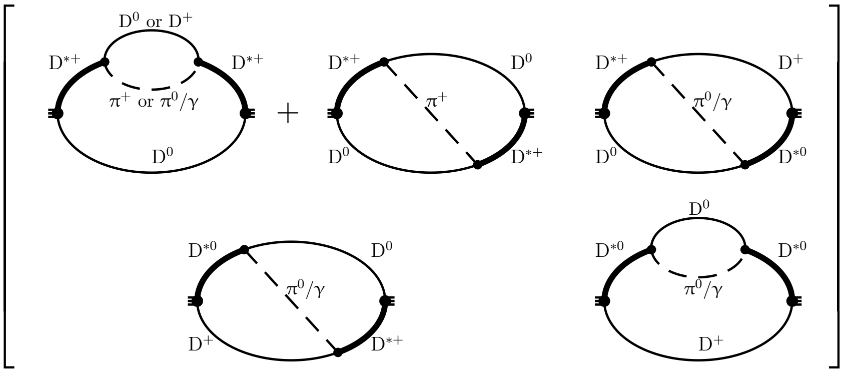 , (1)

where suppressed $D^{*0} \to D^+\pi^-$ transition is neglected.
