# Supplementary material for: Study of the doubly charmed tetraquark Tcc+
Source: Nat Commun. 2022 Jun 16;13:3351. doi: 10.1038/s41467-022-30206-w (PMC9203551; doi:10.1038/s41467-022-30206-w)
Supplement: Supplementary file 2 — Editorial Assessment Report [file 41467_2022_30206_MOESM2_ESM.pdf]

nature portfolio

GUIDED OPEN ACCESS

nature physics | nature communications | communications physics

Contents of this report

1. **Manuscript details**: overview of your manuscript and the editorial team.
2. **Review synthesis**: summary of the reviewer reports provided by the editors.
3. **Editorial evaluations**: personalized evaluation and recommendation from all 3 journals.
4. **Annotated reviewer comments**: the referee reports with comments from the editors.
5. **Open research evaluation**: advice for adhering to best reproducibility practices.

About the editorial process

Because you selected the **Nature Portfolio Guided Open Access** option, your manuscript was assessed for suitability in three of our titles publishing high-quality work across the spectrum of physics research: ***Nature Physics***, ***Nature Communications***, **and *Communications Physics***. More information about Guided Open Access can be found here.

Collaborative editorial assessment

Your editorial team discussed the manuscript to determine its suitability for the Nature Portfolio Guided OA pilot. Our assessment of your manuscript takes into account several factors, including whether the work meets the **technical standard** of the Nature Portfolio and whether the findings are of **immediate significance** to the readership of at least one of the participating journals in the Nature Portfolio Guided Open Access physics cluster.

Peer review

Experts were asked to evaluate the following aspects of your manuscript:

- **Novelty** in comparison to prior publications;
- **Likely audience** of researchers in terms of broad fields of study and size;
- **Potential impact** of the study on the immediate or wider research field;
- **Evidence** for the claims and whether additional experiments or analyses could feasibly strengthen the evidence;
- **Methodological detail** and whether the manuscript is reproducible as written;
- Appropriateness of the **literature review**.

Editorial evaluation of reviews

Your editorial team discussed the potential suitability of your manuscript for each of the participating journals. They then discussed the revisions necessary in order for the work to be published, keeping each journal's specific editorial criteria in mind.

Journals in the Nature portfolio will support authors wishing to transfer their reviews and (where reviewers agree) the reviewers' identities to journals outside of Springer Nature.
If you have any questions about review portability, please contact our editorial office at guidedoa@nature.com.

Manuscript details

Tracking number	Submission date	Decision date	Peer review type
GUIDEDOA-21-00252	Sep 7, 2021	Oct 20, 2021	Single-blind

Manuscript title	Author details
Study of the doubly charmed tetraquark T_{cc}^+<br><br>**Preprint:** https://arxiv.org/abs/2109.01056	Ivan Belyaev *et al.* (LHCb Collaboration)<br><br>**Affiliation:** Institute For Theoretical and Experimental Physics/ITEP NRC (complete author list in manuscript)

Editorial assessment team

**Primary editor**	**Marco Bentivegna**<br>**Home journal:** *Nature Communications*<br>**ORCID:** orcid.org/0000-0002-3986-5760<br>**Email:** marco.bentivegna@nature.com
**Other editors consulted**	**Stefanie Reichert**<br>**Home journal:** *Nature Physics*<br><br>**Elena Belsole**<br>**Home journal:** *Communications Physics*<br>**ORCID:** orcid.org/0000-0001-6196-5277
**About your primary editor**	Marco completed his MS in particle physics and his PhD in quantum information and quantum optics in Sapienza University of Rome. For his doctoral research, he worked on quantum computation and simulation using integrated photonic circuits. His post-doctoral appointment involved performing experiments on quantum nonlocality and contextuality. He handles manuscripts in quantum information, computation and communication, fundamental aspects of quantum theory, information theory and high energy physics. Marco is based in the London office.

Editorial assessment and review synthesis

**Editor's summary and assessment**	The manuscript provides further analysis of the proton-proton collision data collected by the LHCb apparatus (reported in a companion paper), compatible with production of a doubly charmed tetraquark state. Its aim is to characterise the state's properties in order to shed light on its nature. The analysis supports interpretation of the state as an isoscalar doubly charmed tetraquark with quantum numbers 1^+. Such a conclusion, if confirmed, would be of high interest to the particle physics community.
**Editorial synthesis of reviewer reports**	Two of the reviewers are very positive and recognise the importance of the work for the particle physics community. Of these two, reviewer #1 admits that the conclusions of the paper rest on a model, which however shouldn't diminish the significance of the study.<br><br>Reviewer #2 is the most critical. They appreciate the originality of the results and their interest to a specialised community, but they challenge the strength of the interpretation at this stage. This, in the reviewer's opinion, diminishes the significance of the results and their interest to a wider community. However, as *Nature Communications* aims at publishing research which is of interest to specialists within each field, we do not believe this should necessarily stand in the way for publication in *Nature Communications*, as long as the technical concerns from the reviewer are properly answered and the underlying assumptions and limitations of the study are clearly stated in the paper.

Editorial evaluations

Nature Physics<br><br>Revision not invited	As Nature Physics is inviting a resubmission of the companion paper, we are unable to consider a revision of this paper.
Nature Communications<br><br>Some additional data needed, but generally minor revisions	The authors should answer all the technical concerns raised by the reviewers. This will require providing additional details on the analysis as requested. Additionally, any further data or analysis that might be able to improve the solidity of the conclusions, as from reviewer #2's report, would be of great value; in their absence, a clear indication of the underlying assumptions and the limitations of the study should be added in the relevant parts of the text.
Communications Physics<br><br>Minor revisions	Similarly to the requirements for publication in *Nature Communications,* the authors should address all technical concerns raised by the reviews. No additional data or analysis will be required for publication in *Communications Physics*, but authors are encouraged to highlight any limitations that are intrinsic to the current data and their analysis.

Next steps

**Editorial recommendation 1:**	Our recommendation is to revise and resubmit your manuscript to *Nature Communications.* All the technical concerns and requests for clarifications should be answered. If the authors consider this as a feasible option, they may consider adding further data or analysis in order to strengthen the main conclusions against the limitations pointed out by reviewer #2. If this is not possible, it is essential that such limitations are clearly stated in the text.
**Editorial recommendation 2:**	You may also choose to revise and resubmit your manuscript to *Communications Physics.* Technical concerns raised by the reviewers should be addressed and clarified. Anything that is not fully supported by the current data should be made explicit, but no further data or data analysis is required.
**Note**	As stated on the previous page *Nature Physics* is not inviting a revision at this time. Please keep in mind that the journal will not be able to consider any appeals of their decision through Guided Open Access.

Revision

To follow our recommendation, please upload the revised manuscript files using **the link provided in the decision letter**.

Revision checklist

- ☐ Cover letter, stating to which journal you are submitting
- ☐ Revised manuscript
- ☐ Point-by-point response to reviews
- ☐ Updated Reporting Summary and Editorial Policy Checklist
- ☐ Supplementary materials (if applicable)

Submission elsewhere

If you choose not to follow our recommendations, you can still take the reviewer reports with you.

Option 1: Transfer to another Nature Portfolio journal
Springer Nature provides authors with the ability to transfer a manuscript within the Nature Portfolio, without the author having to upload the manuscript data again. To use this service, **please follow the transfer link provided in the decision letter.** If no link was provided, please contact guidedOA@nature.com.

> *Note that any decision to opt in to In Review at the original journal is not sent to the receiving journal on transfer. You can opt in to In Review at receiving journals that support this service by choosing to modify your manuscript on transfer.*

Option 2: Portable Peer Review option for submission to a journal outside of Nature Portfolio
If you choose to submit your revised manuscript to a journal at another publisher, we can share the reviews with another journal outside of the Nature Portfolio if requested. You will need to request that

the receiving journal office contacts us at guidedOA@nature.com.
We have included editorial guidance below in the reviewer reports and open research evaluation to aid in revising the manuscript for publication elsewhere.

Annotated reviewer reports

The editors have included some additional comments on specific points raised by the reviewers below, to clarify requirements for publication in the recommended journal(s). However, please note that all points should be addressed in a revision, even if an editor has not specifically commented on them.

Reviewer #1 information	
**Expertise**	Theoretical particle and nuclear physics, XYZ states
**Editor's comments**	This reviewer's report confirms the value of the study in spite of its limitations, and was key in helping the editors gauging the importance of the criticisms raised by reviewer #2.

Reviewer #1 comments	
**Section**	**Annotated Reviewer Comments**
**Remarks to the Author: Overall significance**	This paper is very significant: it presents a careful, first and original study of the properties of the doubly charged tetraquark newly discovered by the same collaboration, discover presented in another paper.<br>This tetraquark state is the first exotic of this type with an unprecedented long lifetime and its study is certainly very significant both for the strong interaction community and for the wider field.<br><br>I believe that the authors have extensively quoted previous work even if it may be beneficial to quote some reviews on the subject like the ones of the quarkonium working group.
**Remarks to the Author: Impact**	N/A
**Remarks to the Author: Strength of the claims**	This paper presents a very accurate and detailed study of the properties of this newly discovered state. The quantum numbers are thoroughly investigated as well as the decay structure of the state. The obtained results suggest that the state is an isoscalar.<br><br>Under this hypothesis, properties of the state like the mass relative to the first

	threshold and the coupling to it are analysed in a unitarized model, with characteristics similar to what is widely used in the literature. While this is a model, it is also clear that we do not yet have any way to obtain these state properties directly from the underlying field theory, and therefore this study is highly useful and valuable.<br><br>Moreover all the assumptions are clearly described as well a big effort is made to try to quantify the systematics. The information obtained on the pole position, the scattering length, the effective range and the compositeness will drive many future experimental and theoretical confirmations and explorations. The observation on the characteristics of the production rate of the state with respect to the track multiplicity is pretty interesting and will motivate many studies.
**Remarks to the Author: Reproducibility**	I believe that the statistical analysis, the quality of the data and of the presentation are appropriate.

Reviewer #2 information

**Expertise**	Experimental particle physics, XYZ states with a focus on tetraquarks
**Editor's comments**	This report is the most critical. While considerations about suitability for a specific journal are the prerogative of editors, specific technical concerns should be answered with as much detail as possible.

Reviewer #2 comments

Section	Annotated Reviewer Comments
**Remarks to the Author: Overall significance**	This manuscript present a detailed study for the recently observed Tcc state by the LHCb collaboration (arXiv:2109.01038). The results in my opinion, original but not significant, and they are only of interest to a special filed instead of a wider community. The most important conclusion is that the Tcc might be a hadronic molecule. However, the conclusion itself are not solid enough. The multiplicity dependent production yield of Tcc is different from molecule expectation, and the D0D*+ intermediate state need further supporting studies. The authors have credit previous work properly.<br><br>**Please try to give possible explanations for this phenomenology.**
**Remarks to the**	I do not think the paper will influence our thinking in the field.

**Author: Impact**	
**Remarks to the Author: Strength of the claims**	Here I listed a few questions and comments, which can strengthen the conclusions. However, I am not sure whether these can be addressed by the LHCb experiment.<br><br>1. Page 4, last paragraph: It's difficult to understand what was doing here corresponding to the numbers in Table 4.<br>2. Page 6, parametrization of background: when you use a 3-body phase space background shape, I assume your signal significance will be reduced. Please give these details here.<br>3. Page 8, Discussion, Line 7: may you give the resolution of D*+ here, which is quite relevant to your \delta m measurement?<br>4. Page 8, Discussion, paragraph 2: From Extended Data Fig. 10, I think it's difficult to make the conclusion that D0pi come from D*+. It's quite possible there are D0D0 resonance too, as from the Dalitz plot. From the current analysis, it's hard to judge whether the intermediate state come from D0D0 resonance or D*0 resonance.<br>5. Page 9, paragraph 1: The discussion about D+D*0 seems lack of enough supporting, without reconstruction of pi^0 or gamma. It's difficult to convince the audience the D+D0 peak come from D+D*0 resonance.<br><br>**Please either provide additional evidence against these criticisms or, if this is not possible, acknowledge the highlighted limitations in the text.**<br><br>6. Page 10, table 6: Here you observed 263 D0D0 events from the peak, while the Tcc events is only 186, which means the D0D0 peak not only come from Tcc, but also from other sources, e.g. Tcc^0 etc. As for the mass and yield prediction about the isospin partners, it suffers from theoretical uncertainties, and a ~1.5 times yield of D0D0 peak events might be a strong hint.<br>7. Page 14, last paragraph: a) Is it possible to prove the D0D0bar mass enhancement come from chi_c1(3872)? By only looking at the D0D0bar mass distribution it's difficult to judge where does it come from. b) Further, according to LHCb's measurement, the intrinsic width of chi_c1(3872) is about 1 MeV, which is comparable to the resolution here, and thus will not make the D0D0bar mass distribution significantly wide. c) As for chi_c1(3872)->D0D0bar pi0/gamma decay, both Belle and BESIII have studied it, and the information is already quite abundant.<br>8. Page 18, paragraph 2: Is there any challenge to reconstruct D0->K3pi channel at the moment?<br><br>**Please include the requested technical details, and perform the requested analysis where possible.**
**Remarks to the**	The statistical analysis seems to be appropriate and all the details of how the

**Author: Reproducibility**	results have been achieved have been givenThe presentation of the manuscript is almost clear, after some necessary modifications (see my comments above). It's difficult to reproduce the work. However, in order to strengthen the claims that Tcc decay are dominated by D* component and there is no isospin partner, it's important for other experiments such as Belle/Belle II to reproduce the D+D*0 decay mode.<br>**Although a confirmation from other experiments would be desirable, we believe that the lack thereof does not diminish the case for publication.**

Reviewer #3 information

**Expertise**	Experimental particle physics; broader but also tetraquarks
**Editor's comments**	The report is clearly positive, and the few requests for additional details and clarifications should not be a problem for the authors.

Reviewer #3 comments

Section	Annotated Reviewer Comments
**Remarks to the Author: Overall significance**	The main result, Study of the doubly charmed tetraquark T+cc, is of appropriate importance and significance to be published.<br><br>The result is novel and has not been made prior to this measurement and all appropriate previous measurements have been cited.<br><br>The claims are supported and well motivated as well as being based on the measurements made in this paper.
**Remarks to the Author: Impact**	The clarity of presentation and measurement of the properties of the new state will support theoretical development in the area of exotic hadrons.
**Remarks to the Author: Strength of the claims**	The claims are well supported in the paper.<br><br>I have a few minor issues that should be addressed about some of the claims.<br><br>1. Bottom page 4 through 5. The claim is made that the FWHM for the two signal models used in the analysis are consistent. I have read through these paragraphs a few times and have a feel for what is being attempted here but I still cannot figure out exactly what is being done or claimed. This section needs to be rewritten to clarify exactly how these quantities are consistent, a sketch

	or cartoon may be of assistance.<br><br>2. Figure 3: Has a legend that shows a background contribution where the fit results in a negligible background contribution. This makes the plot confusing, at.a minimum a comment needs to be added to the legend to explain this.<br><br>3. Fig 9. The top plot contains a suppressed zero which has not been commented on or signified in the axis. Either add a comment or explicitly add a signifier to show the zero is suppressed .
**Remarks to the Author: Reproducibility**	The data and methods used in this analysis are presented so that an accomplished particle physics researcher could reproduce the results (in conjunction with the companion paper).

Open research evaluation

Data availability
**Data availability statement**
Thank you for including a Data Availability statement. However, we noted that you have only indicated that data are available upon request. The data availability statement must make the conditions of access to the “minimum dataset” that are necessary to interpret, verify and extend the research in the article, transparent to readers.<br><br>In addition, Nature Portfolio policies include a strong preference for research data to be archived in public repositories. For data types without specific repositories, we recommend that data are deposited in a generalist repository such as figshare or Dryad. More information about our data availability policy can be found here.<br><br>See here for more information about formatting your Data Availability Statement.
**Code availability statement**
For all studies using custom code or mathematical algorithm that is deemed central to the conclusions, a statement must also be included under the heading "Code availability", indicating whether and how the code or algorithm can be accessed, including any restrictions to access. Code availability statements should be provided as a separate section after the data availability statement but before the References.<br><br>In the Reporting Summary, you included a link to https://gitlab.cern.ch/lhcb-bandq/X2DDstar. As this link is not publicly accessible, please clarify the limitations of access in the Code Availability Statement.
**Other data requests**
Springer Nature strongly supports data sharing and believes that all datasets on which the conclusions of the paper rely should be available to readers. We encourage authors to ensure that their datasets are either deposited in publicly available repositories (where available and appropriate) or presented in the main manuscript or additional supporting files whenever possible.<br><br>Please see Springer Nature’s information on recommended repositories here.<br><br>In the Editorial Policy Checklist, you have confirmed that all relevant accession codes are provided; however, we observed that no data requiring mandated deposition was generated in the study. Further, the ‘data’ section of the reporting summary declares that ‘LHCb has an open data policy, see document LHCb-PUB-2013-003, http://cdsweb.cern.ch/record/1543410?ln=en. Subject to the resources being identified, LHCb will endeavor to provide open access to some reconstructed level data on disk at

CERN.' This link directs to the data access policy of LHCb, which
provides information on restrictions on data availability. Further, a link to access the data is not provided in the manuscript or in the reporting summary.

Please make the limitations of access more explicit in the Data Availability Statement.

All source data underlying the graphs and charts presented in the main figures must be made available as Supplementary Data (in Excel or text format) or via a generalist repository (eg, Figshare or Dryad). This is mandatory for publication in a Nature Portfolio journal, but is also best practice for publication in any venue. In the present paper, Figure 1, 3, 4a, 4b, 5a and 5b require associated source data.

Please provide a 'Competing interests' statement using one of the following standard sentences:

1. The authors declare the following competing interests: [specify competing interests]
2. The authors declare no competing interests.

See our competing interests policy for further information.

Reporting & reproducibility

Nature Portfolio journals allow unlimited space for Methods. The Methods must contain sufficient detail such that the work could be repeated. It is preferable that all key methods be included in the main manuscript, rather than in the Supplementary Information. Please avoid use of "as described previously" or similar, and instead detail the specific methods used with appropriate attribution.

Statistics and data presentation

The meaning of all error bars/bands and how they were calculated should be described within the captions of all figures in which they occur. If they represent standard deviations (or absolute minima and maxima) then this can be simply stated as such, but if not, more detail is required.

Other notes

We have included as an attachment to the decision letter a version of your Reporting Summary with a few notes. This is mainly for your information, but we hope it is helpful when preparing your revised manuscript. If you decide to resubmit the manuscript for further consideration, please be sure to include an updated Reporting Summary.
